# A comprehensive and integrative reconstruction of evolutionary history for Anomura (Crustacea: Decapoda)

**DOI:** 10.1186/1471-2148-13-128

**Published:** 2013-06-20

**Authors:** Heather D Bracken-Grissom, Maren E Cannon, Patricia Cabezas, Rodney M Feldmann, Carrie E Schweitzer, Shane T Ahyong, Darryl L Felder, Rafael Lemaitre, Keith A Crandall

**Affiliations:** 1 Department of Biology, Florida International University-Biscayne Bay Campus, North Miami, FL, 33181, USA; 2 Department of Biology, Brigham Young University, Provo, UT, 84602, USA; 3 Computational Biology Institute, George Washington University, Ashburn, VA, 20147, USA; 4 Department of Geology, Kent State University, Kent, OH, 44242, USA; 5 Kent State University at Stark, 6000 Frank Ave. NW, North Canton, OH, 44720, USA; 6 Australian Museum, 6 College St, Sydney, NSW, 2010, Australia; 7 Department of Biology, University of Louisiana at Lafayette, Lafayette, LA, 70504, USA; 8 Department of Invertebrate Zoology, National Museum of Natural History, Smithsonian Institution, 4210 Silver Hill Road, Suitland, MD, 20746, USA

**Keywords:** Anomura, Phylogeny, Divergence times, Diversification rates, Molecular, Morphology, Character evolution, Next-generation sequencing

## Abstract

**Background:**

The infraorder Anomura has long captivated the attention of evolutionary biologists due to its impressive morphological diversity and ecological adaptations. To date, 2500 extant species have been described but phylogenetic relationships at high taxonomic levels remain unresolved. Here, we reconstruct the evolutionary history—phylogeny, divergence times, character evolution and diversification—of this speciose clade. For this purpose, we sequenced two mitochondrial (16S and 12S) and three nuclear (H3, 18S and 28S) markers for 19 of the 20 extant families, using traditional Sanger and next-generation 454 sequencing methods. Molecular data were combined with 156 morphological characters in order to estimate the largest anomuran phylogeny to date. The anomuran fossil record allowed us to incorporate 31 fossils for divergence time analyses.

**Results:**

Our best phylogenetic hypothesis (morphological + molecular data) supports most anomuran superfamilies and families as monophyletic. However, three families and eleven genera are recovered as para- and polyphyletic. Divergence time analysis dates the origin of Anomura to the Late Permian ~259 (224–296) MYA with many of the present day families radiating during the Jurassic and Early Cretaceous. Ancestral state reconstruction suggests that carcinization occurred independently 3 times within the group. The invasion of freshwater and terrestrial environments both occurred between the Late Cretaceous and Tertiary. Diversification analyses found the speciation rate to be low across Anomura, and we identify 2 major changes in the tempo of diversification; the most significant at the base of a clade that includes the squat-lobster family Chirostylidae.

**Conclusions:**

Our findings are compared against current classifications and previous hypotheses of anomuran relationships. Many families and genera appear to be poly- or paraphyletic suggesting a need for further taxonomic revisions at these levels. A divergence time analysis provides key insights into the origins of major lineages and events and the timing of morphological (body form) and ecological (habitat) transitions. Living anomuran biodiversity is the product of 2 major changes in the tempo of diversification; our initial insights suggest that the acquisition of a crab-like form did not act as a key innovation.

## Background

The infraorder Anomura represents a highly diverse group of decapod crustaceans comprised of hermit crabs, mole crabs, king crabs, squat-lobsters and porcelain crabs. The fossil record contains representatives of nearly all extant families and spans the Norian/Rhaetian (Late Triassic) [1] to Holocene. Anomurans have colonized a wide variety of ecosystems including freshwater, anchialine cave, terrestrial and hydrothermal vent habitats, and are distributed from the ocean’s surface to depths more than 5000 m [2]. Their morphological and ecological diversity are of doubtless scientific interest, but anomurans also represent an important economic commodity as evident in major commercial fisheries for some king crab and squat lobster genera [3-5] and the common use of hermit crabs as pets in the aquarium trade. Moreover, some species are threatened or endangered due to rarity in nature, e.g., Pylochelidae [6], overfishing, e.g., Lithodidae [7], or habitat loss, e.g., Aeglidae [8-10]. Thus, improved understanding of these groups bears not only on appreciation of their diversity and ecology, but also strategies for their conservation.

Anomuran classification has long been fraught with controversy [see reviews by [11-13]]. Early classifications from the 19th to the first half of the 20th centuries were based on adult morphological characters including mouthparts, antennae, gills, pleon type, and/or larval characteristics. These classifications often differed in higher-level composition and, in some cases, the infraordinal name (e.g. Anomura vs. Anomala). Since these studies, various researchers have proposed changes in the classification scheme [14-18], many of which remain actively debated. More recently, molecular and/or morphological data have been used to reevaluate anomuran relationships [19-21]. As currently defined, extant Anomura contains 7 superfamilies, 20 families, 335 genera, and more than 2500 species [17,18,22,23]. Although the monophyly of Anomura is widely accepted [24-26], the elucidation of internal relationships among families, genera, and species using modern methods is dynamic and under continuous debate [11,17,18,20,23,27].

One of the most debated evolutionary questions within Anomura is phylogenetic relationships between hermit and king crabs. Since the early 1800’s [e.g., [28,29]], studies have suggested king crabs and hermit crabs are close relatives, despite first appearances to the contrary. King crabs are among the largest arthropods and have a crab-like body shape, whereas hermit crabs are relatively small and depend on a shell for protection. Despite glaring morphological differences as adults, an affinity between king crabs (lithodoids) and hermit crabs (paguroids) has been long suggested [30,31]. Although most accept this claim, the evolutionary pathways and hypothesized ancestor of both groups has been debated for decades, with two major hypotheses being proposed. The first suggests that the lithodids (*Lithodes* or *Paralithode*s) evolved from a pagurid-like ancestor (*Pagurus*) (“hermit to king hypothesis”) while the second suggests the opposite evolutionary pathway (“king to hermit hypothesis”). Here we revisit these hypotheses in light of new phylogenetic data to test the “hermit to king”/“king to hermit” evolutionary pathway.

Additional controversy over anomuran relationships stems from apparently rampant examples of convergent and/or parallel evolution in body forms. Anomurans span an impressive array of body configurations that include: 1) crab-like forms 2) squat-lobster forms 3) hermit crab forms with pleonal (abdomen) symmetry (found in 1 hermit crab family) and 4) hermit crab forms with pleonal asymmetry (found in 4 hermit crab families). Recent studies suggest that the acquisition of a crab-like body form, known as carcinization [see, [32] for a review of this concept], has occurred multiple times during evolution of the group [20,33]. Brachyura, all of which possess a “crab-like” body shape or slight modifications to this form, dominates decapod diversity with 6550+ species [34], and is considered the sister clade to Anomura [24-26,35-38]. Given the success of brachyuran crabs, it has been hypothesized that acquisition of a crab-like form may have acted as a key innovation [33], possibly impacting diversification rates within these lineages. For the first time, we explore diversification patterns in Anomura and specifically test if carcinized lineages underwent unusually rapid diversification rates. If the emergence of the crab-like form promoted diversification we would expect the overall rate in carcinized lineages to be high compared to net of diversification across Anomura. Additionally, we test if the acquisition of different body forms (i.e., crab-like, squat-lobster-like, pleonal asymmetry and symmetry (hermit)) arose once or multiple times during the emergence of the anomurans and reconstruct the evolutionary pathways of these transitions.

Divergence dating is a powerful tool used to estimate the timing and origins of diversity, morphological traits, habitat shifts, and diversification. Although nearly all the family-level groups of Anomura are represented in the fossil record, the discovery has not been as frequent as that of other decapod groups (i.e., true crabs, lobsters). Two factors, variations in cuticular sclerotization and habitat preference, are likely responsible for the limited occurrence of anomuran fossils. Many taxa are weakly calcified, whereas others possess well-calcified claws and poorly calcified carapaces and pleons. In addition, habitats currently occupied by anomurans, including freshwater, terrestrial, intertidal marine, deep marine, and hydrothermal vent areas are strongly underrepresented in the fossil record. Despite these limitations, we incorporate 31 fossil calibrations to estimate the origin of lineages and major events during anomuran evolutionary history, including the transition of body forms and shift into freshwater and terrestrial environments.

Here, we present the taxonomically broadest and largest dataset yet assembled. We combine sequences generated by traditional Sanger and next-generation 454 sequencing methods with morphological characters, including 19/20 extant families and 137 species, to estimate phylogenetic relationships, character state evolution, divergence times, and diversification patterns among major lineages of this diverse clade of crustaceans. Our comprehensive sampling, in combination with modern integrative approaches, allows us to present the most complete evolutionary picture for the infraorder Anomura to date.

## Results

Our study includes representatives from 19 of the 20 anomuran families and 18 outgroup taxa sampled across Decapoda (Dendrobranchiata, Caridea, Axiidea, Gebiidea, Brachyura) (Table 1). Alternative outgroup sampling schemes did not affect internal relationships among Anomura. The optimal models of evolution for each gene selected in MODELTEST were as follows: GTR + I + G 18S, 28S, H3 and TVM + I + G 12S, 16S. Several sequences downloaded from GenBank were excluded from the analysis due to contamination after a BLAST search and/or strange alignment results (see Additional file 1).

**Table 1:** Taxonomy, voucher catalog numbers, and GenBank accession numbers for gene sequences used in this study

**Infraorder**	**Family**	**Species**	**Catalog ID**	**16SrRNA**	**18SrRNA**	**28SrRNA**	**H3**	**12SrRNA**
**Ingroup**								
Anomura	Aeglidae	*Aegla abtao* Schmitt, 1942	KAC-Aa5/KC_Aa004	AY050067	AF439390	AY595966	DQ079658	AY050021
Anomura	Aeglidae	*Aegla alacalufi* Jara & Lopez, 1981	KACa1144/KaC798/KACa0090/KAC-A90	FJ472207	EU920958	AY595958	EU921042	AY050013
Anomura	Aeglidae	*Aegla camargoi* Buckup & Rossi, 1977	KACa0358	AY595874	N/A	AY596045	N/A	AY595493
Anomura	Aeglidae	*Aegla cholchol Jara* & Palacios, 1999	KAC-A71	AY050050	N/A	AY595948	N/A	AY050004
Anomura	Aeglidae	*Aegla jarai *Bond-Buckup & Buckup, 1994	KACa0273	AY595849	N/A	AY596020	N/A	AY595468
Anomura	Aeglidae	*Aegla papudo* Schmitt, 1942	KAC-A7	AY050032	AY595796	AY595930	N/A	AY049986
Anomura	Aeglidae	*Aegla platensis* Schmitt, 1942	KACa0495	AY595917	AY595800	AY596088	N/A	AY595536
Anomura	Aeglidae	*Aegla uruguayana* Schmitt, 1942	KACaB395	AF436051	AF436012	AF435992	N/A	AY595505
Anomura	Aeglidae	*Aegla violacea* Bond-Buckup & Buckup, 1994	KACa0379	AY595880	AY595799	AY596051	N/A	AY595499
Anomura	Albuneidae	*Albunea catherinae* Boyko, 2002	KC6848/ULLZ10315	KF182559	KF182445	KF182607	N/A	KF182439
Anomura	Albuneidae	*Albunea gibbesii* Stimpson, 1859	KC4754/ULLZ7376	KF182558	KF182440	KF182604	KF182698	KF182373
Anomura	Albuneidae	*Lepidopa californica* Efford, 1971	N/A	AF436054	AF436015	AF435996	N/A	N/A
Anomura	Albuneidae	*Lepidopa dexterae* Abele & Efford, 1972	KC6846/ULLZ4867	KF182561	KF182442	KF182606	KF182704	KF182375
Anomura	Albuneidae	*Paraleucolepidopa myops* (Stimpson, 1860)	KC4756/ULLZ10659	KF182560	KF182441	KF182605	KF182703	KF182374
Anomura	Albuneidae	*Zygopa michaelis* Holthuis, 1961	KC6849/ULLZ7565	KF182562	KF182443	KF182608	KF182699	KF182387
Anomura	Blepharipodidae	*Blepharipoda occidentalis* Randall, 1840	N/A	AF436053	AF436014	AF435994	N/A	N/A
Anomura	Chirostylidae	*Chirostylus novaecaledoniae* Baba, 1991	MNHN:Ga 2072	EU821539	EU821555	EU821572	N/A	N/A
Anomura	Chirostylidae	*Gastroptychus novaezelandiae* Baba, 1974	NIWA:23496	EU821538	EU821554	EU821571	N/A	N/A
Anomura	Chirostylidae	*Gastroptychus rogeri* Baba, 2000	NIWA:14598	HQ380260	HQ380285	HQ380272	N/A	N/A
Anomura	Chirostylidae	*Gastroptychus spinifer* (A. Milne-Edwards, 1880)	KC6839/ULLZ11351	KF182520	KF182511	KF182657	KF182720	KF182438
Anomura	Chirostylidae	*Uroptychus nitidus* (A. Milne-Edwards, 1880)	KACurni	AY595925	AF439387	AY596096	N/A	AY595544
Anomura	Chirostylidae	*Uroptychus parvulus* (Henderson, 1885)	KACurpa	AY595926	AF439386	AY596097	DQ079703	AY595545
Anomura	Chirostylidae	*Uroptychus scambus* Benedict, 1902	NIWA:10198	EU831282	EU821553	EU831283	N/A	N/A
Anomura	Chirostylidae	*Uroptychus spinirostris* (Ahyong & Poore, 2004)	NIWA:8992	N/A	EU821582	EU821570	N/A	N/A
Anomura	Coenobitidae	*Birgus latro* (Linnaeus, 1767)	KC6694	KF182532	KF182470	KF182625	KF182696	KF182421
Anomura	Coenobitidae	*Coenobita clypeatus* (Fabricius, 1787)	KC4759/ULLZ9968	KF182531	KF182467	KF182624	KF182695	KF182420
Anomura	Coenobitidae	*Coenobita compressus* (H. Milne Edwards)	N/A	AF436059	AF436023	AF435999	N/A	N/A
Anomura	Coenobitidae	*Coenobita perlatus* H. Milne Edwards, 1836	MNHN:IU200816162	HQ241512	HQ241524	HQ241535	HQ241557	HQ241501
Anomura	Diogenidae	*Areopaguristes hewatti* (Wass, 1963)	KC4766/ULLZ6876	KF182535	KF182485	KF182643	KF182733	KF182377
Anomura	Diogenidae	*Areopaguristes hewatti* (Wass, 1963)	KC6865/ULLZ6876	KF182536	KF182481	KF182644	KF182734	N/A
Anomura	Diogenidae	*Areopaguristes hewatti* (Wass, 1963)	KC6976/ULLZ6861	N/A	KF182482	KF182645	KF182735	KF182378
Anomura	Diogenidae	*Areopaguristes hummi* (Wass, 1955)	KC6866/ULLZ6880	KF182541	KF182483	KF182641	KF182730	KF182379
Anomura	Diogenidae	*Areopaguristes hummi* (Wass, 1955)	KC6984/ULLZ6926	KF182542	KF182484	KF182642	KF182731	KF182380
Anomura	Diogenidae	*Areopaguristes pilosus* H. Milne Edwards, 1836	NIWA:28030	HQ380271	HQ380296	HQ380283	N/A	N/A
Anomura	Diogenidae	*Calcinus laevimanus* (Randall, 1840)	KC6994/ULLZ10120	N/A	KF182471	KF182632	KF182691	KF182426
Anomura	Diogenidae	*Calcinus obscurus* Stimpson, 1859	H111	AF436058	AF436022	AF435998	FJ620465	N/A
Anomura	Diogenidae	*Clibanarius albidigitus* Nobili, 1901	N/A	AF425323	AF438751	AF425342	N/A	N/A
Anomura	Diogenidae	*Clibanarius antillensis* Stimpson, 1859	KC6973/ULLZ9433	KF182529	KF182472	KF182628	KF182693	KF182424
Anomura	Diogenidae	*Clibanarius corallinus* (H. Milne-Edwards, 1848)	KC6975/ULLZ10121	KF182528	KF182473	KF182629	KF182694	KF182423
Anomura	Diogenidae	*Clibanarius vittatus* (Bosc, 1802)	KC6855/ULLZ4781	KF182527	KF182474	KF182630	KF182692	KF182422
Anomura	Diogenidae	*Dardanus fucosus* Biffar & Provenzano, 1972	KC6858/ULLZ7122	KF182586	N/A	KF182654	N/A	KF182430
Anomura	Diogenidae	*Dardanus insignis* (de Saussure, 1858)	KC6857/ULLZ7964	KF182585	KF182498	KF182631	N/A	KF182429
Anomura	Diogenidae	*Dardanus sp.*	KC4761/ULLZ6711	KF182533	KF182468	KF182626	KF182697	KF182428
Anomura	Diogenidae	*Isocheles pilosus* (Holmes, 1900)	N/A	AF436057	AF436021	N/A	N/A	N/A
Anomura	Diogenidae	*Isocheles wurdemanni* Stimpson, 1859	KC6856/ULLZ5683	KF182530	KF182475	KF182633	N/A	KF182425
Anomura	Diogenidae	*Paguristes cadenati* Forest, 1954	KC6862/ULLZ7624	KF182540	KF182493	KF182637	N/A	KF182386
Anomura	Diogenidae	*Paguristes grayi* (Benedict, 1901)	KC6859/ULLZ11744	KF182537	KF182488	KF182636	KF182728	KF182382
Anomura	Diogenidae	*Paguristes nr. moorei*	KC6863/ULLZ11765	KF182552	KF182490	KF182640	N/A	KF182385
Anomura	Diogenidae	*Paguristes punticeps* Benedict, 1901	KC6861/ULLZ6801	KF182538	KF182487	KF182639	KF182727	KF182383
Anomura	Diogenidae	*Paguristes sericeus* A. Milne Edwards, 1880	KC4762/ULLZ7331	N/A	KF182486	KF182635	KF182726	KF182381
Anomura	Diogenidae	*Paguristes tortugae* Schmitt, 1933	KC4763/ULLZ6800	KF182534	KF182480	N/A	KF182732	KF182376
Anomura	Diogenidae	*Paguristes triangulatus* A. Milne-Edwards & Bouvier, 1893	KC6860/ULLZ6892	KF182539	KF182489	KF182638	KF182729	KF182384
Anomura	Diogenidae	*Paguristes turgidus* (Stimpson, 1857)	N/A	AF436056	AF436020	AF435997	N/A	N/A
Anomura	Diogenidae	*Petrochirus diogenes* (Linnaeus, 1758)	KC4764/ULLZ8129	N/A	KF182469	KF182627	KF182719	KF182427
Anomura	Eumunididae	*Eumunida funambulus* Gordon, 1930	KC3100	EU920922	EU920957	EU920984	EU921056	EU920892
Anomura	Eumunididae	*Eumunida picta* Smith, 1883	KC6872	KF182518	KF182463	KF182619	KF182690	KF182368
Anomura	Eumunididae	*Eumunida picta* Smith, 1883	KC6874	KF182519	KF182464	KF182620	N/A	KF182369
Anomura	Eumunididae	*Pseudomunida fragilis* Haig, 1979	KC6707	KF182517	KF182462	KF182618	KF182665	KF182370
Anomura	Galatheidae	*Alainius crosnieri* Baba, 1991	MNHN:Norfolk I Stn DW 1703	HQ380263	HQ380287	HQ380275	N/A	N/A
Anomura	Galatheidae	*Galathea rostrata* A. Milne-Edwards, 1880	KC4767/ULLZ7681	KF182523	KF182504	KF182664	KF182684	KF182388
Anomura	Galatheidae	*Galathea sp.*	KES-2008	EU821544	EU821561	EU821578	N/A	N/A
Anomura	Hapalogastridae	*Hapalogaster mertensii* Brandt, 1850	KC6175/ULLZ11535	KF182573	KF182451	KF182601	KF182667	KF182401
Anomura	Hapalogastridae	*Oedignathus inermis* (Stimpson, 1860)	N/A	AF425334	Z14062	AF425353	N/A	N/A
Anomura	Hippidae	*Emerita brasiliensis* Schmitt, 1935	KCembr	DQ079712	AF439384	DQ079786	DQ079673	N/A
Anomura	Hippidae	*Emerita emeritus* (Linnaeus 1767)	AMSP67874	AY583898	AY583971	AY583990	N/A	N/A
Anomura	Hippidae	*Emerita talpoida* (Say, 1817)	KC6850/ULLZ9434	KF182557	KF182444	KF182587	KF182702	KF182419
Anomura	Kiwaidae	*Kiwa hirsuta* Macpherson, Jones & Segonzac, 2005	MNHN:Ga 5310	EU831284	DQ219316	EU831286	EU921065	N/A
Anomura	Lithodidae	*Cryptolithodes sp.*	KC6971/ULLZ11844	KF182574	KF182453	KF182603	KF182669	KF182402
Anomura	Lithodidae	*Glyptolithodes cristatipes (Faxon, 1893)*	N/A	AF425326	N/A	AF425346	N/A	N/A
Anomura	Lithodidae	*Lithodes santolla (Molina, 1782)*	KC6340/ULLZ11875	KF182572	KF182452	KF182602	KF182671	KF182400
Anomura	Lithodidae	*Lithodes santolla (Molina, 1782)*	KAClisa	AY595927	AF439385	AY596100	DQ079679	AY595546
Anomura	Lithodidae	*Lopholithodes mandtii Brandt, 1848*	N/A	AF425333	N/A	AF425352	N/A	N/A
Anomura	Lithodidae	Paralithodes brevipes (H. Milne Edwards & Lucas, 1841)	N/A	AF425337	N/A	AF425356	N/A	N/A
Anomura	Lithodidae	Paralithodes camtschaticus Tilesius, 1815	N/A	AF425338	N/A	AF425357	N/A	N/A
Anomura	Lithodidae	*Paralithodes platypus* (Brandt, 1850)	N/A	N/A	N/A	AB193822	N/A	N/A
Anomura	Lithodidae	*Paralomis sp.*	KC3506	KF182571	KF182446	KF182588	KF182666	KF182399
Anomura	Lithodidae	*Phyllolithodes papillosus* Brandt, 1848	N/A	AF425340	N/A	AF425359	N/A	N/A
Anomura	Lomisidae	*Lomis hirta* (Lamarck, 1818)	KClohi	AF436052	AF436013	AF435993	DQ079680	AY595547
Anomura	Munididae	*Agononida procera* Ahyong & Poore, 2004	NIWA:9017	EU821540	EU821556	EU821573	N/A	N/A
Anomura	Munididae	*Anoplonida inermis* (Baba, 1994)	MNHN:SANTO Stn AT9	HQ380265	HQ380289	HQ380276	N/A	N/A
Anomura	Munididae	*Babamunida kanaloa* Schnabel, Martin & Moffitt, 2009	LACM:CR 2006-014.21	N/A	HQ380294	HQ380281	N/A	N/A
Anomura	Munididae	*Bathymunida balssi* Van Dam, 1838	MNHN:SANTO Stn AT5	HQ380266	HQ380290	HQ380277	N/A	N/A
Anomura	Munididae	*Cervimunida johni* Porter, 1903	NIWA:46109	EU821546	EU821563	EU821580	N/A	N/A
Anomura	Munididae	*Munida iris* A. Milne-Edwards, 1880	KC4768/ULLZ8366	KF182521	KF182491	KF182622	KF182685	KF182389
Anomura	Munididae	*Munida pusilla* Benedict, 1902	KC6837/ULLZ8322	KF182522	KF182492	KF182623	KF182686	KF182390
Anomura	Munididae	*Munida quadrispina* Benedict, 1902	N/A	AF436050	AF436010	AF435990	N/A	N/A
Anomura	Munididae	*Munida gregaria* (Fabricius, 1793)	KAC-mso1/Kcmusu	AY050075	AF439382	AY596099	DQ079688	AY050029
Anomura	Munididae	*Neonida grandis* Baba & de Saint Laurent, 1996	MNHN:Lifou Stn CP2	HQ380264	HQ380288	N/A	N/A	N/A
Anomura	Munididae	*Pleuroncodes monodon* (H. Milne Edwards, 1837)	NIWA:46108	EU821545	EU821562	EU821579	N/A	N/A
Anomura	Munididae	*Sadayoshia sp.*	MNHN:SANTO	EU821547	EU821564	EU821581	N/A	N/A
Anomura	Munidopsidae	*Galacantha rostrata* A. Milne-Edwards, 1880	NIWA:9002	HQ380261	EU821559	EU821576	N/A	N/A
Anomura	Munidopsidae	*Galacantha valdiviae* Balss, 1913	KC3102	EU920928	EU920961	EU920985	EU921066	EU920898
Anomura	Munidopsidae	*Leiogalathea laevirostris* (Balss, 1913)	NIWA:10197	EU821541	EU821557	EU821574	N/A	N/A
Anomura	Munidopsidae	*Munidopsis bairdii* (Smith, 1884)	NIWA:19175	EU821542	EU821558	EU821575	N/A	N/A
Anomura	Munidopsidae	*Munidopsis erinacea* (A. Milne-Edwards, 1880)	KC4769/ULLZ7810	KF182524	KF182479	KF182621	KF182689	KF182391
Anomura	Munidopsidae	*Shinkaia crosnieri* Baba & Williams, 1998	NTOU:Chan, Lee & Lee, 2000	NC_011013	N/A	EU831285	N/A	NC_011013
Anomura	Paguridae	*Agaricochirus alexandri* (A. Milne-Edwards & Bouvier, 1893)	KC4772/ULLZ6891	N/A	KF182447	KF182593	KF182672	KF182404
Anomura	Paguridae	*Bythiopagurus macrocolus* McLaughlin, 2003	NIWA:29632	EU821532	EU821548	EU821565	N/A	N/A
Anomura	Paguridae	*Discorsopagurus schmitti* (Stevens, 1925)	N/A	AF436055	AF436017	N/A	N/A	N/A
Anomura	Paguridae	*Goreopagurus piercei* Wass, 1963	KC6991/ULLZ8570	N/A	KF182456	KF182592	KF182670	KF182416
Anomura	Paguridae	*Iridopagurus caribbensis* (A. Milne-Edwards & Bouvier, 1893)	KC4774/ULLZ6759	KF182580	KF182448	KF182598	KF182687	KF182412
Anomura	Paguridae	*Iridopagurus reticulatus* García-Gómez, 1983	KC6827/ULLZ10032	KF182581	KF182449	KF182599	KF182688	KF182413
Anomura	Paguridae	*Labidochirus splendescens* (Owen, 1839)	N/A	AF425332	N/A	AF425351	N/A	N/A
Anomura	Paguridae	*Manucomplanus ungulatus* (Studer, 1883)	KC6833/ULLZ7851	KF182575	KF182457	KF182612	KF182681	N/A
Anomura	Paguridae	*Pagurus bernhardus* (Linnaeus, 1758)	N/A	AF425335	N/A	AF425354	N/A	N/A
Anomura	Paguridae	*Pagurus brevidactylus* (Stimpson, 1859)	KC4776/ULLZ7065	KF182563	KF182495	KF182610	KF182679	KF182407
Anomura	Paguridae	*Pagurus bullisi* Wass, 1963	KC6832/ULLZ11056	KF182568	KF182454	KF182595	KF182668	KF182410
Anomura	Paguridae	*Pagurus maclaughlinae* García-Gómez, 1982	KC6831/ULLZ11975	KF182566	KF182460	KF182611	KF182680	KF182408
Anomura	Paguridae	*Pagurus nr. carolinensis*	KC6830/ULLZ8576	KF182565	KF182465	KF182609	N/A	N/A
Anomura	Paguridae	*Pagurus pollicaris* Say, 1817	KC6829/ULLZ11954	N/A	KF182458	KF182589	KF182737	KF182403
Anomura	Paguridae	*Pagurus stimpsoni* (A. Milne-Edwards & Bouvier, 1893)	KC6828/ULLZ11110	KF182564	KF182466	KF182613	KF182682	KF182409
Anomura	Paguridae	*Phimochirus holthuisi* (Provenzano, 1961)	KC6834/ULLZ7973	KF182578	KF182455	KF182594	KF182678	KF182415
Anomura	Paguridae	*Phimochirus randalli* (Provenzano, 1961)	KC4777/ULLZ7071	KF182576	KF182461	KF182591	KF182676	KF182417
Anomura	Paguridae	*Phimochirus randalli* (Provenzano, 1961)	KC4778/ULLZ7345	KF182577	KF182450	KF182596	KF182677	KF182418
Anomura	Paguridae	*Porcellanopagurus filholi* de Saint Laurent & McLaughlin, 2000	NIWA:29628	HQ380267	HQ380291	HQ380278	N/A	N/A
Anomura	Paguridae	*Pylopaguridium markhami* McLaughlin & Lemaitre, 2001	KC4779/ULLZ6780	KF182570	KF182478	KF182597	KF182674	KF182414
Anomura	Paguridae	*Pylopagurus discoidalis* (A. Milne-Edwards, 1880)	KC4780/ULLZ7675	KF182569	KF182496	KF182614	KF182675	KF182405
Anomura	Paguridae	*Tomopagurus merimaculosus* (Glassell, 1937)	KC4782/ULLZ9441	KF182567	KF182497	KF182590	KF182673	KF182411
Anomura	Paguridae	*Xylopagurus cancellarius* Walton, 1950	KC4783/ULLZ9443	KF182584	KF182459	KF182600	KF182683	KF182406
Anomura	Parapaguridae	*Parapagurus latimanus* Henderson, 1888	NIWA:29621	N/A	EU821550	EU821567	N/A	N/A
Anomura	Parapaguridae	*Sympagurus acinops* Lemaitre, 1989	KC6977/ULLZ11020	KF182526	KF182476	KF182616	KF182701	KF182371
Anomura	Parapaguridae	*Sympagurus dimorphus* (Studer, 1883)	NIWA:29594	EU821533	EU821549	EU821566	N/A	N/A
Anomura	Parapaguridae	*Sympagurus pictus* Smith, 1883	KC7247/KC6835 (ULLZ 10849 16S/H3only)	KF182579	KF182477	KF182617	KF182700	KF182372
Anomura	Porcellanidae	*Allopetrolisthes spinifrons* (H. Milne Edwards, 1837)	KC6965/ULLZ5979	KF182550	KF182499	KF182662	KF182714	KF182398
Anomura	Porcellanidae	*Euceramus sp.*	KC6974/ULLZ10235	KF182555	KF182513	KF182634	KF182716	N/A
Anomura	Porcellanidae	*Megalobrachium poeyi* (Guérin-Méneville, 1855)	KC6964/ULLZ6094	N/A	KF182512	N/A	KF182713	KF182397
Anomura	Porcellanidae	*Neopisosoma angustifrons* (Benedict, 1901)	KC6968/ULLZ5385	KF182545	KF182501	KF182652	KF182712	KF182434
Anomura	Porcellanidae	*Pachycheles ackleianus* A. Milne-Edwards, 1880	KC6988/ULLZ8341	KF182554	KF182503	KF182651	KF182706	N/A
Anomura	Porcellanidae	*Pachycheles haigae* Rodrigues da Costa, 1960	KAC-pha1	AY050076	AF439389	N/A	N/A	AY050030
Anomura	Porcellanidae	*Pachycheles pilosus* (H. Milne Edwards, 1837)	KC6986/ULLZ10036	KF182544	KF182502	KF182653	KF182707	N/A
Anomura	Porcellanidae	*Pachycheles rudis* Stimpson, 1859	N/A	AF260598	AF436008	AF435988	N/A	N/A
Anomura	Porcellanidae	*Pachycheles rugimanus* A. Milne-Edwards, 1880	KC4787/ULLZ6903	KF182543	KF182500	KF182650	KF182705	KF182392
Anomura	Porcellanidae	*Parapetrolisthes tortugensis* (Glassell, 1945)	KC4788/ULLZ6726	KF182546	KF182507	KF182658	KF182709	KF182393
Anomura	Porcellanidae	*Parapetrolisthes tortugensis* (Glassell, 1945)	KC6979/ULLZ7560	KF182547	KF182508	KF182660	KF182710	KF182394
Anomura	Porcellanidae	*Petrolisthes armatus* (Gibbes, 1850)	KC6993/ULLZ10098	KF182549	KF182510	KF182661	KF182708	KF182396
Anomura	Porcellanidae	*Petrolisthes armatus* (Gibbes, 1850)	N/A	AF436049	AF436009	AF435989	N/A	N/A
Anomura	Porcellanidae	*Petrolisthes galathinus* (Bosc, 1802)	KC4789/ULLZ6897	KF182548	KF182509	KF182659	KF182711	KF182395
Anomura	Porcellanidae	*Petrolisthes laevigatus* (Guérin, 1835)	N/A	AF260606	AF439388	N/A	N/A	N/A
Anomura	Porcellanidae	*Pisidia magdalenensis* (Glassell, 1936)	KC6980/ULLZ5986	KF182556	KF182514	N/A	KF182718	N/A
Anomura	Porcellanidae	*Polyonyx gibbesii* Haig, 1956	KC6987/ULLZ12061& KC6983/ULLZ8943	KF182553	KF182515	KF182663	KF182717	N/A
Anomura	Porcellanidae	*Porcellana sayana* (Leach, 1820)	KC4790/ULLZ8092	KF182551	KF182516	N/A	KF182715	N/A
Anomura	Pylochelidae	*Pomatocheles jeffreysii* Miers, 1879	KC3097	EU920930	EU920965	EU920983	EU921070	EU920903
Anomura	Pylochelidae	*Trizocheles spinosus* (Henderson, 1888)	NIWA:29348	N/A	EU821551	EU821568	N/A	N/A
Anomura	Pylochelidae	*Xylocheles macrops* Forest, 1987	AMSP57955	AY583897	AY583970	AY583989	N/A	N/A
**Outgroup**								
Brachyura	Calappidae	*Calappa gallus* (Herbst, 1803)	KC3083	EU920916	EU920947	EU920977	EU921049	EU920886
Brachyura	Varunidae	*Cyclograpsus cinereus* (Dana, 1851)	KC3417	EU920914	EU920945	EU920997	EU921046	EU920884
Brachyura	Leucosiidae	*Praebebalia longidactyla* (Yokoya, 1933)	KC3086	EU920931	EU920946	EU920979	EU921071	EU920904
Brachyura	Epialtidae	*Chorilia longipes* (Dana, 1852)	KC3089	EU920919	EU920948	EU920981	EU921052	EU920889
Brachyura	Rainidae	*Cosmonotus grayi* (White, 1848)	KC3092	EU920918	EU920949	EU920982	EU921051	EU920888
Axiidea	Callianassidae	*Lepidophthalmus louisianensis* (Schmitt,1935)	KAC1852	DQ079717	DQ079751	DQ079792	DQ079678	EU920897
Axiidea	Callianassidae	*Sergio mericeae* (Manning & Felder, 1995)	KAC1865	DQ079733	DQ079768	DQ079811	DQ079700	EU920909
Axiidea	Axiidae	*Calaxius manningi* Kensley et al., 2000	NTOUA0053	EF585447	EF585458	EF585469	N/A	N/A
Axiidea	Calocarididae	*Calastacus crosnieri* Kensley & Chan, 1998	NTOUA00212	EF585446	EF585457	EF585468	N/A	N/A
Gebiidea	Laomediidae	*Laomedia astacina* de Haan, 1841	NTOUA00366	EF585450	EF585461	EF585472	N/A	N/A
Gebiidea	Thalassinidae	*Thalassina anomala* (Herbst, 1804)	ZRC1998-.2263	AY583896	AY583969	EF585476	N/A	N/A
Gebiidea	Upogebiidae	*Austinogebia narutensis* (Sakai, 1986)	NTOUA00416	EF585443	EF585454	EF585465	N/A	N/A
Penaeoidea	Solenoceridae	*Hymenopenaeus debilis* Smith, 1882	KC 4444/ULLZ 8531	KF182582	KF182505	KF182655	KF182721	KF182431
Penaeoidea	Solenoceridae	*Solenocera sp.*	KC 4454/ULLZ 6705	KF182583	KF182506	KF182656	KF182722	KF182432
Caridea	Hippolytidae	*Latreutes fucorum* (Fabricius, 1798)	KC 4498/ULLZ 9135	EU868664	EU868755	KF182646	KF182723	KF182435
Caridea	Atyidae	*Atyopsis sp.*	KC 4517/ULLZ 9174	EU868634	EU868724	KF182647	KF182724	KF182433
Caridea	Palaemonidae	*Palaemonetes pugio* Holthuis, 1949	KC 4523/ULLZ7458	EU868697	EU868791	KF182648	KF182725	KF182437
Caridea	Ogyrididae	*Ogyrides sp.*	KC 4542/ULLZ 7755	EU868679	EU868772	KF182649	KF182736	KF182436

An “N/A” not available indicates missing sequence data. New sequences are indicated as KFXXXXXX.

### Phylogenetic analyses

**Figure 1:**
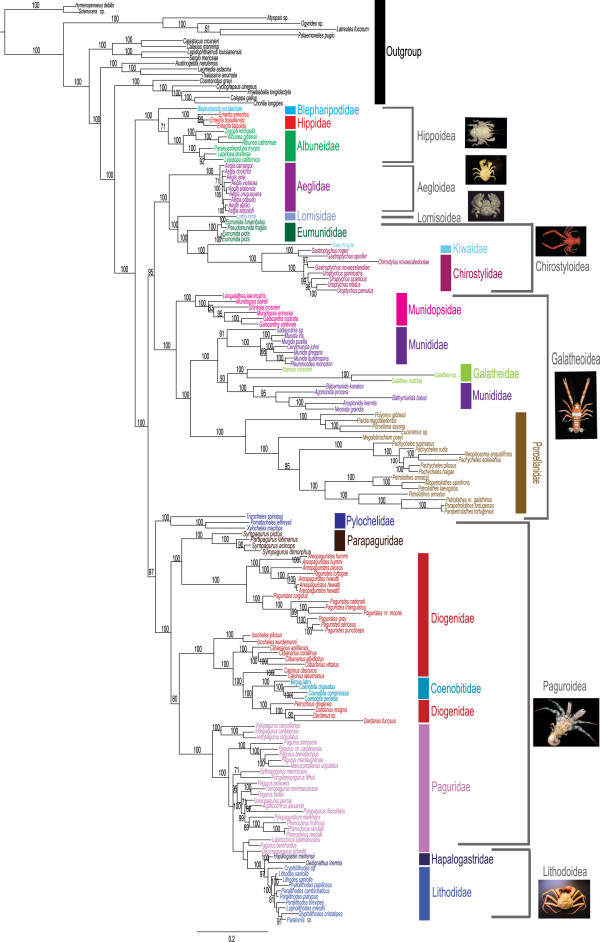
**Combined Bayesian phylogram based on molecular (3669 characters) and morphological (156 characters) data.** Vertical colored bars represent anomuran families, grey brackets represent superfamilies, and the black vertical line represents outgroups. Bayesian posterior probabilities represented as percentages and >70% are noted above or below branches.

Alternative outgroup selections did not affect internal anomuran relationships. With all outgroups included, Brachyura was recovered as the sister taxon. The Bayesian analysis from the combined molecular + morphology dataset recovers Anomura as a monophyletic group with high support (100 = Pp, Figure 1). The majority of the nodes (86%) are recovered with very high support (>95). Three families are recovered as para- or polyphyletic (Diogenidae, Paguridae, Munididae). With the exception of three families (Blepharipodidae, Kiwaidae, Lomisidae) each having a single representative, the remaining families were found to be monophyletic (Hippidae, Albuneidae, Munidopsidae, Galatheidae, Porcellanidae, Parapaguridae, Aeglidae, Eumunididae, Chirostylidae, Lithodidae, Hapalogastridae, Pylochelidae, and Coenobitidae) with high support. Blepharipodidae, Hippidae, and Albuneidae (Hippoidea) group together with very high support (100), being sister to the remaining 16 anomuran families. Lomisidae, Eumunididae, Kiwaidae, and Chirostylidae (Lomisoidea + Chirostyloidea) form a clade with high support (100) and are sister to Aeglidae (Aegloidea). Munidopsidae, Galatheidae, Munididae, and Porcellanidae (Galatheoidea) form a clade with high Bayesian support (100). Within the Galatheoidea, Munididae is paraphyletic with the galatheids nested within the group. Pylochelidae, Parapaguridae, Diogenidae, Coenobitidae, Paguridae, Hapalogastridae, and Lithodidae (= Paguroidea + Lithodoidea) form a statistically supported clade (97). Six of the seven anomuran superfamilies are monophyletic (Hippoidea, Galatheoidea, Aegloidea, Lomisoidea [monotypic], Chirostyloidea, and Lithodoidea). The remaining superfamily, Paguroidea is found to be paraphyletic and includes the superfamily Lithodoidea (Lithodidae + Hapalogastridae). 11 genera were found to be poly- or paraphyletic (*Eumunida, Gastroptychus, Munidopsis, Munida, Pachycheles, Petrolisthes, Sympagurus, Areopaguristes, Paguristes, Pagurus,* and *Paralithodes*).

**Figure 2:**
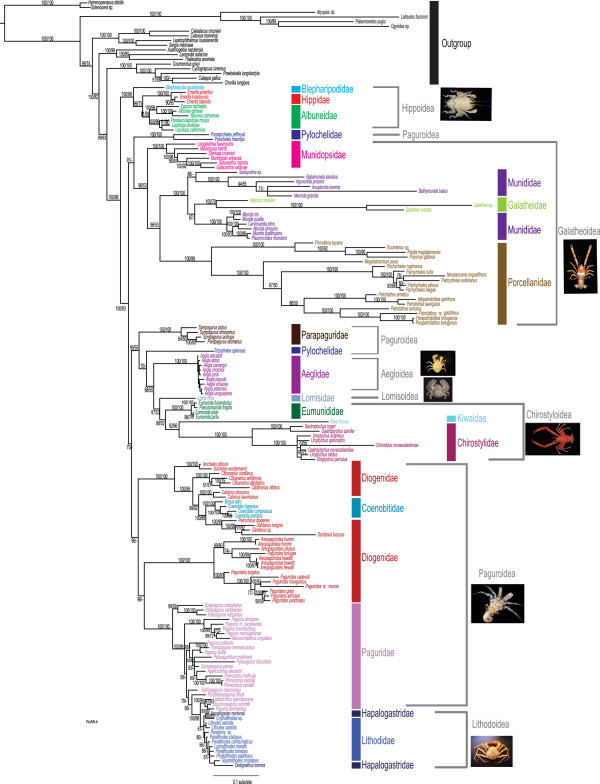
**Bayesian phylogram based on 5 genes 12S, 16S, 18S, 28S, H3 and 3669 characters.** Vertical colored bars represent anomuran families, grey brackets represent superfamilies, and the black vertical line represents outgroups. Bayesian posterior probabilities represented as percentages and maximum likelihood bootstrap values are noted above or below branches.

The molecular-only phylogeny (Figure 2) is similar to our combined phylogeny, with most differences being found in placement and composition of Paguroidea. Unlike the combined phylogeny, which recovered Paguroidea as paraphyletic, Paguroidea was found to be polyphyletic. The family Pylochelidae was recovered as polyphyletic according to molecular data but was monophyletic when morphology was added. Parapaguridae was sister to a clade containing Pylochelidae, Aeglidae, Lomisidae, Eumunididae, Kiwaidae, and Chirostylidae, similar to the results of Tsang et al. [20] based on nuclear protein coding genes. As in the combined phylogeny, Coenobitidae is nested within the Diogenidae, and Lithodoidea nested within the Paguroidea. Within Lithodoidea of the molecular–only phylogeny, Hapalogastridae was found to be paraphyletic, with representatives of the genera *Hapalogaster* and *Oedignathus* at the basal (*H. mertensi*) and derived (*O. inermis*) end of the tree. However lithodoid relationships in the molecular-only phylogeny should be interpreted with caution as many were recovered with little to no support. In the combined phylogeny Hapalogastridae was found to be a monophyletic and sister to Lithodidae (Figure 1). The remaining superfamilies were monophyletic as recovered in the combined tree (Figures 1 and 2). Twelve genera were found to be poly- or paraphyletic (*Munidopsis, Munida, Pachycheles, Petrolisthes, Sympagurus, Eumunida, Gastroptychus, Uroptychus, Paguristes, Areopaguristes, Pagurus,* and *Paralithodes*)*.* Some deep splits and short branches in the molecular-only phylogeny should be interpreted with caution, as support is low.

### Alternative hypotheses

Alternative hypotheses regarding monophyly of the families Paguridae, Diogenidae, Hapalogastridae, Lithodidae, Munididae, Pylochelidae, and the superfamily Paguroidea [14-18,23,39] were tested using the Shimodaira-Hasegawa test (S-H). Three of the seven hypotheses were found to be significantly worse than our unconstrained topology (*P* < 0.05; ML_best_ = −68420.363272; ML_Diogenidae_ = −68667.853268, ML_Paguridae_ = −68497.123254; ML_Paguroidea_ = −68825.722919). The remaining four hypotheses were not found to be significantly worse than our unconstrained topology (*P* > 0.05; ML_Hapalogastridae_ = −68432.438825; ML_Lithodidae_ = −68438.309801; ML_Munididae_ = −68428.284597; ML_Pylochelidae_ = −68430.951016). Hypotheses that tested a “king to hermit” evolutionary pathway were all significantly worse than the alternative (i.e., “hermit to king”) as recovered in our best ML tree (*P* < 0.05; ML_best_ = −68420.363272; ML_king-Paguroidea_ = −68777.179402; ML_king-Paguridae_ = −68713.171227).

### Character evolution

**Figure 3:**
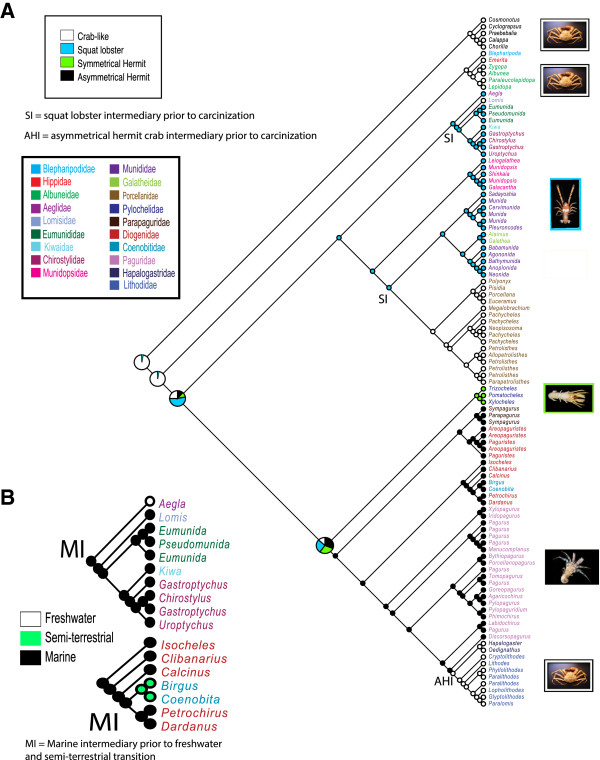
**Ancestral state reconstruction analysis using maximum likelihood methods for body shape and habitat transition within Anomura.** Colored taxa correspond to anomuran families as noted in legend. Pie charts represent the likelihood of the ancestral state. (**A**) Character states for body shape were defined as crab-like white, squat lobster blue, symmetrical hermit green and asymmetrical hermit black. (**B**) Character states for habitat were defined as freshwater white, semi-terrestrial green, and marine black. Subtrees are shown for the transition into freshwater (Aeglidae) and semi-terrestrial habitats (Coenobitidae).

To infer evolutionary pathways, body forms (crab-like, squat lobster, asymmetrical hermit pleon, symmetrical hermit pleon) and habitat types (marine, freshwater, semi-terrestrial) were optimized across our combined phylogeny using ancestral state reconstruction methods (Figure 3A). Analyses indicated that a crab-like ancestor gave rise to all extant anomuran lineages. In addition to the earliest branching clade, Hippoidea, carcinization occurred independently three times during the evolution of the group, twice through squat lobster-like intermediaries (squat intermediary = SI on tree) and once through an asymmetrical hermit crab-like ancestor (asymmetrical hermit intermediary = AHI on tree) (Figure 3A). The squat lobster-like form arose once as an early branching lineage and gave rise to the crab-like clades, Lomisidae and Porcellanidae. Within the hermit crab lineages, the symmetrical pleon arose once within the Pylochelidae. The asymmetrical pleon arose once within the Paguroidea, but was subsequently partially reverted to the ancestral symmetrical condition (in males only) within the crab-like Lithodidae and Hapalogastridae (= Lithodoidea, Figure 3A). We traced the colonization of freshwater and semi-terrestrial habitats by the families Aeglidae and Coenobitidae (Figure 3B). Both transitions occurred via marine ancestors (marine intermediary = MI in Figure 3B). In combination with divergence time results, we can make predictions about the timing of these events (see Discussion). Maximum parsimony and maximum likelihood methods recovered similar ancestral state reconstructions for body form and habitat (Figure 3A and B), so only the likelihood analyses are presented.

### Divergence time analysis

**Figure 4:**
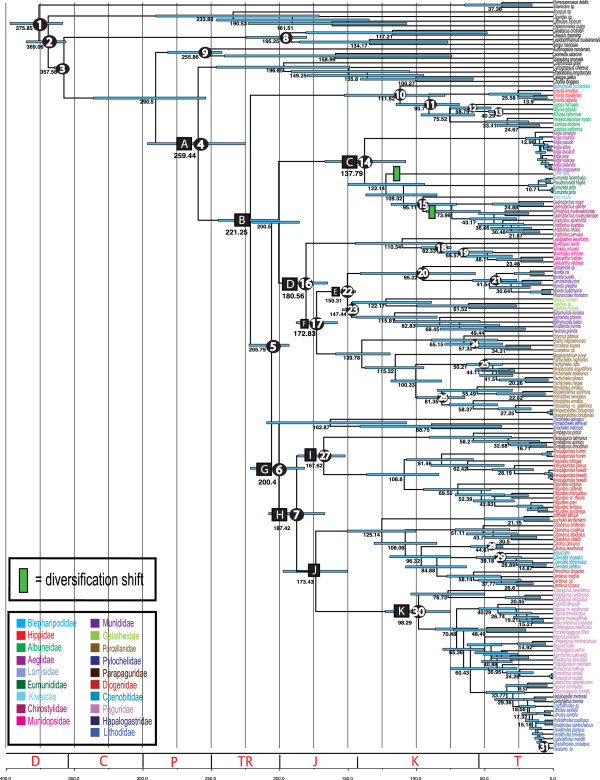
**Divergence time chronogram using Bayesian evolutionary analysis by sampling trees using BEAST.** Fossil calibration points are indicated by numbers 1–31 embedded in black circles (refer to Table 2). Divergence time estimates (MY) are noted adjacent to their respective nodes and blue nodal bars correspond to the 95% highest posterior density regions. Geological periods are superimposed onto the phylogeny and listed as follows: D, Devonian; C, Carboniferous; P, Permian; TR, Triassic; J, Jurassic; K, Cretaceous; T, Tertiary. Colored taxa correspond to anomuran families as noted in the legend. Green boxes indicate a diversification shift.

The divergence dating program BEAST was used to estimated origins and radiations of major lineages based on 31 fossil calibrations (Table 2). All parameters reached convergence for individual runs. BEAST estimated the divergence of the anomurans from the true crabs, Brachyura, to be in the Permian (~259 (224–296) MYA, Figure 4, Square A). The most recent common ancestor of all present-day families radiated shortly afterwards in the Triassic representing the origin of the earliest branching clade (Blepharipodidae-Albuneidae-Hippidae) estimated in the Norian (~221 MYA, Square B). Additional speciation events leading to these present-day families occurred throughout the Cretaceous (~111-90.7 MYA). The exclusively freshwater family Aeglidae diverged in the Early Cretaceous (~137 MYA, Square C) with rapid speciation of present day species occurring since the mid-Miocene (~12 MYA). The families Lomisidae, Eumunididae, Chirostylidae, and Kiwaidae all originated in the Cretaceous (~122, 109, 95, and 95 MYA respectively). Squat lobsters and porcelain crabs within the superfamily Galatheoidea originated in the Early Jurassic (Hettangian) and split into the Munidopsidae and remaining families during the Pliensbachian, Early Jurassic (~180 MYA, Square D). The other galatheoid families, Munididae and Galatheidae, arose soon thereafter within the Tithonian, Late Jurassic (~150 MYA, Square E) while Porcellanidae emerged in the Aalenian, Middle Jurassic (~173 MYA, Square F). The oldest family of hermit crabs, the symmetrical pylochelids, branched from the remaining hermits around 200 MYA in the Norian, Late Triassic (Figure 4, Square G). The origin of the asymmetrical hermit crab lineages followed soon after in the Pliensbachian, Early Jurassic (~187 MYA, Square H). Two hermit crab families were recovered as non-monophyletic assemblages (Diogenidae, Paguridae), which resulted in multiple timing of origins for these families. Parapaguridae split from one clade of Diogenidae (*Areopaguristes* and *Paguristes*) in the Bathonian, Middle Jurassic (~167 MYA, Square I), while the family Coenobitidae is found nested within a slightly older clade of Diogenidae (~173 MYA, Square J), which includes most present day genera. Paguridae is not monophyletic, because of the internally nested Lithodidae and Haplogastridae. The most recent common ancestor of the pagurid + lithodid + hapalogastrid clade was placed in the Late Cretaceous (Cenomanian, ~98 MYA, Square K) with Lithodidae and Hapalogastridae splitting from one another around 18 MYA (Burdigalian, Miocene).

**Table 2:** Fossil calibrations used in BEAST divergence time analyses

**Taxonomy**	**Species**	**Geological Age (MYA)**	**Node**
**Outgroup**			
**Natantia**			
**Suborder Dendrobranchiata**			
Superfamily *Penaeoidea*	*Aciculopoda mapesi* Feldmann and Schweitzer, 2010	Late Devonian (Fammenian) 359-374	1
**Suborder Pleocyemata**			
Infraorder *caridea*	*Pinnacaris dentata* Garassino and Teruzzi, 1993	Late Triassic (Norian) 204-228	2
**Reptantia**	*Palaeopalaemon newberryi* Whitfield, 1880	Late Devonian 354-370	3
Infraorder *axiidea*	*Callianassa s.l. bonjouri* Étallon, 1861	Early Jurassic (Toarcian) 176-183	8
Infraorder *gebiidea*	*Upogebia s. l. obscura* von Meyer, 1834	Early Triassic 245-251	9
**Ingroup**			
Infraorder *anomura*	*Platykotta akaina Chablais,* Feldmann and Schweitzer, 2011	Late Triassic (Norian/Rhaetian) 201.6-228	4
**Superfamily Aegloidea**			
Family *Aeglidae*	*Protaegla miniscula* Feldmann, Vega, Applegate, and Bishop, 1998	Early Cretaceous (Albian) 99.6-112	14
**Superfamily Chirostyloidea**	*Pristinaspina gelasina* Schweitzer and Feldmann, 2001	Late Cretaceous 65.5-99.6	15
**Superfamily Galatheoidea**			
Family *Galatheidae*	*Galatheites zitteli* (Moericke, 1889)	Late Jurassic (Tithonian) 145.5-151	23
Genus *Shinkaia*	*Shinkaia katapsyxis* Schweitzer and Feldmann, 2008	Eocene 33.9-55.8	19
Family *Munididae*	*Juracrista perculta* Robins, Feldmann, and Schweitzer, 2012	Late Jurassic (Tithonian) 145.5-151	22
Genus *Munida*	*Munida konara* Schweitzer and Feldmann, 2000	Oligocene-Miocene 5.3-36.6	21
Genus *Sadayoshia*	*Sadayoshia pentacantha* (Muller and Collins, 1991)	Late Eocene (Priabonian) 33.9-37.2	20
Family *Munidopsidae*	*Based upon a drawing and description only, type material apparently destroyed: Palaeomunidopsis moutieri* Van Straelen, 1925	Middle Jurassic (Bathonian) 168-165	16
	Based upon actual fossil material: *Gastrosacus wetzleri* Von Meyer, 1851	Late Jurassic (Oxfordian-Tithonian) 161-145	
Genus *Munidopsis*	*Munidopsis foersteri* Feldmann et al., 1993	Late Cretaceous (Campanian) 70.6-83.5	18
Family *Porcellanidae*	*Jurellana tithonia* Schweitzer and Feldmann, 2010	Late Jurassic (Tithonian) 145.5-151	17
Genus *Pachycheles*	*Pachycheles dorsosulcatus* Beschin, Busulini, De Angeli, and Tessier, 2007	Eocene 36.6-57.8	25
Genus *Petrolisthes*	*Petrolisthes bittneri* De Angeli and Garassino, 2002	Oligocene 23.7-36.6	26
Genus *Pisidia*	*Pisidia dorsosinuata* De Angeli and Garassino, 2002	Eocene 36.6-57.8	24
**Superfamily Hippoidea**			
Family *Albuneidae*	*Praealbunea rickorum* Fraaije, 2002	Late Cretaceous (Maastrichtian) 65.5-70.6	11
Genus *Albunea*	*Albunea cuisiana* Beschin and De Angeli, 1984	Eocene 33.9-55.8	13
Genus *Zygopa*	*Zygopa galantensis* De Angeli and Marangon, 2001	Oligocene 23-33.9	12
Family *Blepharipodidae*	*Lophomastix antiqua* Schweitzer and Boyko, 2000	Eocene 33.9-55.8	10
**Superfamily Lithodoidea**			
Family *Lithodidae*	*Paralomis debodeorum* Feldmann, 1998	Miocene 5.3-23	31
**Superfamily Paguroidea**	Based upon claws only: *Palaeopagurus deslongchampsi* Van Straelen, 1925	Early Jurassic (Pliensbachian) 190-183	5
	*Based upon carapace material: Diogenicheles theodorae* Fraaije et al., 2012	Late Jurassic (Oxfordian) 161-156	
Family *Coenobitidae*	*Birgus latro Linnaeus, 1767*	Pliocene 2.6-5.3	29
Family *Diogenidae*	*Annuntidiogenes ruizdegaonai* Fraaije et al., 2008	Early Cretaceous (Albian) 99.6-112	7
Genus *Calcinus*	*Calcinus agnoensis* Beschin et al., 2005	Eocene 33.9-55.8	28
Family *Paguridae*	*Pagurus malloryi* Schweitzer and Feldmann 2001	Oligocene 23.7-36.6	30
Family *Parapaguridae*	*Eotylaspis wehnerae* van Bakel et al., 2008	Late Jurassic (Kimmeridgian) 151-156	27
Family *Pylochelidae*	*Jurapylocheles malutka, Ammopylocheles mclaughlinae* Van Bakel et al. 2008	Late Jurassic (Kimmeridgian) 151-156	6

Number corresponds to nodal placement as assigned in Figure 4.

### Diversification analyses

The reworked version of MEDUSA [40] was used to detect whether any clade within the anomuran tree was best explained by independent diversification models, and to specifically address whether acquisition of the crab-like form resulted in an increase of diversification rates. The background tempo of diversification across the anomuran tree is characterized by a speciation rate *lambda* of 0.032572 lineages/Myr, and our results suggest that the diversification of anomurans is characterized by two periods where the tempo of diversification changes (Figure 4). A slow speciation rate is detected in the lineage leading to the monotypic and carcinized family Lomisidae, and an increase rate occurred in the squat-lobster family Chirostylidae. The ancient but species-depauperate branch leading to the monotypic family Lomisidae was optimally modelled separately with maximum likelihood estimate of *lambda* = 0 (rate reduction). The rate shift that occurred in the branch leading to the family Chirostylidae was characterized by a speciation rate *lambda* of 0.054182 (rate acceleration). All three resulting clade-specific diversification models were optimally fit as Yule models (AIC = 339.3032).

## Discussion

### Phylogenetic relationships

Recent studies on anomuran evolution have used molecular data [20,21,25,38], morphological (including developmental) data [41-43], or a combination of the two [19] to resolve phylogenetic relationships. These studies have dramatically increased our understanding of anomuran relationships and resulted in several major changes within higher-level classification [17,18,27]. The instability of anomuran taxonomy in recent years highlights the need for continued phylogenetic study of this group at many levels, and we for now elect to follow the most recent and up-to-date classification scheme [17,18,22,44-46].

Our total evidence approach combines 3669 molecular (nuclear and mitochondrial) and 156 morphological (adult, sperm and larval) characters from 137 species to recover the anomuran phylogeny (Figure 1). The addition of morphological data increased the support for many intra-familial and superfamily relationships that were poorly supported in the molecular-only phylogeny (Figure 2). As mentioned previously, anomurans have undergone dramatic changes in higher-level classification based on recent phylogenetic studies. Galatheoidea has been revised recently to exclude Aeglidae, Kiwaidae, and Chirostylidae [18,23], and include only Galatheidae, Munididae, Munidopsidae, and Porcellanidae [18]. With the recent revision of Galatheoidea, all superfamilies were recovered as monophyletic (i.e., Hippoidea, Aegloidea, Lomisoidea, Chirostyloidea, Galatheoidea, Lithodoidea), except for Paguroidea (Figures 1 and 2). We found Lithodoidea to be nested within Paguroidea, which is in accordance with all recent combined (molecular + morphology) and molecular-based phylogenetic studies [19-21,33,41]. An affinity between certain Lithodidae (*Lithodes*) and Paguridae (*Pagurus*) has been suggested since the early 1800’s [see [32] for review of literature], based on morphological characters including mouthparts, gills, and pleonal characters. However, the evolutionary pathways of the two groups continue to be debated (see also “Hermit to King, King to Hermit Evolutionary Hypotheses”) with all recent evidence pointing to a “hermit to king” hypothesis.

Family-level relationships were well resolved in the combined analysis (Figure 1) and in accordance with recent changes in classification [17,19]. In 2010, *Eumunida* and *Pseudomunida* were removed from Chirostylidae and included in the newly erected Eumunididae*,* and the new family Munididae was erected on the basis of morphological and molecular evidence [17,18]. Our results generally support these taxonomic revisions, recovering the Eumunididae as a monophyletic group, but finding Munididae to be possibly paraphyletic (Figure 1). Galatheidae was found nested inside Munididae, but alternative topologies that recovered Munididae as monophyletic were not significantly worse than our best estimate (see Results). Deeper sampling within both families is needed to resolve family and genus level relationships. The families Galatheidae, Munidopsidae, and Porcellanidae were all recovered as monophyletic with high support (Figure 1). The paraphyly and/or polyphyly of Diogenidae and Paguridae is consistent across the combined and molecular phylogenies and in accordance with recent phylogenies that have sampled sufficiently within these families [19-21,25,26,41]. Alternative hypotheses proposing the monophyly of these families (i.e., Diogenidae, Paguridae) were rejected using S-H tests, confirming our findings (see Results). Coenobitidae (semi-terrestrial hermit crab) was deeply nested within Diogenidae (left-handed hermit crabs) while *Paguristes* and *Areopaguristes* are more closely related to Parapaguridae (deep-water hermit crabs) than to other members of Diogenidae (Figure 1). This relationship was first proposed by Boas [47], which he collectively called the Paguristinen. The families Pylochelidae and Hapalogastridae were found to be polyphyletic in the molecular analysis (Figure 2), but monophyletic in the combined analysis (Figure 1). Although we did not find Pylochelidae to be polyphyletic in our combined tree, alternative molecular-based and morphological phylogenies have recovered similar results that suggest a polyphyletic Pylochelidae [20,48]. Additionally, there is morphological support for polyphyly among pylochelids separating *Trizocheles* and *Mixtopagurus* from the remaining pylochelid genera (based on form of ocular acicles, eye type and larval forms [20,48]).

Generic relationships within Anomura seem to be much less resolved than superfamily and family level relationships. We found several genera to be poly- or paraphyletic (i.e., *Munida, Munidopsis, Paguristes)*, in agreement with previous studies [19,20,41,49]. Most instances of non-monophyly occur within highly speciose genera (i.e., *Paguristes* = ~115 spp., *Pagurus* = ~170 spp., *Munida* = ~240 spp*.)*, suggesting deeper sampling and continued research needs to be undertaken on these groups.

### King to hermit and hermit to king evolution: historical to recent hypotheses

Although past studies have shown an affinity between Paguridae (hermit crabs) and Lithodidae (king crabs), the evolutionary pathways and ancestry of these anomuran lineages have been debated for the past two centuries. The traditional and prevalent hypothesis posits that lithodids are free-living hermit crabs that abandoned shell use and underwent a series of morphological changes (carcinization) resulting in a crab-like form. It has been argued that the asymmetry of the lithodid female pleon, in particular, is evidence of asymmetrical hermit crab ancestry. Boas [31,50] was the first to suggest the evolution of lithodids specifically from pagurid ancestors, and based on morphology proposed the ancestral pagurid to be closely related to *Nematopagurus* and *Pylopagurus*. Bouvier [51-53] similarly derived the lithodids from the pagurids, agreeing with Boas on the structural pleonal similarities between these two groups. However, Bouvier also proposed a series of gradual and linear progressive stages in the transformation of the pagurid pleon, starting from a pagurid precursor to various genera of hapalogastrids (*Hapalogaster*, *Dermaturus*) and lithodids (from *Neolithodes*, *Paralithodes*, *Lithodes*, *Lopholithodes, Paralomis, Rhinolithodes,* to *Cryptolithodes*). In modern times, this concept of pagurid and lithodid evolution was brought to attention when Cunningham et al. [54] coined the phrase “from hermit to king” in applying molecular analysis to study hermit crab and lithodid phylogeny, and was then widely popularized [55]. A subsequent morphologically-based phylogenetic study by Richter and Scholtz [56] supported this same evolutionary view of pagurid and lithodid evolution. Recently, a study that examines the hemolymph vascular system in hermit and king crabs found close similarities in arterial systems of the dorsal cephalothorax [57].

An alternate, opposite view, often stated as the “king to hermit” evolutionary hypothesis, was proposed by McLaughlin and Lemaitre [32]. Using morphological characters and an unusual application of cladistic methodology, McLaughlin and Lemaitre explored possible evolutionary pathways of carcinization across Anomura (rather than attempt to determine precise phylogenetic relationships among taxa). They acknowledged that the crab-like form might have arisen multiple times across the Anomura, but in the case of pagurid/lithodid evolution they concluded that the opposite evolutionary trajectory was more plausible, i.e., the transition was from a “crab-like” body form to a ‘hermit-crab” body form through a series of habitat change, calcium loss, and consequential adult morphological adaptations. Subsequent studies showed that the linear evolutionary scenario proposed by Bouvier did not correspond to the ontogenetic changes that take place in the megalopa to juvenile crab stages in at least 10 species of eight lithodid genera [43,58]. Based on observations of the complex changes in pleonal tergites from megalopa to juvenile crab stages, these studies demonstrated that adult lithodid pleonal tergite structure in several species was the result of decalcification and sundering, not secondary calcification and fusion as had been proposed by Bouvier.

Our recent phylogenetic reconstruction of anomurans based on molecular and morphological data supports the traditional “hermit to king” hypothesis in congruence with all recent studies [19-21,33,59]. With the largest number of taxa and most robust molecular/morphological dataset ever used in a phylogenetic study of anomurans, our study once again shows Lithodoidea to be nested within Paguridae. Moreover, our conclusions are consistent with the fossil record, which suggest hermits are much older (Jurassic) than king crabs (Miocene, Table 2). Finally, topology testing rejects the “king to hermit” hypothesis and finds it as significantly worse than the alternative (*P* < 0.05) (i.e., “hermit to king”) (see Results).

While there is undeniable evidence of a close relationship between hermits and king crabs, it is less clear how morphological changes associated with carcinization may have proceeded within the Lithodoidea. A recent study comparing hermit and king crab circulatory systems identified several vascular changes that occurred as the result of carcinization, arguing for more comparative studies that look at morphology (both internal and external) and development [57]. However, only with a clear phylogenetic hypothesis can many of these studies be correctly interpreted. Recent molecular or combined morphological-molecular phylogenies recover conflicting evolutionary relationships, although only three lithodoid genera (and not always the same, or excluding Hapalogastridae) have been used in previous analyses [19-21,33]. Our phylogenetic reconstruction (Figure 3) shows the less carcinized and less calcified Hapalogastridae as sister to Lithodidae, in agreement with virtually every study since Bouvier’s in the 19th century. But within Lithodidae, and in contrast to Bouvier’s linear hypothesis, our study places *Cryptolithodes*, the most heavily calcified and carcinized lithodid, as an early branching lineage followed by more derived genera (see also McLaughlin and Lemaitre, 1997, Figure 2). It thus appears that the process of heavy calcification may have appeared at least twice in lithodid lineages. More lithodoid genera/species are needed to examine the process of carcinization within the Lithodoidea and to properly test Bouvier’s and Boas’ earlier hypotheses (explaining the transition of a shell-dwelling hermit crab to a fully calcified lithodid crab). In conclusion, while recent, modern studies, including ours, overwhelmingly and clearly support a “hermit to king” evolutionary scenario, it is also clear that the evolutionary process and concomitant morphological changes (particularly in pleonal tergal plates and pleopods) that occurred within the Lithodoidea to produce the various degrees of crab-like forms in that family, is at best poorly understood.

In our reaffirmation here of the “hermit to king” hypothesis, we revealed a close relationship between Lithodoidea and the pagurid, *Discorsopagurus* (Figures 1, 2, 3 and 4). Curiously, the same close relationship has surfaced in previous studies [21,33]. This revelation is important to highlight because the “hermit [Paguridae] to king [Lithodidae]” hypothesis presupposes a distinctly asymmetrical shell-dwelling hermit crab-like ancestor from or close to the Paguridae, or more precisely *Pagurus*, as proposed by early [52] as well as modern studies [54]. However, *Pagurus* is currently a taxonomic and paraphyletic conundrum of more than 160 species, and it remains unknown which of the different lineages within “*Pagurus*” could be the most likely candidate for lithodoid ancestry. The close relationship between *Discorsopagurus* and Lithodoidea may suggest a *Discorsopagurus*-like hermit crab as the precursor to the crab-like lithodoids. All species of *Discorsopagurus* are tube-dwellers, not shell-dwellers, and show pleonal asymmetry only in having unpaired pleopods. The genus is relatively small in size compared to the typically large-sized lithodoids with a distribution across both sides of the North Pacific, from the Sea of Japan to Puget Sound and the Straits of Juan de Fuca, Washington [60]. The relationship between *Discorsopagurus* and lithodoids may not be coincidental in the North Pacific region where *D. schmitti* (this analysis) and all other *Discorsopagurus* species are found [61-63]. This region harbors the highest diversity of lithodoids, so it is plausible to expect closely related species (*Discorsopagurus*) in similar areas. Future studies with increased sampling within these groups will shed light into the evolutionary pathway of lithodoids from paguroid (possibly *Discorsopagurus*-like) ancestors.

### Divergence times and character evolution

Our divergence dating analysis estimated the origin of Anomura to be in the Late Permian (~259 MYA) from a symmetrical crab-like ancestor (Figures 3 and 4). This is consistent with many higher-level decapod phylogenies finding Anomura and Brachyura as sister clades [26,35,37,56], including the present study (Figure 1). Results estimate that the earliest diverging anomurans are the hippoids (~221 MYA), consistent with recent molecular estimates [20]. Although this date is considerably older than the hippoid fossil record, closely related extinct forms extend into the Triassic and present day Hippoidea are found in substrates underrepresented in the fossil record. The superfamily Hippoidea containing Blepharipodidae, Hippidae, and Albuneidae, has been described as being similar to primitive brachyurans [20,64], and ancestral reconstruction analysis confirms that the present day hippoids were derived from crab-like (brachyuran-like) predecessors (Figure 3A). The next radiation occurred in the Late Triassic, giving rise to the squat-lobsters and crab-like superfamilies Chirostyloidea and Galatheoidea, Aegloidea, Lomisoidea, and the hermit crab and crab-like superfamilies Paguroidea and Lithodoidea. Our results suggest these superfamily clades were derived from a squat-lobster-like ancestor approximately ~205 MYA (Figures 3A and 4). Interestingly, our divergence time and character reconstruction analyses (Figures 3A and 4) are consistent with fossil evidence, and more specifically, the discovery of *Platykotta akaina*, the oldest known anomuran fossil [1]. *Platykotta akaina,* with a possibly squat-lobster-like body form, dates back to the Late Triassic (~201.6-228 MYA) and has strong morphological affinity with the superfamilies Chirostyloidea and Galatheoidea. This fossil was found as part of a biotic assemblage suggesting that *Platykotta akaina* thrived in tropical-subtropical waters and lived in the subtidal with connections to the open ocean [1,65].

Around 137 MYA a squat-lobster like ancestor gave rise to a unique superfamily of anomurans, Aegloidea. Aegloid crabs represent the only freshwater anomuran family and can be found in caves, lakes, and streams throughout the Neotropical region of South America [66]. Apart from a single species of freshwater hermit crab, *Clibanarius fonticola*[67], the transition into a completely freshwater environment only occurred in extant Aeglidae (Figure 3B). Fossil evidence suggests freshwater aeglids once inhabited marine waters, based on the fossil representative, *Haumuriaegla glawssneri*, found in New Zealand from Late Cretaceous rocks [68]. In combination with our divergence time analyses, we hypothesize that the complete transition in freshwater occurred sometime between the Late Cretaceous and Miocene. This transition appears to have allowed for rapid diversification approximately 13 MYA (20–7.4 MYA).

From approximately 180 MYA to 147 MYA, the families of Galatheoidea radiated and diversified. These include the squat lobsters families Munidopsidae, Munididae and Galatheidae, and the porcelain crab family Porcellanidae. The porcellanids diverged in the Middle Jurassic (~172 MYA) from squat-lobster like ancestors, but a crab-like body form evolved by the Tithonian (~151-145.5 MYA) based on fossil evidence and ancestral reconstruction analyses. This was the first occurrence of carcinization from a squat-lobster or hermit-like ancestor within Anomura (Figures 3A and 4). Interestingly, Henderson [69] and Ortmann [70] suggested porcellanid crabs were derived galatheids despite the differences in body shape and form, and this is consistent with our current evolutionary hypothesis.

Lomisoidea and Chirostyloidea diverged around 122 MYA from a squat-lobster like ancestor. This body form was retained within the chirostyloids and underwent further carcinization, attaining a crab-like form in the monotypic Lomisidae, endemic to Australia.

Early hypotheses based on larval evidence proposed hermit crabs evolved as two independent lineages, Coenobitoidea and Paguroidea [71] and recent studies continue to explore superfamily and family level relationships [20,23,39]. In our combined analysis, the hermit crab families, Pylochelidae, Parapaguridae, Diogenidae, Coenobitidae, and Paguridae, formed a monophyletic group with the inclusion of Lithodidae or king crabs, and Hapalogastridae. We estimated these families arose early in the evolution of Anomura, approximately 205 MYA. The symmetrical hermit crabs, Pylochelidae, are unique with most having complete body symmetry and in utilizing broken gastropod shells, siboglinid tubes, and coral pieces for shelter and protection, in contrast to other hermit groups that commonly use coiled gastropod shells [42]. Our analysis suggests pylochelids branched early in the evolution of hermit and king crabs, consistent with morphological and fossil evidence that place them as a basal primitive lineage [39]. The oldest hermit crab fossils, *Jurapylocheles malutka, Ammopylocheles mclaughlinae* and *Eotylaspis wehnerae*[72] of Kimmeridgian age (~151-156 MYA), belong to the families Pylochelidae and Parapaguridae (Table 2). This is consistent with our divergence time analysis, which recovers these families as early branching lineages. Diogenidae, Coenobitidae, and Paguridae typically possess an asymmetrical pleon accompanied by an enlarged right or left chela. According to our combined analysis, pleonal asymmetry in hermits appears to have been derived once in the evolution of the anomurans, most probably between 200–187 MYA. This contrasts with the results obtained by Tsang et al. [20], who proposed that the pleonal asymmetry evolved independently in two different hermit crab lineages, once in Parapaguridae, and a second time in Diogenidae, Coenobitidae, and Paguridae. These contrasting differences are the result of incongruent phylogenies based on total evidence (molecular + morphology, this paper) and molecular only approaches [20]. Note, however, that our molecular-only analyses recover similar results to those of Tsang et al. [20]. The semiterrestrial coenobitids colonized land from a marine ancestor sometime between 84 and 39 MYA (Figures 3B and 4). The emergence of Diogenidae (~173-167 MYA), Coenobitidae (~84 MYA), and Paguridae (~173 MYA) all predate their first appearance in the fossil record (Table 2, Figure 4). Carcinization occurred for the third time in the crab-like superfamily Lithodoidea between 29–18 MYA from an asymmetrical hermit-like ancestor. This estimation is consistent with other timing estimates of king crab carcinization [54].

### Carcinization

The crab-like body form was recovered in our study as the ancestral state for all the anomurans. In our study, all alternative body forms were present (crab-like, squat lobster, symmetrical hermit, and asymmetrical hermit) early in the divergence of the anomurans. From these ancestral character states, carcinization occurred independently three times during the evolution of Anomura, once in the Lithodoidea through an asymmetrical hermit intermediate, and twice in Lomisidae and Porcellanidae through squat lobster intermediates (see AHI and SI, Figure 3A). These evolutionary pathways of the crab-like form, twice from squat lobster intermediaries and once through an asymmetrical ancestor, corroborates recent hypotheses [20]. However, our tree differs significantly from Tsang et al.’s study [17] in the deep ancestral origins of carcinization. Tsang et al.’s hypothesis suggests a symmetrical hermit crab-like ancestor predated the squat lobster and asymmetrical intermediaries, whereas we recovered a crab-like ancestor to predate these intermediaries. We acknowledge that our analysis recovers two deep nodes that are unresolved, however symmetrical reconstruction at these nodes seems unlikely (Figure 3A). It must also be noted that the most recent common ancestor of Anomura is unresolved in the Tsang et al. analysis, although it appears to be a crab-like or symmetrical hermit ancestor. The major differences in the two analyses stems from the differences in phylogeny and more specifically the monophyly (our study) or polyphyly [17] of Paguroidea and families therein (i.e., Pylochelidae). There is agreement with Tsang et al. in the sister group relationship between Paguridae and Lithodoidea, although Tsang et al. used only four lithodid genera (vs. eight in our study) and did not include representatives of Hapalogastridae. In addition, both studies provide strong evidence for the intermediary ancestors directly predating carcinization across Anomura (twice through squat lobster (SI) and once through asymmetrical hermit (ASI), Figure 3A).

The multiple cases of carcinization among the anomurans have been noted since the early 1900s. Borradaile (1916) was the first to propose the term carcinization to explain the crab-like aspects of the hermit crab *Porcellanopagurus* and the tendency of anomurans to achieve this form, a phenomenon unique to Anomura not evident in other decapod lineages (e.g., lobsters, shrimp). The emergence of the crab-like form is not ‘evenly distributed’ across our phylogeny, first occurring in the older lineages Porcellanidae and Lomisidae and only more recently within the superfamily Lithodoidea. Some questions naturally arise. Why did carcinization occur independently three times during the evolution of the Anomura? Why did the presumably shell-dwelling asymmetrical hermit crab ancestors of lithodid king crabs forsake the use of shells for protection, which already provided them with survival advantage? Morrison et al. [33] suggest that the crab-like form might represent a key innovation that is associated with an evolutionary advantage, possibly due to the greater mobility and agility provided by this morphology. This seems to be evident within the true crabs, or Brachyura, which dominate decapods in terms of species richness [>6,559 species; 34] and have thrived in marine, freshwater, and terrestrial environments. Although diversification seems to be low in the crab-like anomurans when compared to the brachyurans, fossil evidence and divergence time analyses suggest crab-like anomurans are much younger when compared to the closely related true crabs (Table 2, Figure 4). Furthermore, the crab-like porcellanids are one of the oldest (~172 MYA, MRCA = 139 MYA) and most diverse families of anomurans [~247 species, 22]. Lithodids represent an even younger lineage, originating ~18 MYA, but comprising over 100 extant species. It is plausible that a crab-like form may hold some evolutionary advantage when considering age and diversification within Anomura, although this does not seem to hold true for all groups that underwent the crab-like transition (i.e., monotypic family Lomisidae). A second hypothesis explains the possible advantage of carcinization from a hermit-like ancestor. Previous studies have suggested a free-living body form may have a selective advantage in obtaining food resources when unconstrained by a gastropod shell [54,73]. An example can be seen in the semi-terrestrial hermit crab, *Birgus latro*, a species that in the adult stages has lost dependence on shells as protection for the pleon, and instead has developed a calcified body [74].

### Diversification rates

The extraordinary morphological and ecological diversity of anomurans has long fascinated evolutionary biologists. Previous studies covering a wide range of faunas have shown how morphological or ecological factors may influence the course of subsequent evolutionary diversification [75-77], and in particular for anomurans it has been hypothesized that the acquisition of the crab-like form may have acted as a key-innovation [33].

Our analysis reports the pattern of diversification in Anomura to be characterized by a low net rate of diversification, with two major changes in the rates of speciation along its evolutionary history. The initial diversification of the group during the Late Permian was characterized by slow rates of diversification and it was not concomitant with major family radiations, which took place from the Jurassic onwards.

A significant change in the tempo of diversification was identified within the speciose squat-lobster family Chirostylidae, which has a higher speciation rate than the overall tempo of diversification across the anomuran tree (Figure 4). Recent studies based on the munidid squat-lobster genus *Paramunida* suggest that dramatic environmental change may provide great geological and habitat complexity, which in turn promotes isolation and rapid diversification [78]. The fact that both families, chirostylids and munidids, diverged during the Late Triassic (see Figure 4) and currently occupy deep-sea habitats suggests that similar geological and environmental changes may also have driven major diversification within the Munididae, which shifted habitats at some point because the Jurassic forms are nearly all coral-reef associated. Currently, the family Chirostylidae accounts for 7% of all anomuran species, but the true diversity is underestimated and about 100 new species are in hand of taxonomists [79]. Clearly, a more accurate phylogenetic framework is needed to interpret in detail the exceptionally high speciation rates reported here.

The monotypic family Lomisidae showed a strikingly lower rate than the overall tempo of diversification in Anomura. *Lomis hirta* is anomalous in its prolonged persistence despite an inferred speciation rate of zero (as recovered by the MEDUSA analysis, see Results). These taxa, old lineages with few extant species, have been reported in several invertebrates and vertebrates [40,80,81] and more recently in butterflies [82], suggesting that extremely low rates of diversification characterize these groups. High extinction rates could also account for this pattern; however, we report that a pure-birth Yule model best explains our data. Under a high-extinction scenario we would expect to see an overabundance of more recently arisen species that simply have not yet gone extinct; such a pattern is not observed in our phylogeny.

Our analysis failed to identify a correlation between the timing of branching events (speciation) and the evolutionary history of carcinized lineages, which suggests that the acquisition of a crab-like form did not play a major role in shaping extant anomuran biodiversity. However, a major limitation of the MEDUSA approach is that rate shifts cannot be assigned below the level of phylogenetic resolution [40], which prevents us from evaluating if the highly carcinized family Lithodidae underwent an unusual rapid diversification event. The lack of a rate shift in the branch leading to the three collapsed families (Paguridae, Hapalogastridae, and Lithodidae) does not necessarily imply that subclades within that group have not experienced changes in the tempo of diversification, which may be masked by the lack of taxonomic resolution among these taxa. Thus, further studies which focus on clarifying the systematics of the infraorder, with particular emphasis on the families Paguridae, Hapalogastridae, and Lithodidae, are necessary to examine the role of carcinization in anomuran diversification.

## Conclusions

Anomuran relationships have been the topic of long debate, likely because of their extraordinary morphological and ecological diversity and their common targeting in fisheries. Here we estimate evolutionary relationships among 19 families, 7 superfamilies, and 137 species of anomurans based on morphological and molecular data to provide the most robust anomuran phylogeny to date. Many families and genera appear to be poly- or paraphyletic suggesting further taxonomic revisions at these levels. Carcinization evolved multiple times during the evolution of Anomura whereas transition into exclusively freshwater or semi-terrestrial environment occurred in the families Aeglidae and Coenobitidae, respectively. Divergence times date the origin of the group in the Late Permian, with subsequent radiations through the Jurassic and Cretaceous. Results suggest that anomurans diversified under low speciation rates with two major changes in the tempo of diversification. First insights suggest that the acquisition of the crab-like form did not play a major role in shaping the extant diversity of Anomura, but further examination is required in order to confirm this pattern.

## Methods

### Taxon sampling

Our study included extant representatives from 19 families, 77 genera, and 137 species of anomurans. The exceptionally rare family Pylojaquesidae is excluded for lack of molecular grade tissue samples. A total of 345 sequences from 76 of 144 anomuran specimens were new to this study, while sequences for all five genes from 68 taxa were obtained from GenBank. Newly included specimens were collected on cruise and field expeditions, from collaborators, or from the University of Louisiana at Lafayette Zoological (ULLZ) collection of molecular grade specimen and tissue samples (Table 1). Specimens were stored in 80% ethyl alcohol.

The sister group of Anomura is widely accepted to be Brachyura [24-26,35-38], but because some molecular studies have recovered alternative arrangements [24,25,38] we included 18 outgroup taxa (see Table 1) spanning several decapod lineages. Different outgroups were included/excluded to explore sister relationships to Anomura and the impact of outgroup selection on anomuran relationships. They consist of representatives from infraorders Brachyura (5), Axiidea (4), Gebiidea (3), Caridea (4), and suborder Dendrobranchiata (2).

### Morphological matrix

Our morphological data matrix consisted of 156 characters and 154 species (including outgroups) and was constructed in MacClade 4.0 (see Additional files 2 and 3). Citations of previously recognized characters and states are given following characters in Additional file 3. Codings for somatic morphological characters were scored based on examination of sequenced species (Table 1) supplemented by literature. For spermatozoal (130–143) and larval characters (144–156) that are highly conserved (but not available for every sequenced species), reasonable assumptions of monophyly were made in order to optimize the potential contribution of these data. Thus, for these spermatozoal characters, all members of a particular family for which data were available for some members were scored as uniform. For larval characters (primarily first zoeal stage), all members of a particular genus for which data were available for some members were scored as uniform. The larval characters that could be meaningfully scored across the breadth of taxa were included. Others are typically invariant within the family-level (and often superfamily-level) clades, as defined by recent revisionary classifications, and could have been effectively scored at family level. In deference to the possibility that some families might not be monophyletic, however, we took a more conservative, genus-level approach to larval character scoring. Monophyly (or not) of genera, however, with respect to the first zoeal characters employed does not affect results because of the level of generality of characters operating at low taxonomic levels.

Missing data were scored as unknown (?) and polymorphisms were scored as such rather than assuming a plesiomorphic state. Just as alignment gaps in molecular data have been variously treated as a fifth position or as missing in different studies, inapplicable character states in the morphological data may be scored as missing or as an additional character state, ‘inapplicable’ [83]. We scored inapplicable character states as unknown (indicated by ‘-’), rather than an additional state, in order to avoid the possibility of nodes being supported by a non-existent character state [84].

### DNA extraction, PCR, sequencing, and next-generation approaches

Total genomic DNA was extracted from the pleon or gills using the QiagenDNeasy® Blood and Tissue Kit Cat. No. 69582. Two partial mitochondrial genes, 16S and 12S, were amplified by PCR using the following primers, respectively: L2/L9 & 16S1472 or 16SF & 16S1472 [~580 bps, [85-87]] and 12S1F & 12SR or 12SF & 12S1R [~350 bps, [88]]. The nuclear large subunit 28S rRNA was amplified in sections by 1.3 F/4b, 3.25/4.4b, sA/5b, and 4.8/6b [~2200 bps, [89,90]]. The nuclear small subunit 18S rRNA was amplified by A/L, C/Y, O/B [~1800 bps, [91,92]] or by 1 F/2.9, 0.7/bi, 2.0/9R [89,90], or by shorter internal primers (~1700 bps, B/D18s1R, D18s2F-D18s2R, D18s3F-D18s3R, D18s4F-D18s4R and D18s5F-A [93]). The histone H3 gene was amplified by H3AF/H3AR [~350 bps, [94]]. The majority of target gene regions were obtained through traditional Sanger sequencing and data for seven taxa were obtained through next-generation 454 sequencing (see below).

PCR amplifications were performed in 25 μl volumes containing 1 μl of Taq polymerase HotMaster or REDTaq, PCR buffer, 2.5 mM of deoxyribonucleotide triphosphate mix dNTPs, 0.5 μM forward and reverse primer, and extracted DNA. The thermal profile used an initial denaturation for 1 min at 94°C followed by 35–40 cycles of 30 sec at 94°C, 45 sec at 45-60°C depending on gene region, 1 min at 72º and a final extension of 10 min at 72°C. PCR products were purified using plate filters PrepEase™ PCR Purification 96-well Plate Kit, USB Corporation and sequenced with ABI BigDye® terminator mix (Applied Biosystems, Foster City, CA, USA). Cycle sequencing reactions were performed in an Applied Biosystems 9800 Fast Thermal Cycler (Applied Biosystems, Foster City, CA, USA), and sequencing products were run forward and reverse on an ABI 3730xl DNA Analyzer 96-capillary automated sequencer in the Brigham Young University (BYU) sequencing center.

Sequence data for seven taxa were obtained using a novel next-generation sequencing technique TAS: Targeted Amplification Sequencing on the 454 platform [95,96]. The process required a two-step PCR to prepare selected DNA regions for targeted/directed sequencing. The first PCR used a locus specific primer (e.g., 16S, 12S, etc.) with a 22 bp adapter. These amplicons were cleaned using plate filters PrepEase™ PCR Purification 96-well Plate Kit, USB Corporation. One μl of cleaned PCR product was used as template for the second PCR. PCR II incorporated a 10 bp barcode multiplex identifier, MID, 4 bp key, and a 21 bp 454 Titanium primer. Samples were again cleaned using the Millipore system and subsequently combined in emulsion PCR and sequenced via 454 GS FLX Titanium pyrosequencing technology (Roche) at the BYU sequencing center. The bioinformatic pipeline, BarcodeCruncher, allowed us to exclude short reads, trim adapters, identify contamination, parse barcoded sequences, and assembly consensus sequences for phylogenetic reconstruction [for full description of methods see [95,96]].

### Phylogenetic analyses

Sequences were cleaned and assembled using Sequencher 4.9 (GeneCodes, Ann Arbor, MI, USA). To check for pseudogenes, we followed suggestions by Song et al. (2008), which included extracting DNA from tissue with high amounts of mitochondrial gill tissue, translating protein-coding sequences H3 to check for indels and stop codons, comparing sequences among closely-related species, and building individual gene trees to ensure similar topologies [97]. Comparing gene trees and BLAST searches helped identify contamination. Two datasets were assembled: 1) molecular dataset including all 5 gene regions 2) combined dataset including molecular + morphological data.

Individual gene alignments were performed using MAFFT, implementing the “E-INS-i” option. For non-protein coding genes 12S, 16S, 18S, 28S, GBlocks v0.91b were used to exclude regions of the alignment with questionable positional homology [98]. The parameters used in GBlocks for 12S, 16S, 18S, 28S, were as follow: minimum number of sequences for a conserved position = 50/77/77/79; minimum number of sequences for a flanking position = 50/77/80/79; maximum number of contiguous non-conserved positions = 8/8/8/8; minimum length of a block = 5/5/5/5; allowed gap positions = half/half/half/half. Final alignments included 300, 474, 1632, and 931 base pairs for 12S, 16S, 18S, and 28S, respectively. After trimming for primer residue, the H3 alignment resulted in 332 base pairs. In MESQUITE [99], all genes were concatenated 3669 basepairs and partitioned for analysis. The final molecular dataset included 162 individuals as 3669 basepairs (5 genes) while the combined data set included the molecular dataset plus an additional 156 morphological characters.

The Maximum Likelihood (ML) analysis was conducted using RAxML Randomized Axelerated Maximum Likelihood [100-102]. Likelihood settings followed the General Time Reversible Model GTR with a gamma distribution and RAxML estimated all free parameters following a partitioned dataset. The first algorithm used in the analysis was the “-f a” option, for a rapid bootstrap analysis and search for the best tree in a single pass. The second algorithm implemented another search for the best tree implementing a “-f d” option of -#200 iterations of random starting trees. Likelihoods were compared to determine the best tree and bootstraps were mapped on the resulting topology. Confidence in the resulting topology was assessed using non-parametric bootstrap estimates [103] with 1000 pseudoreplicates and values > 50% are presented on the resulting phylogeny.

Bayesian analyses (BA) were performed in MrBayes v3.1.2b4 [104] for the molecular and combined datasets morphology + molecular. We used the Markov *k* Mk, [105] model for the morphological characters equal state frequencies, combined with gamma distributes rates across sites. The model of evolution that best fit the individual datasets was determined by MODELTEST 3.7 [106] and these parameters were applied to our molecular dataset. Three independent BA analyses were implemented each with 20 chains and a starting tree obtained from the ML analysis to help reach convergence. The molecular analysis ran for 30,000,000 generations, sampling one tree every 1000 generations. The combined analysis ran for 50,000,000 generations, sampling one tree every 5000 generations. To ensure that independent analyses converged on similar values, we graphically compared all likelihood parameters and scored means and variances using the program Tracer v1.4 [107]. Burn-in and stationary distributions were determined by observing the likelihood -LnL scores and split frequencies for the data (~10 million generations). A 50% majority-rule consensus tree was obtained from the remaining saved trees, once the data reached convergence. Posterior probabilities Pp for clades were compared for congruence and post-burn-in-trees were combined between individual runs. Values > 0.5 are presented on the BA phylogram presented as percentages. All analyses were run on Marylou6 Dell PowerEdge M610 computing cluster at Brigham Young University. High support is defined as ≥ 95/70 Pp/bs, marginal support is ≥ 85/65 and low support is ≤ 84/64.

### Alternative hypothesis testing

A partitioned S-H test [108] was used to test whether previous hypotheses of anomuran evolution implicit in modern, morphologically-based classifications [14-16] and morphological and/or molecular phylogenies [19,20,23] were significantly worse than our best ML tree. The test was implemented in RAxML using the same data partitions used to estimate our phylogeny. As in the ML analysis, the GTRGAMMA model was applied to each partition. Seven independent constrained tree topologies were constructed in Mesquite v.2.71 [99]. Topological constraints were forced to the following monophyletic clades: Diogenidae, Hapalogastridae, Lithodidae, Munididae, Paguridae, Paguroidea, and Pylochelidae. These clades were tested to examine the validity of current generic assignments by testing the poly- and paraphyly of the families and superfamilies in the tree. Lastly, to test the king to hermit hypothesis, we forced the topologies: 1) king crabs (Lithodidae) ancestral to hermit crab superfamily Paguroidea and 2) king crabs (Lithodidae) ancestral to hermit crab family Paguridae to test if these hypotheses are significantly worse than a “hermit to king” evolutionary pathway.

### Character evolution

We used ancestral state reconstruction (ASR) methods implemented in Mesquite v.2.71 [99] to examine character evolution across the anomurans. We traced evolutionary pathways of two characters: body form and habitat. Body form was assigned as follows: 0: crab-like (carcinized), 1: squat lobster form, 2: asymmetrical pleon, 3: symmetrical pleon. Habitat was assigned as 0: semi-terrestrial, 1: freshwater, 2: marine. These characters were optimized across our best estimate of anomuran relationships (=combined (molecular + morphology) Bayesian phylogeny). Because the importance of employing different methods for ASR has been documented, we used both maximum parsimony and maximum likelihood methods [109]. Likelihood methods are often preferred over parsimony reconstructions since they take into account branch lengths, all character state possibilities, and model evolution [110]. The model of evolution used in the maximum likelihood analysis was the Markov k-state 1 (Mk1) parameter model, which allows equal probability for any character state change. All characters were scored and compiled based on specimen observation and/or literature searches. Reconstructions among all outgroup taxa are not shown.

### Divergence time analyses

To estimate the relative timing of origins, diversification, body form evolution and transition in habitat, Bayesian molecular dating methods were implemented in BEAST v1.5.2 (Bayesian evolutionary analysis by sampling trees) [111]. BEAST allows for missing data, multiple calibration points, relaxed clock models, and increased flexibility of model parameters when compared to other dating methods (e.g., Multidivtime). Substitution and clock models were unlinked and the dataset was partitioned by gene following models of evolution generated by MODELTEST v3.7. A relaxed uncorrelated lognormal clock model and Yule speciation tree prior were selected. We recognize that there are varied models to consider when using relaxed dating methods. Simulation studies that have compared accuracy of relaxed clock methods have recovered conflicting results, with some favoring uncorrelated models [112], others favoring autocorrelated models [113] and some favoring both (autocorrelated and uncorrelated) depending on the dataset [114]. We chose an uncorrelated relaxed clock method due to the biological data under investigation and evidence from our divergence time analysis. It has been suggested that autocorrelation in life history traits (one biological assumption underlying autocorrelated relaxed clocks) would be less relevant in studies focused at high taxonomic levels, divergent taxa [112,115], and sparely sampled datasets [116]. We had little reason to believe we had autocorrelation in our anomuran dataset considering we were reconstructing a phylogeny across an infraorder that originated over 250 MYA (oldest fossil evidence = 201–228 MYA). Moreover, our sampling method focused at the superfamily and family level (and not within genera or species). Secondly, it has been suggested that rate autocorrelation can be measured by comparing the posterior and prior distributions of covariance in rates on neighboring branches [112,116]. All covariance estimates in our divergence time analyses suggested we had no strong evidence for autocorrelation of rates in our phylogeny. The statistic measure between parent and child branches contained values that span zero suggesting branches with slow and fast rates are adjacent on the tree. For abovementioned reasons, we did not assume autocorrelated rates across our tree. Our best estimate of phylogeny (=combined (morphology + molecular) tree) was used as a starting tree and the tree searching parameters were removed from the BEAUTI xml file.

Using the non-parametric rate smoothing algorithm in r8s [117], we made branch lengths proportional to the timing chronogram rather than the substitutions per site phylogram. This allowed the tree to adhere to the topological and temporal constraints of using fossil calibrations. We implemented two runs in BEAST with MCMC chain length of 350 million generations logging parameters every 3,500 samples. To ensure that analyses converged on similar values with acceptable mixing, likelihood stationary and burn-in values, we used the program Tracer v1.4 [107]. The runs were combined using LogCombiner [112]. Estimates of the mean divergence times with 95% highest posterior density regions HPD and posterior probabilities represented as percentages are noted on the chronogram. The BEAST analyses were performed on the Marylou6 Dell PowerEdge M610 computing cluster at Brigham Young University.

### Fossil and time calibrations

A total of 31 fossils were included in the analysis. We included fossils that represented the oldest known specimen for a particular family and/or genus (Table 2). Only fossils that could be confidently assigned to clades based on personal observation and/or previous literature were included in the analysis [[118], Table 2]. We followed recommendations by Parham et al. [119] when justifying fossil placement. Both deep and shallow fossil representatives were included. All fossils were placed at the crown (i.e., most recent common ancestor MRCA) or stem (i.e., node directly preceding the MRCA node of the clade). There was disagreement among authors about the familial assignment of *Juracrista*, as either a munidid or galatheid. The munidids and galatheids, however, are closely related so the affect on calibration is minimal. Therefore, we have followed the original taxonomy [120] and retained *Juracrista* in Munididae. Because divergence time should predate the fossil occurrence, all calibrations followed an exponential prior with the offset value set to the minimum calibration age. This distribution is suitable for modeling fossil calibrations, because it allows us to avoid a hard upper bound while providing an increased probability with the age range of fossil discovery [121].

### Diversification rates

We analysed patterns of diversification along the anomuran tree using a reworked version of MEDUSA [40]http://www.webpages.uidaho.edu/~lukeh/software/index.html. This method infers clade-specific changes in the tempo of diversification (rate acceleration and rate reduction) across a tree from phylogenetic branch lengths and taxonomic extant species richness information, the latter to account for incomplete taxon sampling [77]. MEDUSA utilized a stepwise AIC approach to adding clade-wise diversification models Yule or birth-death to a tree until the decrease in AIC failed to exceed a set threshold, which was dependent on tree size. Diversification analysis implemented in MEDUSA required that in the analysed tree terminal tips represented monophyletic taxonomic groups with known species richness. We attempted to resolve clades to the taxonomic level of family; however our best phylogenetic hypothesis did not support the monophyly of the families Munididae, Diogenidae, and Paguridae (Figure 2). This non-monophyly was also observed in molecular trees, (Additional file 4). Therefore, assuming the current family classification would lead us to infer shifts in diversification rates inconsistent with the phylogenetic relationships found in this study. Recent studies have suggested that further subdivisions are conceivable within Munididae [21], with the recognition of a well-differentiated clade including the genera *Munida*, *Cervimunida*, and *Pleuroncode*s and a second clade including the rest of the genera [19,122,123]. Our study highly supports the existence of the *Munida*, *Cervimunida,* and *Pleuroncode*s clade (Pp = 100), yielding also moderate support for the inclusion of the genus *Sadayoshia* (Pp = 91). Hence, species richness within the family Munididae was assigned to two different monophyletic subclades (see Additional file 4). In agreement with our data, Diogenidae has been recently recovered as paraphyletic [20]. Previous studies have showed the genera *Paguristes* or *Areopaguristes* to be separated from other diogenids [19,33,95,124], and closely related to *Pseudopaguristes* and *Tetralobistes*[125]. Thus, in order to assign a known species richness value, we pruned this clade to include all the species belonging to those 4 genera, and recognized a second clade to accommodate the rest of species of Diogenidae plus Coenobitidae (See Additional file 4). Finally, the family Paguridae is recognized as one of the most challenging groups within Anomura and its monophyletic origin (in relation to Lithodidae and Hapalogastridae) has been debated [19-21]. Given the high species richness of this family (~ 542 species) and the lack of an appropriate taxonomic framework, it is not possible to account for the phylogenetic position of each unsampled extant species or all possible lineages. Thus, subdividing this clade into smaller subclades to assign species richness would be arbitrary, potentially leading to spurious results. Although suboptimal, the family Paguridae was collapsed together with the families Hapalaogastridae and Lithodidae in order to satisfy the monophyly assumption of MEDUSA.

We obtained information about species richness for major lineages of Anomura using the most recent published checklists [22,44,46,126-130]. This information was assigned to 18 lineages of our phylogeny after pruning terminals belonging to the same monophyletic groups according to the aforementioned criteria (see Additional file 4).

## Abbreviations

MRCA: Most recent common ancestor; MYA: Million years ago; YA: Years ago.

## Competing interests

The authors declare that they have no competing interests.

## Authors’ contributions

HBG was the primary author of the paper and lead the phylogenetic, divergence time, and character reconstruction analyses. MEC helped sequence all the material used in the phylogenetic analysis and contributed to the intellectual merit of the paper. PC led the diversification analysis and provided valuable advice on other analyses and text. RMF and CES helped compile a list of fossils for the divergence time analysis, provided advice on fossil Anomura, and consulted with HBG on calibration assignment. STA coded all characters in the morphological matrix and provided insight into anomuran evolution. DLF and KAC conceptualized the project, obtained funding to conduct the study, provided specimens for the anomuran phylogeny and aided analyses and compilation of the manuscript. RL was instrumental in explaining the evolution of carcinization across the anomuran and hermit crab evolutionary relationships. All authors read and approved the final manuscript.

## Supplementary Material

Additional file 1 Excluded species list indicating the species, gene, accession number, and explanation sequence data was not included in the analysis. Click here for file

Additional file 2 Morphomatrix used in combined Bayesian analysis. Click here for file

Additional file 3 Morphological characters and states used in combined Bayesian analysis. Click here for file

Additional file 4 Constrained taxonomic groups for TurboMEDUSA analysis. Click here for file
