# Supplementary material for: A comprehensive and integrative reconstruction of evolutionary history for Anomura (Crustacea: Decapoda)
Source: BMC Evol Biol. 2013 Jun 20;13:128. doi: 10.1186/1471-2148-13-128 (PMC3708748; doi:10.1186/1471-2148-13-128)
Supplement: Additional file 2 — Morphomatrix used in combined Bayesian analysis. [file 1471-2148-13-128-S2.pdf]

Species	1	2	3	4	5	6	7	8	9	10	11	12	13	14	15	16	17	18	19	20
Solenocera sp.	0	0	0	0	0	0	0	0	0	0	0	1	0	0	1	0	1	1	0	0
Hymenopenaeus debilis	0	0	0	0	0	0	0	0	0	0	0	1	0	0	1	0	1	1	0	0
Atyopsis sp.	0	0	0	0	0	1	0	0	0	0	0	1	0	0	0	0	0	1	0	0
Latreutes fucorum	0	0	0	0	0	1	0	0	0	0	0	1	0	0	0	0	0	1	0	0
Ogyrides sp.	0	0	0	0	0	1	0	0	0	0	0	1	0	0	0	0	1	1	0	0
Palaemonetes pugio	0	0	0	0	0	1	0	0	0	0	0	1	0	0	0	0	1	1	1	0
Calastacus crosnieri	0	0	0	0	0	0	0	0	0	0	0	1	0	0	1	0	0	1	0	0
Calaxius manningi	0	0	0	0	0	0	0	0	0	0	0	1	0	0	1	0	0	1	0	0
Lepidophthalmus louisianensis	0	0	0	0	0	0	0	0	0	0	0	1	0	0	0	0	0	1	0	1
Sergio mericeae	1	-	0	0	0	0	0	0	0	0	0	1	0	0	0	0	0	1	0	1
Austinogebia narutensis	0	0	0	0	1	0	0	0	0	0	0	1	0	0	1	0	0	1	0	1
Laemedia astacina	0	2	0	0	1	0	0	0	0	0	0	1	0	0	0	0	0	0	0	1
Thalassina anomala	0	0	0	0	1	0	0	0	0	0	0	1	0	0	0	0	1	0	0	1
Cosmonotus grayi	1	-	0	1	0	1	0	0	0	0	1	0	0	0	0	0	0	1	0	0
Calappa gallus	1	-	0	1	1	1	0	1	0	0	1	1	0	1	0	0	0	1	0	0
Chorilia longipes	0	-	0	1	1	1	0	1	0	0	1	1	0	1	0	0	0	0	0	0
Cyclograpsus cinereus	1	-	0	1	1	1	0	1	0	0	1	1	0	1	0	0	0	1	0	0
Praebebalia longidactyla	1	-	0	1	1	1	0	1	0	0	1	1	0	1	0	0	0	1	0	0
Blepharipoda occidentalis	0	2	0	1	1	0	0	0	0	0	1	0	1	1	0	0	0	1	0	1
Emerita emeritus	0	2	0	0	0	0	0	0	0	0	0	1	0	0	0	0	0	1	0	1
Emerita brasiliensis	0	2	0	0	0	0	0	0	0	0	0	1	0	0	0	0	0	1	0	1
Emerita talpoida	0	2	0	0	0	0	0	0	0	0	0	1	0	0	0	0	0	1	0	1
Albunea gibbesii	0	0	0	0	1	0	0	1	0	0	1	1	0	0	0	0	0	1	0	1
Albunea catherinae	0	0	0	0	1	0	0	1	0	0	1	1	0	0	0	0	0	1	0	1
Zygopa michaelis	1	-	0	0	1	0	0	1	0	0	1	1	0	0	0	0	0	1	0	1
Lepidopa californica	0	2	0	0	1	0	0	1	0	0	1	1	0	0	0	0	0	1	0	1
Lepidopa dexterae	0	2	0	0	1	0	0	1	0	0	1	1	0	0	0	0	0	1	0	1
Paraleucolepidopa	0	2	0	0	1	0	0	1	0	0	1	1	0	0	0	0	0	1	0	1
Coenobita compressus	1	-	0	1	1	0	0	0	0	0	1	2	1	0	0	0	0	1	0	1
Coenobita clypeatus	1	-	0	1	1	0	0	0	0	0	1	2	1	0	0	0	0	1	0	1
Coenobita perlatus	1	-	0	1	1	0	0	0	0	0	1	2	1	0	0	0	0	1	0	1
Birgus latro	0	2	0	1	1	0	0	0	0	0	1	2	1	0	0	0	0	1	0	1
Clibanarius albidigitus	1	-	0	0	0	0	0	0	0	1	1	1	2	0	0	0	0	1	0	1
Cilbanarius antillensis	1	-	0	0	0	0	0	0	0	1	1	1	2	0	0	0	0	1	0	1
Clibanarius corallinus	1	-	0	0	0	0	0	0	0	1	1	1	2	0	0	0	0	1	0	1
Clibanarius vittatus	1	-	0	0	0	0	0	0	0	1	1	1	2	0	0	0	0	1	0	1
Isocheles pilosus	1	-	0	0	1	0	0	0	0	1	1	1	?	0	0	0	0	1	0	1
Isocheles wurdmenni	1	-	0	0	1	0	0	0	0	1	1	1	?	0	0	0	0	1	0	1
Calcinus obscurus	0	2	0	0	1	0	0	0	0	1	1	1	2	0	0	0	0	1	0	1
Calcinus laevimanus	0	2	0	0	1	0	0	0	0	1	1	1	2	0	0	0	0	1	0	1
Paguristes turgidus	0	2	0	0	1	0	0	0	0	1	1	1	2	0	0	0	0	1	0	1
Paguristes tortugae	0	2	0	0	1	0	0	0	0	1	1	1	2	0	0	0	0	1	0	1
Paguristes triangulatus	0	0	0	0	1	0	0	0	0	1	1	1	2	0	0	0	0	1	0	1
Paguristes moorei	1	-	0	0	1	0	0	0	0	1	1	1	2	0	0	0	0	1	0	1
Paguristes sericeus	0	0	0	0	1	0	0	0	0	1	1	1	2	0	0	0	0	1	0	1
Paguristes grayi	0	2	0	0	1	0	0	0	0	1	1	1	2	0	0	0	0	1	0	1
Paguristes puncticeps	0	2	0	0	1	0	0	0	0	1	1	1	2	0	0	0	0	1	0	1
Paguristes cadenati	0	2	0	0	1	0	0	0	0	1	1	1	2	0	0	0	0	1	0	1
Areopaguristes hewatti	0	2	0	0	1	0	0	0	0	1	1	1	2	0	0	0	0	1	0	1
Areopaguristes hewatti	0	2	0	0	1	0	0	0	0	1	1	1	2	0	0	0	0	1	0	1
Areopaguristes hewatti	0	2	0	0	1	0	0	0	0	1	1	1	2	0	0	0	0	1	0	1
Areopaguristes pilosus	0	2	0	0	1	0	0	0	0	1	1	1	2	0	0	0	0	1	0	1
Areopaguristes hummi	0	2	0	0	1	0	0	0	0	1	1	1	2	0	0	0	0	1	0	1
Areopaguristes hummi	0	2	0	0	1	0	0	0	0	1	1	1	2	0	0	0	0	1	0	1
Dardanus fuscous	1	-	0	0	1	0	0	0	0	1	1	1	1	0	0	0	0	1	0	1
Dardanus insignis	1	-	0	0	1	0	0	0	0	1	1	1	1	0	0	0	0	1	0	1
Dardanus sp.	1	-	0	0	1	0	0	0	0	1	1	1	1	0	0	0	0	1	0	1
Petrochirus diogenes	1	-	0	0	1	0	0	0	0	1	1	1	1	0	0	0	0	1	0	1

Species	1	2	3	4	5	6	7	8	9	10	11	12	13	14	15	16	17	18	19	20
Lithodes santolla	0	0	1	1	2	0	1	1	0	0	1	2	0	1	0	0	0	0	0	1
Lithodes santolla	0	0	1	1	2	0	1	1	0	0	1	2	0	1	0	0	0	0	0	1
Glyptolithodes cristatipes	0	0	1	1	2	0	0	1	0	0	1	2	0	1	0	0	0	0	0	1
Paralomis sp.	0	1	0	1	2	0	1	1	0	0	1	2	0	1	0	0	0	0	0	1
Phyllolithodes papillosus	0	1	0	1	2	0	1	1	0	0	1	2	0	1	0	0	0	0	0	1
Lopholithodes mandtii	0	1	0	1	2	0	0	1	0	0	1	2	0	1	0	0	0	0	0	1
Paralithodes brevipes	0	1	0	1	2	0	0	1	0	0	1	2	0	1	0	0	0	0	0	1
Paralithodes camtschaticus	0	1	0	1	2	0	0	1	0	0	1	2	0	1	0	0	0	0	0	1
Paralithodes platypus	0	1	0	1	2	0	0	1	0	0	1	2	0	1	0	0	0	0	0	1
Cryptolithodes sp.	0	2	0	1	2	0	0	1	0	0	1	1	0	1	0	0	0	0	0	1
Oedignathus inermis	0	2	0	1	1	0	0	1	0	0	1	1	1	1	0	0	0	1	0	1
Hapalogaster mertensii	0	2	0	1	1	0	0	1	0	0	1	1	1	1	0	0	0	1	0	1
Pagurus bernhardus	1	-	0	0	1	0	0	0	0	1	1	1	2	0	0	0	0	1	0	1
Pagurus stimpsoni	1	-	0	0	1	0	0	0	0	1	1	1	2	0	0	0	0	1	0	1
Pagurus carolinensis	1	-	0	0	1	0	0	0	0	1	1	1	2	0	0	0	0	1	0	1
Pagurus brevidactylus	1	-	0	0	1	0	0	0	0	1	1	1	2	0	0	0	0	1	0	1
Pagurus maclaughlinae	1	-	0	0	1	0	0	0	0	1	1	1	2	0	0	0	0	1	0	1
Pagurus pollicaris	1	-	0	0	1	0	0	0	0	1	1	1	2	0	0	0	0	1	0	1
Pagurus bullisi	1	-	0	0	1	0	0	0	0	1	1	1	2	0	0	0	0	1	0	1
Iridopagurus caribbensis	1	-	0	0	1	0	0	0	0	1	1	1	2	0	0	0	0	1	0	1
Iridopagurus reticulatus	1	-	0	0	1	0	0	0	0	1	1	1	2	0	0	0	0	1	0	1
Xylopagurus cancellarius	0	2	0	0	1	0	0	0	0	1	0	1	2	0	0	0	0	1	0	1
Labidochirus splendescens	0	2	0	0	2	0	0	1	0	0	1	1	2	0	0	0	0	0	0	1
Porcellanopagurus filholi	0	2	0	1	2	0	0	1	0	0	1	1	2	1	0	0	0	0	0	1
Gorepagurus piercei	1	-	0	0	1	0	0	0	0	1	1	1	2	0	0	0	0	1	0	1
Manucomplanus ungulatus	1	-	0	0	1	0	0	0	0	1	1	1	2	0	0	0	0	1	0	1
Pylopagurus discoidalis	0	2	0	0	1	0	0	0	0	1	0	1	2	0	0	0	0	1	0	1
Pylopaguridium markhami	0	2	0	0	1	0	0	0	0	1	1	1	2	0	0	0	0	1	0	1
Phimochirus holthuisi	0	2	0	0	1	0	0	0	0	1	1	1	2	0	0	0	0	1	0	1
Phimochirus randalli	0	2	0	0	1	0	0	0	0	1	1	1	2	0	0	0	0	1	0	1
Phimochirus randalli	0	2	0	0	1	0	0	0	0	1	1	1	2	0	0	0	0	1	0	1
Agaricochirus alexandri	1	-	0	0	1	0	0	0	0	1	1	1	2	0	0	0	0	1	0	1
Tomopagurus merimaculosus	1	-	0	0	1	0	0	0	0	1	1	1	2	0	0	0	0	1	0	1
Discorsopagurus schmitti	1	-	0	0	1	0	0	0	0	1	1	1	2	0	0	0	0	1	0	1
Bythiopagurus macrocolus	1	-	0	0	1	0	0	0	0	1	1	1	2	0	0	0	0	1	0	1
Sympagurus dimorphus	1	-	0	0	1	0	0	0	1	1	1	1	2	0	0	0	0	1	0	1
Sympagurus acinops	1	-	0	0	1	0	0	0	1	1	1	1	2	0	0	0	0	1	0	1
Sympagurus pictus	1	-	0	0	1	0	0	0	1	1	1	1	2	0	0	0	0	1	0	1
Sympagurus sp.	1	-	0	0	1	0	0	0	1	1	1	1	2	0	0	0	0	1	0	1
Parapagurus latimanus	1	-	0	0	1	0	0	0	1	1	1	1	1	0	0	0	0	1	0	1
Trizocheles spinosus	1	-	0	0	1	0	0	0	0	1	0	1	1	0	0	0	0	1	0	1
Pomatocheles jeffreysii	1	-	0	0	1	0	0	0	0	1	0	1	1	0	0	0	0	1	0	1
Pachycheles haigae	0	4	0	1	0	1	0	1	0	0	1	1	1	0	0	0	0	0	0	1
Pachycheles rudis	0	4	0	1	0	1	0	1	0	0	1	1	1	0	0	0	0	0	0	1
Pachycheles rugimanus	0	4	0	1	0	1	0	1	0	0	1	1	1	0	0	0	0	0	0	1
Pachycheles ackleinus	0	4	0	1	0	1	0	1	0	0	1	1	1	0	0	0	0	0	0	1
Pachycheles pilosus	0	4	0	1	0	1	0	1	0	0	1	1	1	0	0	0	0	0	0	1
Polyonyx gibbesii	0	4	0	1	0	1	0	1	0	0	1	1	1	0	0	0	0	0	0	1
Pisidia magdalenensis	0	3	0	1	0	1	0	1	0	0	1	1	1	1	0	0	0	0	0	1
Megalobrachium poeyi	0	3	0	1	0	1	0	1	0	0	1	1	1	0	1	0	0	0	0	1
Petrolisthes armatus	0	3	0	1	0	1	0	1	0	0	1	2	1	0	0	0	0	1	0	1
Petrolisthes armatus	0	3	0	1	0	1	0	1	0	0	1	2	1	0	0	0	0	1	0	1
Petrolisthes laevigatus	0	3	0	1	0	1	0	1	0	0	1	2	1	0	0	0	0	1	0	1
Petrolisthes galathinus	0	3	0	1	0	1	0	1	0	0	1	2	1	0	0	0	0	1	0	1
Neopisosoma angustifrons	0	3	0	1	0	1	0	1	0	0	1	1	1	0	0	0	0	1	0	1
Parapetrolisthes tortugensis	0	3	0	1	0	1	0	1	0	0	1	1	1	0	0	0	0	1	0	1
Parapetrolisthes tortugensis	0	3	0	1	0	1	0	1	0	0	1	1	1	0	0	0	0	1	0	1
Allopetrolisthes spinifrons	0	2	0	1	0	1	0	1	0	0	1	1	1	0	1	0	0	1	0	1

Species	1	2	3	4	5	6	7	8	9	10	11	12	13	14	15	16	17	18	19	20
Porcellana sayana	0	2	0	1	0	1	0	1	0	0	1	1	1	0	1	0	0	0	0	1
Euceramus sp.	0	2	0	1	1	0	0	0	0	0	1	0	1	1	1	0	0	1	0	1
Munida subrugosa	0	0	0	1	1	0	0	0	0	0	1	0	1	1	2	0	0	1	0	1
Munida quadrispina	0	0	0	1	1	0	0	0	0	0	1	0	1	1	2	0	0	1	0	1
Munida iris	0	0	0	1	1	0	0	0	0	0	1	0	1	1	2	0	0	1	0	1
Munida pusilla	0	0	0	1	1	0	0	0	0	0	1	0	1	1	2	0	0	1	0	1
Pleuroncodes monodon	0	0	0	1	1	0	0	0	0	0	1	1	1	1	2	0	0	1	0	1
Cervimunida johni	0	0	0	1	1	0	0	0	0	0	1	1	1	1	2	0	0	1	0	1
Sadayoshia sp.	0	0	0	1	1	0	0	0	0	0	1	0	1	1	2	1	0	1	0	1
Babamunida kanaloa	0	0	0	1	1	0	0	0	0	0	1	0	1	1	2	0	0	1	0	1
Agononida procera	0	0	0	1	1	0	0	0	0	0	1	0	1	1	2	0	0	1	0	1
Neonida grandis	0	0	0	1	1	0	0	1	0	0	1	0	1	1	1	0	0	1	0	1
Anoplonida inermis	0	0	0	1	1	0	0	1	0	0	1	0	1	1	1	0	0	1	0	1
Bathymunida balssi	0	0	0	1	1	0	0	1	0	0	1	0	1	1	2	0	0	1	0	1
Alainius crosnieri	0	2	0	1	1	0	0	1	0	0	1	1	1	1	1	0	0	1	0	1
Galathea sp.	0	2	0	1	1	0	0	1	0	0	1	1	1	1	0	0	0	0	0	1
Galathea rostrata	0	2	0	1	1	0	0	1	0	0	1	1	1	1	0	0	0	0	0	1
Leiogalathea laevirostris	0	2	0	1	1	0	0	0	0	0	1	1	1	1	0	0	0	1	0	1
Shinkaia crosnieri	0	2	0	1	1	0	0	0	0	0	1	1	1	0	0	0	0	1	0	1
Munidopsis bairdii	0	2	0	1	1	0	0	0	0	0	1	0	1	1	0	0	0	1	0	1
Munidopsis erinacea	0	0	0	1	1	0	0	0	0	0	1	0	1	1	0	0	0	1	0	1
Pseudomunida fragilis	0	0	0	1	1	0	0	0	0	0	1	2	1	1	2	0	1	1	0	1
Galacantha rostrata	0	0	0	1	1	0	0	0	0	0	1	0	1	1	0	0	0	1	0	1
Galacantha valdiviae	0	0	0	1	1	0	0	0	0	0	1	0	1	1	0	0	0	1	0	1
Eumunida picta	0	0	0	1	1	0	0	0	0	0	1	2	1	1	2	1	1	1	0	1
Eumunida picta	0	0	0	1	1	0	0	0	0	0	1	2	1	1	2	1	1	1	0	1
Eumunida funambulus	0	0	0	1	1	0	0	0	0	0	1	2	1	1	2	1	1	1	0	1
Uroptychus spinirostris	0	0	0	0	1	0	1	0	0	0	1	1	1	1	0	0	0	0	0	1
Uroptychus nitidus	0	2	0	0	0	0	0	0	0	0	1	1	1	1	0	0	0	0	0	1
Uroptychus parvulus	0	2	0	0	0	1	0	0	0	0	1	1	1	1	0	0	0	0	0	1
Uroptychus scambus	0	2	0	0	0	1	0	1	0	0	1	1	1	1	0	0	0	0	0	1
Gastroptychus novaezelandiae	0	0	0	1	1	0	0	0	0	0	1	1	1	1	0	0	0	0	0	1
Gastroptychus rogeri	0	0	0	1	1	0	1	0	0	0	1	2	1	1	0	0	0	1	0	1
Gastroptychus spinifer	0	0	0	1	1	0	1	0	0	0	1	2	1	1	0	0	0	1	0	1
Chirostylus novaecaledoniae	1	-	0	1	1	0	0	0	0	0	1	1	1	1	0	0	0	0	0	1
Kiwa hirsuta	0	2	0	1	1	0	0	0	0	0	1	2	1	0	1	0	0	1	0	1
Aegla violacea	0	2	0	1	2	0	0	0	0	0	1	2	0	1	0	0	0	0	0	1
Aegla uruguyana	0	2	0	1	2	0	0	0	0	0	1	2	0	1	0	0	0	0	0	1
Aegla platensis	0	2	0	1	2	0	0	0	0	0	1	2	0	1	0	0	0	0	0	1
Aegla papudo	0	2	0	1	2	0	0	0	0	0	1	2	0	1	0	0	0	0	0	1
Aegla jarai	0	2	0	1	2	0	0	0	0	0	1	2	0	1	0	0	0	0	0	1
Aegla cholchol	0	2	0	1	2	0	0	0	0	0	1	2	0	1	0	0	0	0	0	1
Aegla camargoi	0	2	0	1	2	0	0	0	0	0	1	2	0	1	0	0	0	0	0	1
Aegla abtao	0	2	0	1	2	0	0	0	0	0	1	2	0	1	0	0	0	0	0	1
Aegla alacalufi	0	2	0	1	2	0	0	0	0	0	1	2	0	1	0	0	0	0	0	1
Lomis hirta	0	2	0	1	1	1	0	1	0	0	1	2	1	0	0	0	0	1	0	1

Species	21	22	23	24	25	26	27	28	29	30	31	32	33	34	35	36	37	38	39	40
Solenocera sp.	0	0	0	0	2	0	?	0	0	0	0	0	0	0	0	2	0	0	0	0
Hymenopenaeus debilis	0	0	0	0	2	0	?	0	0	0	0	0	0	0	0	2	0	0	0	0
Atyopsis sp.	0	0	0	0	2	0	?	0	0	0	0	0	0	0	0	1	0	0	0	0
Latreutes fucorum	0	0	0	0	2	0	?	0	0	0	0	0	0	0	0	1	0	0	0	0
Ogyrides sp.	0	0	0	0	2	0	?	0	0	0	0	0	0	0	0	1	0	0	0	0
Palaemonetes pugio	0	0	0	0	2	0	?	0	0	0	0	0	0	0	0	1	0	0	0	0
Calastacus crosnieri	0	0	0	0	2	0	?	0	0	0	0	0	1	0	0	1	0	0	0	0
Calaxius manningi	0	0	0	0	2	0	?	0	0	0	0	0	1	0	0	1	0	0	0	0
Lepidophthalmus louisianensis	0	0	0	0	2	0	?	0	0	0	0	0	1	0	0	0	0	0	0	0
Sergio mericeae	0	0	0	0	2	0	?	0	0	0	0	0	1	0	0	0	0	0	0	0
Austinogebia narutensis	0	0	0	0	2	0	?	0	0	0	0	0	1	0	0	0	0	0	0	0
Laemedia astacina	0	0	0	0	2	0	?	0	0	0	0	0	1	0	0	0	0	0	0	0
Thalassina anomala	0	0	0	0	2	0	?	0	0	0	0	0	1	0	0	0	0	0	0	0
Cosmonotus grayi	0	1	0	0	0	0	?	0	0	0	0	0	0	0	0	1	0	0	1	0
Calappa gallus	0	1	0	0	0	0	?	0	0	0	0	0	0	0	0	1	0	0	1	0
Chorilia longipes	0	1	0	0	0	0	?	0	0	0	0	0	0	0	0	1	0	0	1	0
Cyclograpsus cinereus	0	1	0	0	0	0	?	0	0	0	0	0	0	0	0	1	0	0	1	0
Praebebalia longidactyla	0	1	0	0	0	0	?	0	0	0	0	0	0	0	0	1	0	0	1	0
Blepharipoda occidentalis	1	0	0	1	0	0	?	0	0	0	2	1	1	0	0	0	0	0	1	0
Emerita emeritus	1	0	0	1	0	0	?	0	0	0	2	1	1	0	0	1	0	0	1	0
Emerita brasiliensis	1	0	0	1	0	0	?	0	0	0	2	1	1	0	0	1	0	0	1	0
Emerita talpoida	1	0	0	1	0	0	?	0	0	0	2	1	1	0	0	1	0	0	1	0
Albunea gibbesii	1	0	0	1	0	0	?	0	0	0	2	1	1	0	0	1	0	0	1	0
Albunea catherinae	1	0	0	1	0	0	?	0	0	0	2	1	1	0	0	1	0	0	1	0
Zygopa michaelis	1	0	0	1	0	0	?	0	0	0	2	1	1	0	0	1	0	0	1	0
Lepidopa californica	1	0	0	1	0	0	?	0	0	0	2	1	1	0	0	1	0	0	1	0
Lepidopa dexterae	1	0	0	1	0	0	?	0	0	0	2	1	1	0	0	1	0	0	1	0
Paraleucolepidopa	1	0	0	1	0	0	?	0	0	0	2	1	1	0	0	1	0	0	1	0
Coenobita compressus	1	0	0	0	0	0	?	0	0	1	2	1	1	0	0	1	1	0	1	1
Coenobita clypeatus	1	0	0	0	0	0	?	0	0	1	2	1	1	0	0	1	1	0	1	1
Coenobita perlatus	1	0	0	0	0	0	?	0	0	1	2	1	1	0	0	1	1	0	1	1
Birgus latro	1	0	0	0	0	0	?	0	0	1	2	1	1	0	0	1	1	0	1	1
Clibanarius albidigitus	1	0	0	0	0	0	?	0	0	1	2	1	1	0	0	1	0	1	1	1
Cilbanarius antillensis	1	0	0	0	0	0	?	0	0	1	2	1	1	0	0	1	0	1	1	1
Clibanarius corallinus	1	0	0	0	0	0	?	0	0	1	2	1	1	0	0	1	0	1	1	1
Clibanarius vittatus	1	0	0	0	0	0	?	0	0	1	2	1	1	0	0	1	0	1	1	1
Isocheles pilosus	1	0	0	0	0	0	?	0	0	?	2	1	1	0	0	1	0	1	1	1
Isocheles wurdmenni	1	0	0	0	0	0	?	0	0	?	2	1	1	0	0	1	0	1	1	1
Calcinus obscurus	1	0	0	0	0	0	?	0	0	2	2	1	1	0	0	1	0	1	1	1
Calcinus laevimanus	1	0	0	0	0	0	?	0	0	2	2	1	1	0	0	1	0	1	1	1
Paguristes turgidus	1	0	0	0	0	0	?	0	0	2	2	1	1	0	1	1	0	1	1	1
Paguristes tortugae	1	0	0	0	0	0	?	0	0	2	2	1	1	0	1	1	0	1	1	1
Paguristes triangulatus	1	0	0	0	0	0	?	0	0	2	2	1	1	0	1	1	0	1	1	1
Paguristes moorei	1	0	0	0	0	0	?	0	0	2	2	1	1	0	1	1	0	1	1	1
Paguristes sericeus	1	0	0	0	0	0	?	0	0	2	2	1	1	0	1	1	0	1	1	1
Paguristes grayi	1	0	0	0	0	0	?	0	0	2	2	1	1	0	1	1	0	1	1	1
Paguristes puncticeps	1	0	0	0	0	0	?	0	0	2	2	1	1	0	1	1	0	1	1	1
Paguristes cadenati	1	0	0	0	0	0	?	0	0	2	2	1	1	0	1	1	0	1	1	1
Areopaguristes hewatti	1	0	0	0	0	0	?	0	0	2	2	1	1	0	1	1	0	1	1	1
Areopaguristes hewatti	1	0	0	0	0	0	?	0	0	2	2	1	1	0	1	1	0	1	1	1
Areopaguristes hewatti	1	0	0	0	0	0	?	0	0	2	2	1	1	0	1	1	0	1	1	1
Areopaguristes pilosus	1	0	0	0	0	0	?	0	0	2	2	1	1	0	1	1	0	1	1	1
Areopaguristes hummi	1	0	0	0	0	0	?	0	0	2	2	1	1	0	1	1	0	1	1	1
Areopaguristes hummi	1	0	0	0	0	0	?	0	0	2	2	1	1	0	1	1	0	1	1	1
Dardanus fuscous	1	0	0	0	0	0	?	0	0	1	2	1	1	0	0	1	0	1	1	1
Dardanus insignis	1	0	0	0	0	0	?	0	0	1	2	1	1	0	0	1	0	1	1	1
Dardanus sp.	1	0	0	0	0	0	?	0	0	1	2	1	1	0	0	1	0	1	1	1
Petrochirus diogenes	1	0	0	0	0	0	?	0	0	1	2	1	1	0	0	1	0	1	1	1

Species	21	22	23	24	25	26	27	28	29	30	31	32	33	34	35	36	37	38	39	40
Lithodes santolla	1	1	0	1	0	0	?	0	0	0	1	1	1	0	0	1	0	0	1	1
Lithodes santolla	1	1	0	1	0	0	?	0	0	0	1	1	1	0	0	1	0	0	1	1
Glyptolithodes cristatipes	1	1	1	1	0	0	?	0	0	0	1	1	1	0	0	1	0	0	1	1
Paralomis sp.	1	1	1	1	0	0	?	0	0	0	1	1	1	0	0	1	0	0	1	1
Phyllolithodes papillosus	1	1	0	1	0	0	?	0	0	0	1	1	1	0	0	1	0	0	1	1
Lopholithodes mandtii	1	1	1	1	0	0	?	0	0	0	1	1	1	0	0	1	0	0	1	1
Paralithodes brevipes	1	1	0	1	0	0	?	0	0	0	1	1	1	0	0	1	0	0	1	1
Paralithodes camtschaticus	1	1	0	1	0	0	?	0	0	0	1	1	1	0	0	1	0	0	1	1
Paralithodes platypus	1	1	0	1	0	0	?	0	0	0	1	1	1	0	0	1	0	0	1	1
Cryptolithodes sp.	1	1	0	1	0	0	?	0	0	0	1	1	1	0	0	1	0	0	1	1
Oedignathus inermis	1	1	0	1	0	0	?	0	0	0	1	1	1	0	0	1	0	0	1	1
Hapalogaster mertensii	1	1	0	1	0	0	?	0	0	0	1	1	1	0	0	1	0	0	1	1
Pagurus bernhardus	1	0	0	1	0	0	?	0	0	2	2	1	1	1	0	1	0	1	1	1
Pagurus stimpsoni	1	0	0	1	0	0	?	0	0	1	2	1	1	1	0	1	0	1	1	1
Pagurus carolinensis	1	0	0	1	0	0	?	0	0	1&2	2	1	1	1	0	1	0	1	1	1
Pagurus brevidactylus	1	0	0	1	0	0	?	0	0	1&2	2	1	1	1	0	1	0	1	1	1
Pagurus maclaughlinae	1	0	0	1	0	0	?	0	0	1&2	2	1	1	1	0	1	0	1	1	1
Pagurus pollicaris	1	0	0	1	0	0	?	0	0	?	2	1	1	1	0	1	0	1	1	1
Pagurus bullisi	1	0	0	1	0	0	?	0	0	?	2	1	1	1	0	1	0	1	1	1
Iridopagurus caribbensis	1	0	0	1	0	0	?	0	0	2	2	1	1	1	0	1	0	1	1	1
Iridopagurus reticulatus	1	0	0	1	0	0	?	0	0	2	2	1	1	1	0	1	0	1	1	1
Xylopagurus cancellarius	1	0	0	1	0	0	?	0	0	3	2	1	1	1	0	1	0	1	0	0
Labidochirus splendescens	1	0	0	1	0	0	?	0	0	2	2	1	1	1	0	1	0	1	1	1
Porcellanopagurus filholi	1	0	0	1	0	0	?	0	0	2	2	1	1	0	0	1	0	1	0	0
Gorepagurus piercei	1	0	0	1	0	0	?	0	0	1	2	1	1	1	0	1	0	1	1	1
Manucomplanus ungulatus	1	0	0	1	0	0	?	0	0	2	2	1	1	1	0	1	0	1	1	1
Pylopagurus discoidalis	1	0	0	1	0	0	?	0	0	1	2	1	1	1	0	1	0	1	0	0
Pylopaguridium markhami	1	0	0	1	0	0	?	0	0	?	2	1	1	1	0	1	0	1	1	1
Phimochirus holthuisi	1	0	0	1	0	0	?	0	0	1	2	1	1	1	0	1	0	1	1	1
Phimochirus randalli	1	0	0	1	0	0	?	0	0	1	2	1	1	1	0	1	0	1	1	1
Phimochirus randalli	1	0	0	1	0	0	?	0	0	1	2	1	1	1	0	1	0	1	1	1
Agaricochirus alexandri	1	0	0	1	0	0	?	0	0	2	2	1	1	1	0	1	0	1	0	0
Tomopagurus merimaculosus	1	0	0	1	0	0	?	0	0	1	2	1	1	1	0	1	0	1	1	1
Discorsopagurus schmitti	1	0	0	1	0	0	?	0	0	1	2	1	1	0	0	1	0	1	0	0
Bythiopagurus macrocolus	1	0	0	1	0	0	?	0	0	1	2	1	1	0	0	1	0	1	1	1
Sympagurus dimorphus	1	0	0	1	0	0	?	0	0	1	2	1	1	0	0	1	0	1	1	1
Sympagurus acinops	1	0	0	1	0	0	?	0	0	1	2	1	1	0	0	1	0	1	1	1
Sympagurus pictus	1	0	0	1	0	0	?	0	0	1	2	1	1	0	0	1	0	1	1	1
Sympagurus sp.	1	0	0	1	0	0	?	0	0	1	2	1	1	0	0	1	0	1	1	1
Parapagurus latimanus	1	0	0	1	0	0	?	0	0	1	2	1	1	0	0	1	0	1	1	1
Trizocheles spinosus	1	0	0	0	0	0	?	0	0	0	2	1	1	0	0	1	0	0	0	0
Pomatocheles jeffreysii	1	0	0	0	0	0	?	0	0	0	2	1	1	0	0	1	0	0	0	0
Pachycheles haigae	1	1	0	0	0	0	?	0	0	0	0	1	1	0	0	1	0	0	1	0
Pachycheles rudis	1	1	0	0	0	0	?	0	0	0	0	1	1	0	0	1	0	0	1	0
Pachycheles rugimanus	1	1	0	0	0	0	?	0	0	0	0	1	1	0	0	1	0	0	1	0
Pachycheles ackleinus	1	1	0	0	0	0	?	0	0	0	0	1	1	0	0	1	0	0	1	0
Pachycheles pilosus	1	1	0	0	0	0	?	0	0	0	0	1	1	0	0	1	0	0	1	0
Polyonyx gibbesii	1	1	0	0	0	0	?	0	0	0	0	1	1	0	0	1	0	0	1	0
Pisidia magdalenensis	1	1	0	0	0	0	?	0	0	0	0	1	1	0	0	1	0	0	1	0
Megalobrachium poeyi	1	1	0	0	0	0	?	0	0	0	0	1	1	0	0	1	0	0	1	0
Petrolisthes armatus	1	1	0	0	0	0	?	0	0	0	0	1	1	0	0	1	0	0	1	0
Petrolisthes armatus	1	1	0	0	0	0	?	0	0	0	0	1	1	0	0	1	0	0	1	0
Petrolisthes laevigatus	1	1	0	0	0	0	?	0	0	0	0	1	1	0	0	1	0	0	1	0
Petrolisthes galathinus	1	1	0	0	0	0	?	0	0	0	0	1	1	0	0	1	0	0	1	0
Neopisosoma angustifrons	1	1	0	0	0	0	?	0	0	0	0	1	1	0	0	1	0	0	1	0
Parapetrolisthes tortugensis	1	1	0	0	0	0	?	0	0	0	0	1	1	0	0	1	0	0	1	0
Parapetrolisthes tortugensis	1	1	0	0	0	0	?	0	0	0	0	1	1	0	0	1	0	0	1	0
Allopetrolisthes spinifrons	1	1	0	0	0	0	?	0	0	0	0	1	1	0	0	1	0	0	1	0

Species	21	22	23	24	25	26	27	28	29	30	31	32	33	34	35	36	37	38	39	40
Porcellana sayana	1	1	0	0	0	0	?	0	0	0	0	1	1	0	0	1	0	0	1	0
Euceramus sp.	1	0	0	0	0	0	?	0	0	0	0	1	1	0	0	1	0	0	1	0
Munida subrugosa	1	1	0	0	0	0	?	0	0	0	0	1	1	0	0	1	0	0	1	0
Munida quadrispina	1	1	0	0	0	0	?	0	0	0	0	1	1	0	0	1	0	0	1	0
Munida iris	1	1	0	0	0	0	?	0	0	0	0	1	1	0	0	1	0	0	1	0
Munida pusilla	1	1	0	0	0	0	?	0	0	0	0	1	1	0	0	1	0	0	1	0
Pleuroncodes monodon	1	1	0	0	0	0	?	0	0	0	0	1	1	0	0	1	0	0	1	0
Cervimunida johni	1	1	0	0	0	0	?	0	0	0	0	1	1	0	0	1	0	0	1	0
Sadayoshia sp.	1	1	0	0	0	0	?	0	0	0	0	1	1	0	0	1	0	0	1	0
Babamunida kanaloa	1	1	0	0	0	0	?	0	0	0	0	1	1	0	0	1	0	0	1	0
Agononida procera	1	1	0	0	0	0	?	0	0	0	0	1	1	0	0	1	0	0	1	0
Neonida grandis	1	1	0	0	0	0	?	0	0	0	0	1	1	0	0	1	0	0	1	0
Anoplonida inermis	1	1	0	0	0	0	?	0	0	0	0	1	1	0	0	1	0	0	1	0
Bathymunida balssi	1	1	0	0	0	0	?	0	0	0	0	1	1	0	0	1	0	0	1	0
Alainius crosnieri	1	1	0	0	0	0	?	0	0	0	0	1	1	0	0	1	0	0	1	0
Galathea sp.	1	1	0	0	0	0	?	0	0	0	0	1	1	0	0	1	0	0	1	0
Galathea rostrata	1	1	0	0	0	0	?	0	0	0	0	1	1	0	0	1	0	0	1	0
Leiogalathea laevirostris	1	1	0	0	0	0	?	0	0	0	0	1	1	0	0	1	0	0	1	0
Shinkaia crosnieri	1	1	0	0	0	0	?	0	0	0	0	1	1	0	0	1	0	0	1	0
Munidopsis bairdii	1	1	0	0	0	0	?	0	0	0	0	1	1	0	0	1	0	0	1	0
Munidopsis erinacea	1	1	0	0	0	0	?	0	0	0	0	1	1	0	0	1	0	0	1	0
Pseudomunida fragilis	1	1	0	0	0	0	0	0	1	0	0	2	1	0	0	1	0	0	1	0
Galacantha rostrata	1	1	0	0	0	0	?	0	0	0	0	1	1	0	0	1	0	0	1	0
Galacantha valdiviae	1	1	0	0	0	0	?	0	0	0	0	1	1	0	0	1	0	0	1	0
Eumunida picta	1	1	0	0	0	0	0	0	1	0	0	2	1	0	0	1	0	0	1	0
Eumunida picta	1	1	0	0	0	0	0	0	1	0	0	2	1	0	0	1	0	0	1	0
Eumunida funambulus	1	1	0	0	0	0	0	0	1	0	0	2	1	0	0	1	0	0	1	0
Uroptychus spinirostris	1	1	0	1	2	1	1	0	1	0	0	2	1	0	0	1	0	0	1	0
Uroptychus nitidus	1	1	0	1	2	1	1	0	1	0	0	2	1	0	0	1	0	0	1	0
Uroptychus parvulus	1	1	0	1	2	1	1	0	1	0	0	2	1	0	0	1	0	0	1	0
Uroptychus scambus	1	1	0	1	2	1	1	0	0	0	0	2	1	0	0	1	0	0	1	0
Gastroptychus novaezelandiae	1	1	0	1	2	1	0	1	1	0	0	2	1	0	0	1	0	0	1	0
Gastroptychus rogeri	1	1	0	0	2	0	?	0	1	0	0	2	1	0	0	1	0	0	1	0
Gastroptychus spinifer	1	1	0	0	2	0	?	0	1	0	0	2	1	0	0	1	0	0	1	0
Chirostylus novaecaledoniae	1	1	0	1	2	1	0	1	1	0	0	2	1	0	0	1	0	0	1	0
Kiwa hirsuta	1	1	0	0	1	0	?	0	1	0	0	2	1	0	0	1	0	0	1	0
Aegla violacea	1	1	0	0	1	0	?	0	1	0	0	1	1	0	0	0	0	0	1	0
Aegla uruguyana	1	1	0	0	1	0	?	0	1	0	0	1	1	0	0	0	0	0	1	0
Aegla platensis	1	1	0	0	1	0	?	0	1	0	0	1	1	0	0	0	0	0	1	0
Aegla papudo	1	1	0	0	1	0	?	0	1	0	0	1	1	0	0	0	0	0	1	0
Aegla jarai	1	1	0	0	1	0	?	0	1	0	0	1	1	0	0	0	0	0	1	0
Aegla cholchol	1	1	0	0	1	0	?	0	1	0	0	1	1	0	0	0	0	0	1	0
Aegla camargoi	1	1	0	0	1	0	?	0	1	0	0	1	1	0	0	0	0	0	1	0
Aegla abtao	1	1	0	0	1	0	?	0	1	0	0	1	1	0	0	0	0	0	1	0
Aegla alacalufi	1	1	0	0	1	0	?	0	1	0	0	1	1	0	0	0	0	0	1	0
Lomis hirta	1	1	0	1	0	0	?	0	0	0	0	1	1	0	0	0	0	0	1	0

Species	41	42	43	44	45	46	47	48	49	50	51	52	53	54	55	56	57	58	59	60
Solenocera sp.	0	0	0	0	0	0	0	0	0	0	0	0	0	0	0	0	0	0	0	?
Hymenopenaeus debilis	0	0	0	0	0	0	0	0	0	0	0	0	0	0	0	0	0	0	0	?
Atyopsis sp.	0	0	0	0	0	1	0	0	0	0	0	0	0	0	0	0	0	0	0	?
Latreutes fucorum	0	0	0	0	0	1	0	0	0	0	0	0	0	0	0	0	0	0	0	?
Ogyrides sp.	0	0	0	0	0	1	0	0	0	0	0	0	0	0	0	0	0	0	0	?
Palaemonetes pugio	0	0	0	0	0	1	0	0	0	0	0	0	0	0	0	0	0	0	0	?
Calastacus crosnieri	0	1	0	1	0	0	0	0	0	1	0	0	0	0	0	0	0	0	0	?
Calaxius manningi	0	1	0	1	0	0	0	0	0	1	0	0	0	0	0	0	0	0	0	?
Lepidophthalmus louisianensis	0	1	0	1	0	0	0	0	2	1	0	0	0	0	0	0	0	0	0	?
Sergio mericeae	0	1	0	1	0	0	0	0	2	1	0	0	0	0	0	0	0	0	0	?
Austinogebia narutensis	0	1	0	1	0	0	0	0	1	1	0	0	0	0	0	0	0	0	0	?
Laemedia astacina	0	1	0	1	0	0	0	0	0	1	0	0	0	0	0	0	0	0	0	?
Thalassina anomala	0	1	0	1	0	0	0	0	0	1	0	0	0	0	0	0	1	0	0	?
Cosmonotus grayi	0	1	1	0	0	0	0	0	0	0	0	0	0	0	1	-	-	0	0	?
Calappa gallus	0	1	1	0	0	0	0	1	0	0	1	0	0	0	1	-	-	0	0	?
Chorilia longipes	0	1	1	0	0	0	0	1	0	0	1	0	0	0	1	-	-	0	0	?
Cyclograpsus cinereus	0	1	1	0	0	0	0	1	0	0	1	0	0	0	1	-	-	0	0	?
Praebebalia longidactyla	0	1	1	0	0	0	0	1	0	0	1	0	0	0	1	-	-	0	0	?
Blepharipoda occidentalis	0	1	0	1	1	0	0	0	2	2	0	0	1	1	0	0	2	0	0	?
Emerita emeritus	0	1	0	1	0	0	0	2	0	0	0	0	0	1	0	0	2	0	0	?
Emerita brasiliensis	0	1	0	1	0	0	0	2	0	0	0	0	0	1	0	0	2	0	0	?
Emerita talpoida	0	1	0	1	0	0	0	2	0	0	0	0	0	1	0	0	2	0	0	?
Albunea gibbesii	0	1	0	1	0	0	0	2	0	0	1	0	0	1	0	0	2	0	0	?
Albunea catherinae	0	1	0	1	0	0	0	2	1	0	1	0	0	1	0	0	2	0	0	?
Zygopa michaelis	0	1	0	1	0	0	0	2	0	0	1	0	0	1	0	0	2	0	1	?
Lepidopa californica	0	1	0	1	0	0	0	0	1	0	0	0	0	1	0	0	2	0	0	?
Lepidopa dexterae	0	1	0	1	0	0	0	0	1	0	0	0	0	1	0	0	2	0	0	?
Paraleucolepidopa	0	1	0	1	0	0	0	0	1	0	0	0	0	1	0	0	2	0	0	?
Coenobita compressus	2	1	2	0	2	0	0	2	2	1	0	0	1	0	0	1	2	0	0	0
Coenobita clypeatus	2	1	2	0	2	0	0	2	2	1	0	0	1	0	0	1	2	0	0	0
Coenobita perlatus	2	1	2	0	2	0	0	2	2	1	0	0	1	0	0	1	2	0	0	0
Birgus latro	1	1	0	0	0	0	0	0	2	1	0	0	1	0	0	1	2	0	0	0
Clibanarius albidigitus	2	1	2	0	2	0	0	2	2	1	0	0	1	0	0	1	2	0	0	1
Cilbanarius antillensis	2	1	2	0	2	0	0	2	2	1	0	0	1	0	0	1	2	0	0	1
Clibanarius corallinus	2	1	2	0	2	0	0	2	2	1	0	0	1	0	0	1	2	0	0	1
Clibanarius vittatus	2	1	2	0	2	0	0	2	2	1	0	0	1	0	0	1	2	0	0	1
Isocheles pilosus	2	1	2	0	2	0	2	2	2	1	0	0	0	0	0	1	2	0	0	0
Isocheles wurdmenni	2	1	2	0	2	0	2	2	2	1	0	0	0	0	0	1	2	0	0	0
Calcinus obscurus	2	1	2	0	2	0	2	2	2	1	0	0	1	0	0	1	2	0	0	0
Calcinus laevimanus	2	1	2	0	2	0	2	2	2	1	0	0	1	0	0	1	2	0	0	0
Paguristes turgidus	2	1	2	0	2	0	2	2	2	1	0	0	1	0	0	1	2	0	0	1
Paguristes tortugae	2	1	2	0	2	0	2	2	2	1	0	0	1	0	0	1	2	0	0	2
Paguristes triangulatus	2	1	2	0	2	0	2	2	2	1	0	0	1	0	0	1	2	0	0	0
Paguristes moorei	2	1	2	0	2	0	2	2	2	1	0	0	1	0	0	1	2	0	0	0
Paguristes sericeus	2	1	2	0	2	0	2	2	2	1	0	0	1	0	0	1	2	0	0	0
Paguristes grayi	2	1	2	0	2	0	2	2	2	1	0	0	1	0	0	1	2	0	0	0
Paguristes puncticeps	2	1	2	0	2	0	2	2	2	1	0	0	1	0	0	1	2	0	0	0
Paguristes cadenati	2	1	2	0	2	0	2	2	2	1	0	0	1	0	0	1	2	0	0	0
Areopaguristes hewatti	2	1	2	0	2	0	2	2	2	1	0	0	1	0	0	1	2	0	0	2
Areopaguristes hewatti	2	1	2	0	2	0	2	2	2	1	0	0	1	0	0	1	2	0	0	2
Areopaguristes hewatti	2	1	2	0	2	0	2	2	2	1	0	0	1	0	0	1	2	0	0	2
Areopaguristes pilosus	2	1	2	0	2	0	2	2	2	1	0	0	1	0	0	1	2	0	0	1
Areopaguristes hummi	2	1	2	0	2	0	2	2	2	1	0	0	1	0	0	1	2	0	0	1
Areopaguristes hummi	2	1	2	0	2	0	2	2	2	1	0	0	1	0	0	1	2	0	0	1
Dardanus fuscous	2	1	2	0	2	0	0	2	2	1	0	0	1	0	0	1	2	0	0	1
Dardanus insignis	2	1	2	0	2	0	0	2	2	1	0	0	1	0	0	1	2	0	0	1
Dardanus sp.	2	1	2	0	2	0	0	2	2	1	0	0	1	0	0	1	2	0	0	1
Petrochirus diogenes	2	1	2	0	2	0	0	2	?	?	0	?	?	0	?	1	2	0	0	0

Species	41	42	43	44	45	46	47	48	49	50	51	52	53	54	55	56	57	58	59	60
Lithodes santolla	1	1	1	0	0	0	3	1	2	2	0	0	0	0	1	0	-	0	0	?
Lithodes santolla	1	1	1	0	0	0	3	1	2	2	0	0	0	0	1	0	-	0	0	?
Glyptolithodes cristatipes	1	1	1	0	0	0	0	1	2	2	0	0	0	0	1	0	-	0	0	?
Paralomis sp.	1	1	1	0	0	0	1	1	2	2	0	0	0	0	1	0	-	0	0	?
Phyllolithodes papillosus	1	1	1	0	0	0	4	1	2	2	0	0	0	0	1	0	-	0	0	?
Lopholithodes mandtii	1	1	1	0	0	0	1	1	2	2	0	0	0	0	1	0	-	0	0	?
Paralithodes brevipes	1	1	1	0	0	0	1	1	2	2	0	0	0	0	1	0	-	0	0	?
Paralithodes camtschaticus	1	1	1	0	0	0	1	1	2	2	0	0	0	0	1	0	-	0	0	?
Paralithodes platypus	1	1	1	0	0	0	1	1	2	2	0	0	0	0	1	0	-	0	0	?
Cryptolithodes sp.	1	1	1	0	0	0	0	1	2	2	0	0	0	0	1	0	-	0	0	?
Oedignathus inermis	2	1	1	0	1	0	0	1	2	1	0	0	0	0	1	0	-	0	0	?
Hapalogaster mertensii	2	1	1	0	1	0	0	1	2	1	0	0	0	0	1	0	-	0	0	?
Pagurus bernhardus	2	1	2	0	2	0	2	2	2	1	0	0	1	0	0	1	2	0	0	0
Pagurus stimpsoni	2	1	2	0	2	0	2	2	2	1	0	0	1	0	0	1	2	0	0	0
Pagurus carolinensis	2	1	2	0	2	0	2	2	2	1	0	0	1	0	0	1	2	0	0	1
Pagurus brevidactylus	2	1	2	0	2	0	2	2	2	1	0	0	1	0	0	1	2	0	0	1
Pagurus maclaughlinae	2	1	2	0	2	0	2	2	2	1	0	0	1	0	0	1	2	0	0	0
Pagurus pollicaris	2	1	2	0	2	0	2	2	2	1	0	0	1	0	0	1	2	0	0	0
Pagurus bullisi	2	1	2	0	2	0	2	2	2	1	0	0	1	0	0	1	2	0	0	0
Iridopagurus caribbensis	2	1	2	0	2	0	2	2	1	1	0	0	1	0	0	1	2	0	0	0
Iridopagurus reticulatus	2	1	2	0	2	0	2	2	1	1	0	0	1	0	0	1	2	0	0	0
Xylopagurus cancellarius	2	1	2	0	2	0	2	2	0	1	0	0	0	0	0	1	2	0	0	1
Labidochirus splendescens	2	1	2	0	2	0	2	2	2	1	0	0	1	0	0	1	2	0	0	0
Porcellanopagurus filholi	2	1	2	0	1	0	0	2	2	1	0	0	1	0	0	1	2	0	0	0
Gorepagurus piercei	2	1	2	0	2	0	2	2	0	1	0	0	1	0	0	1	2	0	0	0
Manucomplanus ungulatus	2	1	2	0	2	0	2	2	1	1	0	0	1	0	0	1	2	0	0	0
Pylopagurus discoidalis	2	1	2	0	2	0	2	2	1	1	0	0	1	0	0	1	2	0	0	0
Pylopaguridium markhami	2	1	2	0	2	0	2	2	1	1	0	0	1	0	0	1	2	0	0	1
Phimochirus holthuisi	2	1	2	0	2	0	2	2	1	1	0	0	1	0	0	1	2	0	0	0
Phimochirus randalli	2	1	2	0	2	0	2	2	1	1	0	0	1	0	0	1	2	0	0	0
Phimochirus randalli	2	1	2	0	2	0	2	2	1	1	0	0	1	0	0	1	2	0	0	0
Agaricochirus alexandri	2	1	2	0	2	0	2	2	1	1	0	0	1	0	0	1	2	0	0	0
Tomopagurus merimaculosus	2	1	2	0	2	0	2	2	1	1	0	0	1	0	0	1	2	0	0	0
Discorsopagurus schmitti	2	1	2	0	2	0	0	2	2	1	0	0	0	0	0	1	2	0	0	0
Bythiopagurus macrocolus	2	1	2	0	2	0	2	2	2	2	0	0	1	0	0	1	2	0	0	0
Sympagurus dimorphus	2	1	2	0	2	0	2	2	2	1	0	0	1	0	0	1	2	0	0	0
Sympagurus acinops	2	1	2	0	2	0	2	2	2	1	0	0	1	0	0	1	2	0	0	0
Sympagurus pictus	2	1	2	0	2	0	2	2	2	1	0	0	1	0	0	1	2	0	0	0
Sympagurus sp.	2	1	2	0	2	0	2	2	2	1	0	0	1	0	0	1	2	0	0	0
Parapagurus latimanus	2	1	2	0	2	0	2	2	2	2	0	0	1	0	0	1	2	0	0	0
Trizocheles spinosus	0	1	0	0	0	0	0	0	0	1	0	0	1	?	0	1	2	0	0	0
Pomatocheles jeffreysii	0	1	0	0	0	0	0	0	1	1	0	3	1	?	0	1	2	0	0	?
Pachycheles haigae	0	1	0	1	0	0	0	0	2	0	0	1	0	1	0	0	0	0	0	?
Pachycheles rudis	0	1	0	1	0	0	0	0	2	0	0	1	0	1	0	0	0	0	0	?
Pachycheles rugimanus	0	1	0	1	0	0	0	0	2	0	0	1	0	1	0	0	0	0	0	?
Pachycheles ackleinus	0	1	0	1	0	0	0	0	2	0	0	1	0	1	0	0	0	0	0	?
Pachycheles pilosus	0	1	0	1	0	0	0	0	2	0	0	1	0	1	0	0	0	0	0	?
Polyonyx gibbesii	0	1	0	1	0	0	0	0	2	0	0	1	0	1	0	0	0	0	0	?
Pisidia magdalenensis	0	1	0	1	0	0	0	0	2	0	0	1	0	1	0	0	0	0	0	?
Megalobrachium poeyi	0	1	0	1	0	0	0	0	2	0	0	1	0	1	0	0	0	0	0	?
Petrolisthes armatus	0	1	0	1	0	0	0	0	2	0	0	1	0	1	0	0	0	0	0	?
Petrolisthes armatus	0	1	0	1	0	0	0	0	2	0	0	1	0	1	0	0	0	0	0	?
Petrolisthes laevigatus	0	1	0	1	0	0	0	0	2	0	0	1	0	1	0	0	0	0	0	?
Petrolisthes galathinus	0	1	0	1	0	0	0	0	2	0	0	1	0	1	0	0	0	0	0	?
Neopisosoma angustifrons	0	1	0	1	0	0	0	0	2	0	0	1	0	1	0	0	0	0	0	?
Parapetrolisthes tortugensis	0	1	0	1	0	0	0	0	2	0	0	1	0	1	0	0	0	0	0	?
Parapetrolisthes tortugensis	0	1	0	1	0	0	0	0	2	0	0	1	0	1	0	0	0	0	0	?
Allopetrolisthes spinifrons	0	1	0	1	0	0	0	0	2	0	0	1	0	1	0	0	0	0	0	?

Species	41	42	43	44	45	46	47	48	49	50	51	52	53	54	55	56	57	58	59	60
Porcellana sayana	0	1	0	1	0	0	0	0	2	0	0	1	0	1	0	0	0	0	0	?
Euceramus sp.	0	1	0	1	0	0	0	0	2	0	0	1	0	1	0	0	0	0	0	?
Munida subrugosa	0	1	0	1	0	0	0	0	2	0	0	1	0	1	0	0	0	0	0	?
Munida quadrispina	0	1	0	1	0	0	0	0	2	0	0	1	0	1	0	0	0	0	0	?
Munida iris	0	1	0	1	0	0	0	0	2	0	0	1	0	1	0	0	0	0	0	?
Munida pusilla	0	1	0	1	0	0	0	0	2	0	0	1	0	1	0	0	0	0	0	?
Pleuroncodes monodon	0	1	0	1	0	0	0	0	2	0	0	1	0	1	0	0	0	0	0	?
Cervimunida johni	0	1	0	1	0	0	0	0	2	0	0	1	0	1	0	0	0	0	0	?
Sadayoshia sp.	0	1	0	1	0	0	0	0	2	0	0	1	0	1	0	0	0	0	0	?
Babamunida kanaloa	0	1	0	1	0	0	0	0	2	0	0	1	0	1	0	0	0	0	0	?
Agononida procera	0	1	0	1	0	0	0	0	2	0	0	1	0	1	0	0	0	0	0	?
Neonida grandis	0	1	0	1	0	0	0	0	2	0	0	1	0	1	0	0	0	0	0	?
Anoplonida inermis	0	1	0	1	0	0	0	0	2	0	0	1	0	1	0	0	0	0	0	?
Bathymunida balssi	0	1	0	1	0	0	0	0	2	0	0	1	0	1	0	0	0	0	0	?
Alainius crosnieri	0	1	0	1	0	0	0	0	2	0	0	1	0	1	0	0	0	0	0	?
Galathea sp.	0	1	0	1	0	0	0	0	2	0	0	1	0	1	0	0	0	0	0	?
Galathea rostrata	0	1	0	1	0	0	0	0	2	0	0	1	0	1	0	0	0	0	0	?
Leiogalathea laevirostris	0	1	0	1	0	0	0	0	2	0	0	1	0	1	0	0	0	0	0	?
Shinkaia crosnieri	0	1	0	1	0	0	0	0	2	0	0	1	0	1	0	0	0	1	1	?
Munidopsis bairdii	0	1	0	1	0	0	0	0	2	0	0	1	0	1	0	0	0	1	0	?
Munidopsis erinacea	0	1	0	1	0	0	0	0	2	0	0	1	0	1	0	0	0	1	0	?
Pseudomunida fragilis	0	1	0	1	0	0	0	0	2	1	0	3	1	?	0	0	0	0	0	?
Galacantha rostrata	0	1	0	1	0	0	0	0	2	0	0	1	0	1	0	0	0	1	0	?
Galacantha valdiviae	0	1	0	1	0	0	0	0	2	0	0	1	0	1	0	0	0	1	0	?
Eumunida picta	0	1	0	1	0	0	0	0	2	1	0	3	1	?	0	0	0	0	0	?
Eumunida picta	0	1	0	1	0	0	0	0	2	1	0	3	1	?	0	0	0	0	0	?
Eumunida funambulus	0	1	0	1	0	0	0	0	2	1	0	3	1	?	0	0	0	0	0	?
Uroptychus spinirostris	0	1	0	1	0	0	0	0	2	1	0	3	1	?	0	0	0	0	0	?
Uroptychus nitidus	0	1	0	1	0	0	0	0	2	1	0	3	1	?	0	0	0	0	0	?
Uroptychus parvulus	0	1	0	1	0	0	0	0	2	1	0	3	1	?	0	0	0	0	0	?
Uroptychus scambus	0	1	0	1	0	0	0	0	2	1	0	3	1	?	0	0	0	0	0	?
Gastroptychus novaezelandiae	0	1	0	1	0	0	0	0	2	1	0	3	1	?	0	0	0	0	0	?
Gastroptychus rogeri	0	1	0	1	0	0	0	0	2	1	0	3	1	?	0	0	0	0	0	?
Gastroptychus spinifer	0	1	0	1	0	0	0	0	2	1	0	3	1	?	0	0	0	0	0	?
Chirostylus novaecaledoniae	0	1	0	1	0	0	0	0	2	1	0	3	0	?	0	0	0	0	0	?
Kiwa hirsuta	0	1	0	1	0	0	0	0	2	1	0	3	1	?	0	0	0	1	?	?
Aegla violacea	0	1	0	1	0	0	0	0	2	2	0	2	1	0	0	0	0	0	0	?
Aegla uruguyana	0	1	0	1	0	0	0	0	2	2	0	2	1	0	0	0	0	0	0	?
Aegla platensis	0	1	0	1	0	0	0	0	2	2	0	2	1	0	0	0	0	0	0	?
Aegla papudo	0	1	0	1	0	0	0	0	2	2	0	2	1	0	0	0	0	0	0	?
Aegla jarai	0	1	0	1	0	0	0	0	2	2	0	2	1	0	0	0	0	0	0	?
Aegla cholchol	0	1	0	1	0	0	0	0	2	2	0	2	1	0	0	0	0	0	0	?
Aegla camargoi	0	1	0	1	0	0	0	0	2	2	0	2	1	0	0	0	0	0	0	?
Aegla abtao	0	1	0	1	0	0	0	0	2	2	0	2	1	0	0	0	0	0	0	?
Aegla alacalufi	0	1	0	1	0	0	0	0	2	2	0	2	1	0	0	0	0	0	0	?
Lomis hirta	0	1	0	1	0	0	0	0	2	2	0	0	0	0	0	0	1	0	0	?

**Species**	**61**	**62**	**63**	**64**	**65**	**66**	**67**	**68**	**69**	**70**	**71**	**72**	**73**	**74**	**75**	**76**	**77**	**78**	**79**	**80**
Solenocera sp.	1	0	0	0	0	0	0	1	0	1	0	0	0	1	1	0	1	0	0	0
Hymenopenaeus debilis	1	0	0	0	0	0	0	1	0	1	0	0	0	1	1	0	1	0	0	0
Atyopsis sp.	1	0	0	0	0	0	0	1	2	1	0	1	?	?	?	0	1	0	0	1
Latreutes fucorum	1	0	0	0	0	0	0	1	2	1	0	1	?	1	1	0	1	0	0	1
Ogyrides sp.	1	0	0	1	0	0	0	1	0	1	0	1	?	1	1	0	1	0	0	1
Palaemonetes pugio	1	0	0	0	0	0	0	1	2	1	0	1	?	1	1	0	1	0	0	1
Calastacus crosnieri	1	0	0	0	0	0	0	1	?	?	0	0	?	1	1	0	0	0	0	0
Calaxius manningi	1	0	0	0	0	0	0	1	?	?	0	0	?	1	1	0	0	0	0	0
Lepidophthalmus louisianensis	1	0	0	0	1	0	0	1	0	1	0	0	1	1	1	1	1	0	0	1
Sergio mericeae	1	0	0	0	1	0	0	1	0	1	0	0	1	1	1	1	1	0	0	1
Austinogebia narutensis	1	0	0	0	0	0	0	1	0	?	0	0	0	1	1	0	0	0	0	0
Laemedia astacina	1	0	0	0	0	0	0	1	1	0	0	0	0	1	1	0	0	0	0	0
Thalassina anomala	1	0	0	0	0	0	0	1	0	1	0	0	0	1	1	0	0	0	0	0
Cosmonotus grayi	1	0	0	1	0	0	1	0	?	?	0	0	0	1	1	1	1	1	0	1
Calappa gallus	0	0	0	0	0	0	1	0	1	0	0	0	0	1	1	1	1	3	0	0
Chorilia longipes	0	0	0	0	0	0	1	0	1	0	0	0	0	1	1	1	1	1	0	0
Cyclograpsus cinereus	0	0	0	0	0	0	1	0	1	0	0	0	0	1	1	1	1	1	0	0
Praebebalia longidactyla	0	0	0	0	0	0	1	0	1	0	0	0	0	1	1	1	1	3	0	0
Blepharipoda occidentalis	1	0	0	1	0	0	1	0	0	1	1	0	1	1	0	0	0	0	0	0
Emerita emeritus	1	0	0	0	0	0	1	0	1	1	?	0	1	1	0	0	1	1	0	1
Emerita brasiliensis	1	0	0	0	0	0	1	0	1	1	?	0	1	1	0	0	1	1	0	1
Emerita talpoida	1	0	0	0	0	0	1	0	1	1	?	0	1	1	0	0	1	1	0	1
Albunea gibbesii	1	0	0	0	1	0	1	0	0	1	1	0	1	1	0	0	1	0	0	1
Albunea catherinae	1	0	0	0	1	0	1	0	0	1	1	0	1	1	0	0	1	0	0	1
Zygopa michaelis	1	0	0	0	1	0	1	0	0	1	1	0	1	1	0	0	1	0	0	1
Lepidopa californica	1	0	0	0	1	0	1	0	0	1	1	0	1	1	0	0	1	1	1	1
Lepidopa dexterae	1	0	0	0	1	0	1	0	0	1	1	0	1	1	0	0	1	1	1	1
Paraleucolepidopa	1	0	0	0	1	0	1	0	0	1	1	0	1	1	0	0	1	1	1	1
Coenobita compressus	1	0	0	0	2	1	1	0	1	1	0	0	1	0	0	0	0	0	0	0
Coenobita clypeatus	1	0	0	0	2	1	1	0	1	1	0	0	1	0	0	0	0	0	0	0
Coenobita perlatus	1	0	0	0	2	1	1	0	1	1	0	0	1	0	0	0	0	0	0	0
Birgus latro	1	1	0	0	2	1	1	0	?	?	?	0	1	0	0	0	0	0	0	0
Clibanarius albidigitus	1	0	0	1	0	1	1	0	0	1	1	0	0	0	0	0	0	0	0	0
Cilbanarius antillensis	1	0	0	1	0	1	1	0	0	1	1	0	0	0	0	0	0	0	0	0
Clibanarius corallinus	1	0	0	1	0	1	1	0	0	1	1	0	0	0	0	0	0	0	0	0
Clibanarius vittatus	1	0	0	1	0	1	1	0	0	1	1	0	0	0	0	0	0	0	0	0
Isocheles pilosus	1	0	0	1	0	1	?	?	?	?	?	0	?	0	0	0	0	0	0	0
Isocheles wurdmenni	1	0	0	1	0	1	?	?	?	?	?	0	?	0	0	0	0	0	0	0
Calcinus obscurus	1	0	0	1	0	1	1	0	0	1	1	0	0	0	0	0	0	0	0	0
Calcinus laevimanus	1	0	0	1	0	1	1	0	0	1	1	0	0	0	0	0	0	0	0	0
Paguristes turgidus	1	0	0	1	0	1	1	0	0	1	1	0	0	1	0	0	0	0	0	0
Paguristes tortugae	1	0	0	1	0	1	1	0	0	1	1	0	0	1	0	0	0	0	0	0
Paguristes triangulatus	1	0	0	1	0	1	1	0	0	1	1	0	0	1	0	0	0	0	0	0
Paguristes moorei	1	0	0	1	0	1	1	0	0	1	1	0	0	1	0	0	0	0	0	0
Paguristes sericeus	1	0	0	1	0	1	1	0	0	1	1	0	0	1	0	0	0	0	0	0
Paguristes grayi	1	0	0	1	0	1	1	0	0	1	1	0	0	1	0	0	0	0	0	0
Paguristes puncticeps	1	0	0	1	0	1	1	0	0	1	1	0	0	1	0	0	0	0	0	0
Paguristes cadenati	1	0	0	1	0	1	1	0	0	1	1	0	0	1	0	0	0	0	0	0
Areopaguristes hewatti	1	0	0	1	0	1	1	0	0	1	1	0	0	1	0	0	0	0	0	0
Areopaguristes hewatti	1	0	0	1	0	1	1	0	0	1	1	0	0	1	0	0	0	0	0	0
Areopaguristes hewatti	1	0	0	1	0	1	1	0	0	1	1	0	0	1	0	0	0	0	0	0
Areopaguristes pilosus	1	0	0	1	0	1	1	0	0	1	1	0	0	1	0	0	0	0	0	0
Areopaguristes hummi	1	0	0	1	0	1	1	0	0	1	1	0	0	1	0	0	0	0	0	0
Areopaguristes hummi	1	0	0	1	0	1	1	0	0	1	1	0	0	1	0	0	0	0	0	0
Dardanus fuscous	1	0	0	0	0	1	1	0	0	0	0	0	0	1	0	0	0	0	0	0
Dardanus insignis	1	0	0	0	0	1	1	0	0	0	0	0	0	1	0	0	0	0	0	0
Dardanus sp.	1	0	0	0	0	1	1	0	0	0	0	0	0	1	0	0	0	0	0	0
Petrochirus diogenes	1	0	0	1	0	1	1	0	0	0	0	0	0	0	0	0	0	0	0	0

Species	61	62	63	64	65	66	67	68	69	70	71	72	73	74	75	76	77	78	79	80
Lithodes santolla	1	0	0	0	0	0	1	0	0	0	0	0	0	0	0	0	0	0	0	0
Lithodes santolla	1	0	0	0	0	0	1	0	0	0	0	0	0	0	0	0	0	0	0	0
Glyptolithodes cristatipes	1	0	0	0	0	0	1	0	0	0	0	0	0	0	0	0	0	0	0	0
Paralomis sp.	1	0	0	0	0	0	1	0	0	0	0	0	0	0	0	0	0	0	0	0
Phyllolithodes papillosus	1	0	0	0	0	0	1	0	0	0	0	0	0	0	0	0	0	0	0	0
Lopholithodes mandtii	1	0	0	0	0	0	1	0	0	0	0	0	0	0	0	0	0	0	0	0
Paralithodes brevipes	1	0	0	0	0	0	1	0	0	0	0	0	0	0	0	0	0	0	0	0
Paralithodes camtschaticus	1	0	0	0	0	0	1	0	0	0	0	0	0	0	0	0	0	0	0	0
Paralithodes platypus	1	0	0	0	0	0	1	0	0	0	0	0	0	0	0	0	0	0	0	0
Cryptolithodes sp.	1	0	0	0	0	0	1	0	0	0	0	0	0	0	0	0	0	0	0	0
Oedignathus inermis	1	2	0	0	0	0	1	0	0	0	0	0	0	0	0	0	0	0	0	0
Hapalogaster mertensii	1	2	0	0	0	0	1	0	0	0	0	0	0	0	0	0	0	0	0	0
Pagurus bernhardus	1	0	0	0	0	1	1	0	0	1	0	0	0	0	0	0	0	0	0	0
Pagurus stimpsoni	1	0	0	0	0	1	1	0	0	1	0	0	0	0	0	0	0	0	0	0
Pagurus carolinensis	1	0	0	0	0	1	1	0	0	1	0	0	0	0	0	0	0	0	0	0
Pagurus brevidactylus	1	0	0	0	0	1	1	0	0	1	0	0	0	0	0	0	0	0	0	0
Pagurus maclaughlinae	1	0	0	0	0	1	1	0	0	1	0	0	0	0	0	0	0	0	0	0
Pagurus pollicaris	1	0	0	0	0	1	1	0	0	1	0	0	0	0	0	0	0	0	0	0
Pagurus bullisi	1	0	0	0	0	1	1	0	0	1	0	0	0	0	0	0	0	0	0	0
Iridopagurus caribbensis	1	0	0	0	0	1	1	0	0	1	0	0	0	0	0	0	0	0	0	0
Iridopagurus reticulatus	1	0	0	0	0	1	1	0	0	1	0	0	0	0	0	0	0	0	0	0
Xylopagurus cancellarius	1	0	0	0	0	1	1	0	1	1	1	0	0	0	0	0	0	0	0	0
Labidochirus splendescens	1	0	0	0	0	1	1	0	0	1	0	0	0	0	0	0	0	0	0	0
Porcellanopagurus filholi	1	0	0	0	0	1	1	0	0	1	0	0	0	0	0	0	0	0	0	0
Gorepagurus piercei	1	0	0	0	0	1	1	0	0	1	0	0	0	0	0	0	0	0	0	0
Manucomplanus ungulatus	1	0	0	0	0	1	1	0	0	1	0	0	0	0	0	0	0	0	0	0
Pylopagurus discoidalis	1	0	0	0	0	1	1	0	0	1	0	0	0	0	0	0	0	0	0	0
Pylopaguridium markhami	1	0	0	0	0	1	1	0	0	1	0	0	0	0	0	0	0	0	0	0
Phimochirus holthuisi	1	0	0	0	0	1	1	0	0	1	0	0	0	0	0	0	0	0	0	0
Phimochirus randalli	1	0	0	0	0	1	1	0	0	1	0	0	0	0	0	0	0	0	0	0
Phimochirus randalli	1	0	0	0	0	1	1	0	0	1	0	0	0	0	0	0	0	0	0	0
Agaricochirus alexandri	1	0	0	0	0	1	1	0	0	1	0	0	0	0	0	0	0	0	0	0
Tomopagurus merimaculosus	1	0	0	0	0	1	1	0	0	1	0	0	0	0	0	0	0	0	0	0
Discorsopagurus schmitti	1	0	0	0	0	1	1	0	0	1	0	0	0	0	0	0	0	0	0	0
Bythiopagurus macrocolus	1	0	0	0	0	1	1	0	0	1	0	0	0	0	0	0	0	0	0	0
Sympagurus dimorphus	1	0	0	0	0	1	1	0	0	1	0	0	1	0	0	0	0	0	0	0
Sympagurus acinops	1	0	0	0	0	1	1	0	0	1	0	0	1	0	0	0	0	0	0	0
Sympagurus pictus	1	0	0	0	0	1	1	0	0	1	0	0	1	0	0	0	0	0	0	0
Sympagurus sp.	1	0	0	0	0	1	1	0	0	1	0	0	1	0	0	0	0	0	0	0
Parapagurus latimanus	1	0	0	0	0	1	1	0	0	1	0	0	1	0	0	0	0	0	0	0
Trizocheles spinosus	1	0	0	0	0	1	1	0	1	1	0	0	0	1	1	0	0	0	0	0
Pomatocheles jeffreysii	1	0	0	0	0	0	1	0	1	?	0	0	0	1	0	0	0	0	0	0
Pachycheles haigae	1	0	0	0	0	0	1	0	0	1	0	0	0	1	0	1	1	2	0	0
Pachycheles rudis	1	0	0	0	0	0	1	0	0	1	0	0	0	1	0	1	1	2	0	0
Pachycheles rugimanus	1	0	0	0	0	0	1	0	0	1	0	0	0	1	0	1	1	2	0	0
Pachycheles ackleinus	1	0	0	0	0	0	1	0	0	1	0	0	0	1	0	1	1	2	0	0
Pachycheles pilosus	1	0	0	0	0	0	1	0	0	1	0	0	0	1	0	1	1	2	0	0
Polyonyx gibbesii	1	0	0	0	0	0	1	0	0	1	0	0	0	1	0	1	1	2	0	0
Pisidia magdalenensis	1	0	0	0	0	0	1	0	0	1	0	0	0	1	0	1	1	2	0	0
Megalobrachium poeyi	1	0	0	0	0	0	1	0	0	1	0	0	0	1	0	1	1	2	0	0
Petrolisthes armatus	1	0	0	0	0	0	1	0	0	1	0	0	0	1	0	1	1	2	0	0
Petrolisthes armatus	1	0	0	0	0	0	1	0	0	1	0	0	0	1	0	1	1	2	0	0
Petrolisthes laevigatus	1	0	0	0	0	0	1	0	0	1	0	0	0	1	0	1	1	2	0	0
Petrolisthes galathinus	1	0	0	0	0	0	1	0	0	1	0	0	0	1	0	1	1	2	0	0
Neopisosoma angustifrons	1	0	0	0	0	0	1	0	0	1	0	0	0	1	0	1	1	2	0	0
Parapetrolisthes tortugensis	1	0	0	0	0	0	1	0	0	1	0	0	0	1	0	1	1	2	0	0
Parapetrolisthes tortugensis	1	0	0	0	0	0	1	0	0	1	0	0	0	1	0	1	1	2	0	0
Allopetrolisthes spinifrons	1	0	0	0	0	0	1	0	0	1	0	0	0	1	0	1	1	2	0	0

Species	61	62	63	64	65	66	67	68	69	70	71	72	73	74	75	76	77	78	79	80
Porcellana sayana	1	0	0	0	0	0	1	0	0	1	0	0	0	1	0	1	1	2	0	0
Euceramus sp.	1	0	0	0	0	0	1	0	0	1	0	0	0	1	0	1	1	2	0	1
Munida subrugosa	1	0	0	0	0	0	1	0	0	1	0	0	0	1	0	0	0	0	0	0
Munida quadrispina	1	0	0	0	0	0	1	0	0	1	0	0	0	1	0	0	0	0	0	0
Munida iris	1	0	0	0	0	0	1	0	0	1	0	0	0	1	0	0	0	0	0	0
Munida pusilla	1	0	0	0	0	0	1	0	0	1	0	0	0	1	0	0	0	0	0	0
Pleuroncodes monodon	1	0	0	0	0	0	1	0	0	1	0	0	0	1	0	0	0	0	0	0
Cervimunida johni	1	0	0	0	0	0	1	0	0	1	0	0	0	1	0	0	0	0	0	0
Sadayoshia sp.	1	1	0	0	0	0	1	0	0	1	0	0	0	1	0	0	0	0	0	0
Babamunida kanaloa	1	0	0	0	0	0	1	0	0	1	0	0	0	1	0	0	0	0	0	0
Agononida procera	1	0	0	0	0	0	1	0	0	1	0	0	0	1	0	0	0	0	0	0
Neonida grandis	1	1	0	0	0	0	1	0	0	?	?	0	0	1	0	0	0	0	0	0
Anoplonida inermis	1	1	0	0	0	0	1	0	0	?	?	0	0	1	0	0	0	0	0	0
Bathymunida balssi	1	1	0	0	0	0	1	0	0	1	0	0	0	1	0	0	0	0	0	0
Alainius crosnieri	1	1	0	0	0	0	1	0	0	?	?	0	0	1	0	0	0	0	0	0
Galathea sp.	1	1	0	0	0	0	1	0	0	1	0	0	0	1	0	0	0	0	0	0
Galathea rostrata	1	1	0	0	0	0	1	0	0	1	0	0	0	1	0	0	0	0	0	0
Leiogalathea laevirostris	1	0	0	0	0	0	1	0	0	1	0	0	1	1	0	0	0	0	0	0
Shinkaia crosnieri	1	0	1	0	1	0	1	0	?	?	0	0	1	1	0	0	0	0	0	0
Munidopsis bairdii	1	0	1	0	0	0	1	0	0	1	0	0	1	1	0	0	0	0	0	0
Munidopsis erinacea	1	0	1	0	0	0	1	0	0	1	0	0	1	1	0	0	0	0	0	0
Pseudomunida fragilis	1	0	0	0	0	0	1	2	0	1	0	0	0	1	0	0	0	0	0	0
Galacantha rostrata	1	0	0	0	0	0	1	0	0	1	0	0	1	1	0	0	0	0	0	0
Galacantha valdiviae	1	0	0	0	0	0	1	0	0	1	0	0	1	1	0	0	0	0	0	0
Eumunida picta	1	0	0	0	0	0	1	2	0	1	0	0	0	1	0	0	0	0	0	0
Eumunida picta	1	0	0	0	0	0	1	2	0	1	0	0	0	1	0	0	0	0	0	0
Eumunida funambulus	1	0	0	0	0	0	1	2	0	1	0	0	0	1	0	0	0	0	0	0
Uroptychus spinirostris	1	0	0	0	0	0	1	1	0	1	0	0	0	0	0	0	0	0	0	0
Uroptychus nitidus	1	0	0	0	0	0	1	1	0	1	0	0	0	0	0	0	0	0	0	0
Uroptychus parvulus	1	0	0	0	0	0	1	1	0	1	0	0	0	0	0	0	0	0	0	0
Uroptychus scambus	1	0	0	0	0	0	1	1	0	1	0	0	0	0	0	0	0	0	0	0
Gastroptychus novaezelandiae	1	0	0	0	0	0	1	1	0	1	0	0	0	0	0	0	0	0	0	0
Gastroptychus rogeri	1	0	0	0	0	0	1	1	0	1	0	0	0	0	0	0	0	0	0	0
Gastroptychus spinifer	1	0	0	0	0	0	1	1	0	1	0	0	0	0	0	0	0	0	0	0
Chirostylus novaecaledoniae	1	0	0	0	0	0	1	1	0	1	0	0	1	0	0	0	0	0	0	0
Kiwa hirsuta	1	0	0	0	0	0	0	1	1	1	0	0	0	1	0	0	0	0	0	0
Aegla violacea	1	0	0	0	0	0	0	1	0	1	0	0	0	1	0	0	0	0	0	0
Aegla uruguyana	1	0	0	0	0	0	0	1	0	1	0	0	0	1	0	0	0	0	0	0
Aegla platensis	1	0	0	0	0	0	0	1	0	1	0	0	0	1	0	0	0	0	0	0
Aegla papudo	1	0	0	0	0	0	0	1	0	1	0	0	0	1	0	0	0	0	0	0
Aegla jarai	1	0	0	0	0	0	0	1	0	1	0	0	0	1	0	0	0	0	0	0
Aegla cholchol	1	0	0	0	0	0	0	1	0	1	0	0	0	1	0	0	0	0	0	0
Aegla camargoi	1	0	0	0	0	0	0	1	0	1	0	0	0	1	0	0	0	0	0	0
Aegla abtao	1	0	0	0	0	0	0	1	0	1	0	0	0	1	0	0	0	0	0	0
Aegla alacalufi	1	0	0	0	0	0	0	1	0	1	0	0	0	1	0	0	0	0	0	0
Lomis hirta	1	2	0	0	1	0	0	1	0	1	1	0	0	1	0	0	0	0	0	0

Species	81	82	83	84	85	86	87	88	89	90	91	92	93	94	95	96	97	98	99	100
Solenocera sp.	0	0	0	0	1	0	1	0	0	0	0	0	1	0	0	1	0	0	0	0
Hymenopenaeus debilis	0	0	0	0	1	0	1	0	0	0	0	0	1	0	0	1	0	0	0	0
Atyopsis sp.	0	0	0	0	1	0	1	0	0	0	0	0	1	0	0	1	0	0	0	0
Latreutes fucorum	0	0	0	0	1	0	1	0	0	0	0	0	1	0	0	1	0	0	0	0
Ogyrides sp.	0	0	0	0	1	0	1	0	0	0	0	0	1	0	0	1	0	0	0	0
Palaemonetes pugio	0	0	0	0	1	0	1	0	0	0	0	0	1	0	0	1	0	0	0	0
Calastacus crosnieri	0	0	0	0	1	0	1	0	0	0	0	0	1	0	1	1	0	0	0	1
Calaxius manningi	0	0	0	0	1	0	1	0	0	0	0	0	1	0	1	1	0	0	0	1
Lepidophthalmus louisianensis	0	1	0	0	1	0	1	0	0	0	0	0	1	0	1	0	-	0	0	1
Sergio mericeae	0	1	0	0	1	0	1	0	0	0	0	0	1	0	1	0	-	0	0	1
Austinogebia narutensis	0	1	0	0	1	0	1	0	0	0	0	0	1	0	1	0	-	0	0	1
Laemedia astacina	0	0	0	0	1	0	1	0	0	0	0	0	1	0	1	2	0	0	0	1
Thalassina anomala	0	0	0	0	1	0	1	0	0	0	0	0	1	0	1	2	0	1	0	1
Cosmonotus grayi	0	0	0	1	1	2	0	0	0	0	0	0	1	1	0	0	-	1	0	1
Calappa gallus	0	0	0	1	1	2	0	0	0	0	1	0	1	1	0	0	-	0	0	1
Chorilia longipes	0	0	0	1	1	2	0	0	0	0	1	0	1	1	0	0	-	0	0	0
Cyclograpsus cinereus	0	0	0	1	1	2	0	0	0	0	1	0	1	1	0	0	-	0	0	0
Praebebalia longidactyla	0	0	0	1	1	2	0	0	0	0	1	0	1	1	0	0	-	0	0	0
Blepharipoda occidentalis	0	1	0	0	0	1	1	0	0	0	0	1	1	0	0	2	0	0	0	0
Emerita emeritus	0	1	0	0	0	1	1	0	0	0	0	1	1	0	0	1	0	2	0	0
Emerita brasiliensis	0	1	0	0	0	1	1	0	0	0	0	1	1	0	0	1	0	2	0	0
Emerita talpoida	0	1	0	0	0	1	1	0	0	0	0	1	1	0	0	1	0	2	0	0
Albunea gibbesii	0	1	0	0	0	1	1	0	0	1	0	1	1	0	0	1	0	1	0	0
Albunea catherinae	0	1	0	0	0	1	1	0	0	1	0	1	1	0	0	1	0	1	0	0
Zygopa michaelis	0	1	0	0	0	1	1	0	0	0	0	1	1	0	0	1	0	1	0	0
Lepidopa californica	0	1	0	0	0	1	1	0	0	0	0	1	1	0	0	2	0	1	0	0
Lepidopa dexterae	0	1	0	0	0	1	1	0	0	0	0	1	1	0	0	2	0	1	0	0
Paraleucolepidopa	0	1	0	0	0	1	1	0	0	0	0	1	1	0	0	2	0	1	0	0
Coenobita compressus	0	1	0	0	1	2	0	0	1	0	0	1	1	0	0	0	-	0	1	0
Coenobita clypeatus	0	1	0	0	1	2	0	0	1	0	0	1	1	0	0	0	-	0	1	0
Coenobita perlatus	0	1	0	0	1	2	0	0	1	0	0	1	1	0	0	0	-	0	1	0
Birgus latro	0	1	0	0	1	2	0	1	1	0	0	1	1	0	0	1	0	0	1	0
Clibanarius albidigitus	0	1	0	1	1	2	0	0	0	0	0	1	1	0	0	1	-	0	0	0
Cilbanarius antillensis	0	1	0	1	1	2	0	0	0	0	0	1	1	0	0	1	-	0	0	0
Clibanarius corallinus	0	1	0	1	1	2	0	0	0	0	0	1	1	0	0	1	-	0	0	0
Clibanarius vittatus	0	1	0	1	1	2	0	0	0	0	0	1	1	0	0	1	-	0	0	0
Isocheles pilosus	0	1	0	1	1	2	0	1	0	0	0	1	1	0	0	1	0	0	0	0
Isocheles wurdmenni	0	1	0	1	1	2	0	1	0	0	0	1	1	0	0	1	0	0	0	0
Calcinus obscurus	0	1	0	1	1	2	0	1	0	0	0	1	1	0	0	1	0	0	1	0
Calcinus laevimanus	0	1	0	1	1	2	0	1	0	0	0	1	1	0	0	1	0	0	1	0
Paguristes turgidus	0	1	0	1	1	2	0	1	0	0	0	1	1	0	0	1	0	0	0	0
Paguristes tortugae	0	1	0	1	1	2	0	1	0	0	0	1	1	0	0	1	0	0	0	0
Paguristes triangulatus	0	1	0	1	1	2	0	1	0	0	0	1	1	0	0	1	0	0	0	0
Paguristes moorei	0	1	0	1	1	2	0	1	0	0	0	1	1	0	0	1	0	0	0	0
Paguristes sericeus	0	1	0	1	1	2	0	1	0	0	0	1	1	0	0	1	0	0	0	0
Paguristes grayi	0	1	0	1	1	2	0	1	0	0	0	1	1	0	0	1	0	0	0	0
Paguristes puncticeps	0	1	0	1	1	2	0	1	0	0	0	1	1	0	0	1	0	0	0	0
Paguristes cadenati	0	1	0	1	1	2	0	1	0	0	0	1	1	0	0	1	0	0	0	0
Areopaguristes hewatti	0	1	0	1	1	2	0	1	0	0	0	1	1	0	0	1	0	0	0	0
Areopaguristes hewatti	0	1	0	1	1	2	0	1	0	0	0	1	1	0	0	1	0	0	0	0
Areopaguristes hewatti	0	1	0	1	1	2	0	1	0	0	0	1	1	0	0	1	0	0	0	0
Areopaguristes pilosus	0	1	0	1	1	2	0	1	0	0	0	1	1	0	0	1	0	0	1	0
Areopaguristes hummi	0	1	0	1	1	2	0	1	0	0	0	1	1	0	0	1	0	0	0	0
Areopaguristes hummi	0	1	0	1	1	2	0	1	0	0	0	1	1	0	0	1	0	0	0	0
Dardanus fuscous	0	1	0	1	1	2	0	1	0	0	0	1	1	0	0	1	0	0	1	0
Dardanus insignis	0	1	0	1	1	2	0	1	0	0	0	1	1	0	0	1	0	0	1	0
Dardanus sp.	0	1	0	1	1	2	0	1	0	0	0	1	1	0	0	1	0	0	1	0
Petrochirus diogenes	0	1	0	1	1	2	0	1	0	0	0	1	1	0	0	1	0	0	0	0

Species	81	82	83	84	85	86	87	88	89	90	91	92	93	94	95	96	97	98	99	100
Lithodes santolla	1	1	0	1	1	2	0	1	0	0	0	1	1	0	0	1	0	0	2	0
Lithodes santolla	1	1	0	1	1	2	0	1	0	0	0	1	1	0	0	1	0	0	2	0
Glyptolithodes cristatipes	1	1	0	1	1	2	0	1	0	0	0	1	1	0	0	1	0	0	2	0
Paralomis sp.	1	1	0	1	1	2	0	1	0	0	0	1	1	0	0	1	0	0	2	0
Phyllolithodes papillosus	1	1	0	1	1	2	0	1	0	0	0	1	1	0	0	1	0	0	2	0
Lopholithodes mandtii	1	1	0	1	1	2	0	1	0	0	0	1	1	0	0	1	0	0	2	0
Paralithodes brevipes	1	1	0	1	1	2	0	1	0	0	0	1	1	0	0	1	0	0	2	0
Paralithodes camtschaticus	1	1	0	1	1	2	0	1	0	0	0	1	1	0	0	1	0	0	2	0
Paralithodes platypus	1	1	0	1	1	2	0	1	0	0	0	1	1	0	0	1	0	0	2	0
Cryptolithodes sp.	1	1	0	1	1	2	0	1	0	0	0	1	1	0	0	1	0	0	2	0
Oedignathus inermis	1	1	0	1	1	2	0	1	0	0	0	1	1	?	0	1	0	0	2	0
Hapalogaster mertensii	1	1	0	1	1	2	0	1	0	0	0	1	1	?	0	1	0	0	2	0
Pagurus bernhardus	1	1	0	1	1	2	0	1	0	0	0	1	1	0	0	1	0	0	2	0
Pagurus stimpsoni	1	1	0	1	1	2	0	1	0	0	0	1	1	0	0	1	0	0	2	0
Pagurus carolinensis	1	1	0	1	1	2	0	1	0	0	0	1	1	0	0	1	0	0	2	0
Pagurus brevidactylus	1	1	0	1	1	2	0	1	0	0	0	1	1	0	0	1	0	0	2	0
Pagurus maclaughlinae	1	1	0	1	1	2	0	1	0	0	0	1	1	0	0	1	0	0	2	0
Pagurus pollicaris	1	1	0	1	1	2	0	1	0	0	0	1	1	0	0	1	0	0	2	0
Pagurus bullisi	1	1	0	1	1	2	0	1	0	0	0	1	1	0	0	1	0	0	2	0
Iridopagurus caribbensis	0	1	0	1	1	2	0	1	0	0	0	1	1	0	0	1	0	0	2	0
Iridopagurus reticulatus	0	1	0	1	1	2	0	1	0	0	0	1	1	0	0	1	0	0	2	0
Xylopagurus cancellarius	1	1	0	1	1	2	0	1	0	0	0	1	1	0	0	1	0	0	2	0
Labidochirus splendescens	1	1	0	1	1	2	0	1	0	0	0	1	1	0	0	1	0	0	2	0
Porcellanopagurus filholi	1	1	0	1	1	2	0	1	0	0	0	1	1	0	0	1	0	0	2	0
Gorepagurus piercei	1	1	0	1	1	2	0	1	0	0	0	1	1	0	0	1	0	0	2	0
Manucomplanus ungulatus	1	1	0	1	1	2	0	1	0	0	0	1	1	0	0	1	0	0	2	0
Pylopagurus discoidalis	1	1	0	1	1	2	0	1	0	0	0	1	1	0	0	1	0	0	2	0
Pylopaguridium markhami	1	1	0	1	1	2	0	1	0	0	0	1	1	0	0	1	0	0	2	0
Phimochirus holthuisi	1	1	0	1	1	2	0	1	0	0	0	1	1	0	0	1	0	0	2	0
Phimochirus randalli	1	1	0	1	1	2	0	1	0	0	0	1	1	0	0	1	0	0	2	0
Phimochirus randalli	1	1	0	1	1	2	0	1	0	0	0	1	1	0	0	1	0	0	2	0
Agaricochirus alexandri	1	1	0	1	1	2	0	1	0	0	0	1	1	0	0	1	0	0	2	0
Tomopagurus merimaculosus	1	1	0	1	1	2	0	1	0	0	0	1	1	0	0	1	0	0	2	0
Discorsopagurus schmitti	1	1	0	1	1	2	0	1	0	0	0	1	1	0	0	1	0	0	2	0
Bythiopagurus macrocolus	1	1	0	1	1	2	0	1	0	0	0	1	1	0	0	1	0	0	2	0
Sympagurus dimorphus	0	1	0	1	1	2	0	0	0	0	0	1	1	0	0	1	0	0	2	0
Sympagurus acinops	0	1	0	1	1	2	0	0	0	0	0	1	1	0	0	1	0	0	2	0
Sympagurus pictus	0	1	0	1	1	2	0	0	0	0	0	1	1	0	0	1	0	0	2	0
Sympagurus sp.	0	1	0	1	1	2	0	0	0	0	0	1	1	0	0	1	0	0	2	0
Parapagurus latimanus	0	1	0	1	1	2	0	1	0	0	0	1	1	0	0	1	0	0	2	0
Trizocheles spinosus	1	1	0	1	1	2	0	1	0	0	0	1	1	0	0	1	0	0	0	0
Pomatocheles jeffreysii	0	0	0	0	1	2	0	0	0	0	0	1	1	0	0	1	0	0	0	0
Pachycheles haigae	0	1	1	1	0	2	0	0	0	0	0	1	0	1	0	0	-	0	1&2	0
Pachycheles rudis	0	1	1	1	0	2	0	0	0	0	0	1	0	1	0	0	-	0	1&2	0
Pachycheles rugimanus	0	1	1	1	0	2	0	0	0	0	0	1	0	1	0	0	-	0	1&2	0
Pachycheles ackleinus	0	1	1	1	0	2	0	0	0	0	0	1	0	1	0	0	-	0	1&2	0
Pachycheles pilosus	0	1	1	1	0	2	0	0	0	0	0	1	0	1	0	0	-	0	1&2	0
Polyonyx gibbesii	0	1	1	1	0	2	0	0	0	0	1	1	0	1	0	0	-	0	0&1	0
Pisidia magdalenensis	0	1	1	1	0	2	0	0	0	0	1	1	0	1	0	0	-	0	0&1	0
Megalobrachium poeyi	0	1	1	1	0	2	0	0	0	0	1	1	0	1	0	0	-	0	0&1	0
Petrolisthes armatus	0	1	1	1	0	2	0	0	0	0	0	1	0	1	0	0	-	0	0	1
Petrolisthes armatus	0	1	1	1	0	2	0	0	0	0	0	1	0	1	0	0	-	0	0	1
Petrolisthes laevigatus	0	1	1	1	0	2	0	0	0	0	0	1	0	1	0	0	-	0	0	1
Petrolisthes galathinus	0	1	1	1	0	2	0	0	0	0	0	1	0	1	0	0	-	0	0	1
Neopisosoma angustifrons	0	1	1	1	0	2	0	0	0	0	0	1	0	1	0	0	-	0	0	1
Parapetrolisthes tortugensis	0	1	1	1	0	2	0	0	0	0	0	1	0	1	0	0	-	0	0	1
Parapetrolisthes tortugensis	0	1	1	1	0	2	0	0	0	0	0	1	0	1	0	0	-	0	0	1
Allopetrolisthes spinifrons	0	1	1	1	0	2	0	0	0	0	0	1	0	1	0	0	-	0	0	1

Species	81	82	83	84	85	86	87	88	89	90	91	92	93	94	95	96	97	98	99	100
Porcellana sayana	0	1	1	1	0	2	0	0	0	0	1	1	0	1	0	0	-	0	0	1
Euceramus sp.	0	1	1	0	0	2	0	0	0	0	0	1	0	1	0	0	-	0	0	0
Munida subrugosa	0	0	1	1	1	2	0	0	0	0	0	1	0	0	0	0	-	0	0	0
Munida quadrispina	0	0	1	1	1	2	0	0	0	0	0	1	0	0	0	0	-	0	0	0
Munida iris	0	0	1	1	1	2	0	0	0	0	0	1	0	0	0	0	-	0	0	0
Munida pusilla	0	0	1	1	1	2	0	0	0	0	0	1	0	0	0	0	-	0	0	0
Pleuroncodes monodon	0	0	1	1	1	2	0	0	0	0	0	1	0	0	0	0	-	0	0	0
Cervimunida johni	0	0	1	1	1	2	0	0	0	0	0	1	0	0	0	0	-	0	0	0
Sadayoshia sp.	0	0	1	1	1	2	0	0	0	0	0	1	0	0	0	0	-	0	0	0
Babamunida kanaloa	0	0	1	1	1	2	0	0	0	0	0	1	0	0	0	0	-	0	0	0
Agononida procera	0	0	1	1	1	2	0	0	0	0	0	1	0	0	0	0	-	0	0	0
Neonida grandis	0	0	1	1	1	2	0	0	0	0	0	1	0	0	0	0	-	0	0	0
Anoplonida inermis	0	0	1	1	1	2	0	0	0	0	0	1	0	0	0	0	-	0	0	0
Bathymunida balssi	0	0	1	1	1	2	0	0	0	0	0	1	0	0	0	0	-	0	0	0
Alainius crosnieri	0	0	1	1	1	2	0	0	0	0	0	1	0	0	0	0	-	0	0	0
Galathea sp.	0	0	1	1	1	2	0	0	0	0	0	1	0	0	0	0	-	0	0	0
Galathea rostrata	0	0	1	1	1	2	0	0	0	0	0	1	0	0	0	0	-	0	0	0
Leiogalathea laevirostris	0	0	1	1	1	2	0	0	0	0	0	1	0	0	0	0	-	0	0	0
Shinkaia crosnieri	0	0	1	1	1	2	0	0	0	0	0	1	0	0	0	0	-	0	0	0
Munidopsis bairdii	0	0	1	1	1	2	0	0	0	0	0	1	0	0	0	0	-	0	0	0
Munidopsis erinacea	0	0	1	1	1	2	0	0	0	0	0	1	0	0	0	0	-	0	0	0
Pseudomunida fragilis	0	1	0	1	1	2	0	0	0	0	0	1	1	0	0	1	0	0	0	0
Galacantha rostrata	0	0	1	1	1	2	0	0	0	0	0	1	0	0	0	0	-	0	0	0
Galacantha valdiviae	0	0	1	1	1	2	0	0	0	0	0	1	0	0	0	0	-	0	0	0
Eumunida picta	0	1	0	1	1	2	0	0	0	0	0	1	1	0	0	1	0	0	0	0
Eumunida picta	0	1	0	1	1	2	0	0	0	0	0	1	1	0	0	1	0	0	0	0
Eumunida funambulus	0	1	0	1	1	2	0	0	0	0	0	1	1	0	0	1	0	0	0	0
Uroptychus spinirostris	0	1	0	1	1	2	0	0	0	0	0	1	1	0	0	1	0	0	0	0
Uroptychus nitidus	0	1	0	1	1	2	0	0	0	0	0	1	1	0	0	1	0	0	0	0
Uroptychus parvulus	0	1	0	1	1	2	0	0	0	0	0	1	1	0	0	1	0	0	0	0
Uroptychus scambus	0	1	0	1	1	2	0	0	0	0	0	1	1	0	0	1	0	0	0	0
Gastroptychus novaezelandiae	0	1	0	1	1	2	0	0	0	0	0	1	1	0	0	1	0	0	0	0
Gastroptychus rogeri	0	1	0	1	1	2	0	0	0	0	0	1	1	0	0	1	0	0	0	0
Gastroptychus spinifer	0	1	0	1	1	2	0	0	0	0	0	1	1	0	0	1	0	0	0	0
Chirostylus novaecaledoniae	0	1	0	1	1	2	0	0	0	0	0	1	1	0	0	0	-	0	0	0
Kiwa hirsuta	0	1	0	1	1	2	0	0	0	0	0	1	1	0	0	1	1	0	0	0
Aegla violacea	0	0	1	1	0	2	0	0	0	0	0	1	2	0	0	1	1	0	0	0
Aegla uruguyana	0	0	1	1	0	2	0	0	0	0	0	1	2	0	0	1	1	0	0	0
Aegla platensis	0	0	1	1	0	2	0	0	0	0	0	1	2	0	0	1	1	0	0	0
Aegla papudo	0	0	1	1	0	2	0	0	0	0	0	1	2	0	0	1	1	0	0	0
Aegla jarai	0	0	1	1	0	2	0	0	0	0	0	1	2	0	0	1	1	0	0	0
Aegla cholchol	0	0	1	1	0	2	0	0	0	0	0	1	2	0	0	1	1	0	0	0
Aegla camargoi	0	0	1	1	0	2	0	0	0	0	0	1	2	0	0	1	1	0	0	0
Aegla abtao	0	0	1	1	0	2	0	0	0	0	0	1	2	0	0	1	1	0	0	0
Aegla alacalufi	0	0	1	1	0	2	0	0	0	0	0	1	2	0	0	1	1	0	0	0
Lomis hirta	0	1	0	1	1	2	0	0	0	0	0	1	1	0	0	2	0	0	0	1

Species	101	102	103	104	105	106	107	108	109	110	111	112	113	114	115	116	117
Solenocera sp.	1	0	2	0	0	1	0	0	0	0	0	0	0	0	0	0	0
Hymenopenaeus debilis	1	0	2	0	0	1	0	0	0	0	0	0	0	0	0	0	0
Atyopsis sp.	0	0	2	0	0	1	1	0	0	0	0	0	0	2	0	0	0
Latreutes fucorum	0	0	2	0	0	1	1	0	0	0	0	0	0	0	0	0	0
Ogyrides sp.	0	0	2	0	0	1	1	0	0	0	0	0	0	0	0	0	0
Palaemonetes pugio	0	0	2	0	0	1	1	0	0	0	0	0	0	0	0	0	0
Calastacus crosnieri	1	0	2	1	1	1	1	0	0	0	0	0	0	0	0	0	1
Calaxius manningi	1	0	2	1	1	1	1	0	0	0	0	0	0	0	0	1	-
Lepidophthalmus louisianensis	1	0	2	1	1	1	1	0	0	0	0	0	0	0	0	0	1
Sergio mericeae	1	0	2	1	1	1	1	0	0	0	0	0	0	0	0	0	1
Austinogebia narutensis	1	0	1	1	1	1	1	0	0	0	0	0	0	0	0	1	-
Laemedia astacina	1	0	1	1	1	1	1	0	0	0	0	0	0	0	0	1	-
Thalassina anomala	1	0	0	1	1	1	1	0	0	0	0	0	0	0	0	0	1
Cosmonotus grayi	1	0	1	0	1	1	1	0	0	0	0	0	0	0	0	0	1
Calappa gallus	1	0	1	0	1	0	1	0	0	0	0	0	0	0	0	0	1
Chorilia longipes	1	0	1	0	1	0	1	0	0	0	0	0	0	0	0	0	1
Cyclograpsus cinereus	1	0	1	0	1	0	1	0	0	0	0	0	0	0	0	0	1
Praebebalia longidactyla	1	0	1	0	1	0	1	0	0	0	0	0	0	0	0	0	1
Blepharipoda occidentalis	1	0	1	1	1	1	1	0	0	0	0	0	1	1	0	1	-
Emerita emeritus	1	0	1	1	1	1	1	0	0	0	0	0	1	1	0	1	-
Emerita brasiliensis	1	1	1	1	1	1	1	0	0	0	0	0	1	1	0	1	-
Emerita talpoida	1	1	1	1	1	1	1	0	0	0	0	0	1	1	0	1	-
Albunea gibbesii	1	0	0	1	1	1	1	0	0	0	0	0	1	1	0	1	-
Albunea catherinae	1	0	0	1	1	1	1	0	0	0	0	0	1	1	0	1	-
Zygopa michaelis	1	0	0	1	1	1	1	0	0	0	0	0	1	1	0	1	-
Lepidopa californica	1	0	0	1	1	1	1	0	0	0	1	0	1	1	0	1	-
Lepidopa dexterae	1	0	0	1	1	1	1	0	0	0	1	0	1	1	0	1	-
Paraleucolepidopa	1	0	0	1	1	1	1	0	0	0	0	0	1	1	0	1	-
Coenobita compressus	1	0	1	0	0	0	1	1	1	0	0	1	1	1	-	1	-
Coenobita clypeatus	1	0	1	0	0	0	1	1	1	0	0	1	1	1	-	1	-
Coenobita perlatus	1	0	1	0	0	0	1	1	1	0	0	1	1	1	-	1	-
Birgus latro	0	0	1	0	0	0	1	2	1	0	0	0	1	1	-	1	-
Clibanarius albidigitus	0	0	1	0	0	0	1	1	1	0	0	1	1	1	1	1	-
Cilbanarius antillensis	0	0	1	0	0	0	1	1	1	0	0	1	1	1	1	1	-
Clibanarius corallinus	0	0	1	0	0	0	1	1	1	0	0	1	1	1	1	1	-
Clibanarius vittatus	0	0	1	0	0	0	1	1	1	0	0	1	1	1	1	1	-
Isocheles pilosus	0	0	1	0	0	0	1	1	1	0	0	1	1	1	1	1	-
Isocheles wurdmenni	0	0	1	0	0	0	1	1	1	0	0	1	1	1	1	1	-
Calcinus obscurus	0	0	1	0	0	0	1	1	1	0	0	1	1	1	1	1	-
Calcinus laevimanus	0	0	1	0	0	0	1	1	1	0	0	1	1	1	1	1	-
Paguristes turgidus	2	0	1	0	0	0	1	1	1	1	0	1	1	1	0	0	1
Paguristes tortugae	2	0	1	0	0	0	1	1	1	1	0	1	1	1	0	0	1
Paguristes triangulatus	2	0	1	0	0	0	1	1	1	1	0	1	1	1	0	0	1
Paguristes moorei	2	0	1	0	0	0	1	1	1	1	0	1	1	1	0	0	1
Paguristes sericeus	2	0	1	0	0	0	1	1	1	1	0	1	1	1	0	0	1
Paguristes grayi	2	0	1	0	0	0	1	1	1	1	0	1	1	1	0	0	1
Paguristes puncticeps	2	0	1	0	0	0	1	1	1	1	0	1	1	1	0	0	1
Paguristes cadenati	2	0	1	0	0	0	1	1	1	1	0	1	1	1	0	0	1
Areopaguristes hewatti	2	0	1	0	0	0	1	1	1	1	0	1	1	1	0	0	1
Areopaguristes hewatti	2	0	1	0	0	0	1	1	1	1	0	1	1	1	0	0	1
Areopaguristes hewatti	2	0	1	0	0	0	1	1	1	1	0	1	1	1	0	0	1
Areopaguristes pilosus	2	0	1	0	0	0	1	1	1	1	0	1	1	1	0	0	1
Areopaguristes hummi	2	0	1	0	0	0	1	1	1	1	0	1	1	1	0	0	1
Areopaguristes hummi	2	0	1	0	0	0	1	1	1	1	0	1	1	1	0	0	1
Dardanus fuscous	0	0	1	0	0	0	1	1	1	0	0	1	1	1	1	1	-
Dardanus insignis	0	0	1	0	0	0	1	1	1	0	0	1	1	1	1	1	-
Dardanus sp.	0	0	1	0	0	0	1	1	1	0	0	1	1	1	1	1	-
Petrochirus diogenes	0	0	1	0	0	0	1	1	1	0	0	1	1	1	1	1	-

Species	101	102	103	104	105	106	107	108	109	110	111	112	113	114	115	116	117
Lithodes santolla	0	0	1	0	0	0	1	0	0	0	0	0	1	1	1	1	-
Lithodes santolla	0	0	1	0	0	0	1	0	0	0	0	0	1	1	1	1	-
Glyptolithodes cristatipes	0	0	1	0	0	0	1	0	0	0	0	0	1	1	1	1	-
Paralomis sp.	0	0	1	0	0	0	1	0	0	0	0	0	1	1	1	1	-
Phyllolithodes papillosus	0	0	1	0	0	0	1	0	0	0	0	0	1	1	1	1	-
Lopholithodes mandtii	0	0	1	0	0	0	1	0	0	0	0	0	1	1	1	1	-
Paralithodes brevipes	0	0	1	0	0	0	1	0	0	0	0	0	1	1	1	1	-
Paralithodes camtschaticus	0	0	1	0	0	0	1	0	0	0	0	0	1	1	1	1	-
Paralithodes platypus	0	0	1	0	0	0	1	0	0	0	0	0	1	1	1	1	-
Cryptolithodes sp.	0	0	1	0	0	0	1	0	0	0	0	0	1	1	1	1	-
Oedignathus inermis	0	0	1	0	0	0	1	0	0	0	0	0	1	1	1	1	-
Hapalogaster mertensii	0	0	1	0	0	0	1	0	0	0	0	0	1	1	1	1	-
Pagurus bernhardus	2	0	1	0	0	0	1	1	1	0	0	1	1	1	1	1	-
Pagurus stimpsoni	2	0	1	0	0	0	1	1	1	0	0	1	1	1	1	1	-
Pagurus carolinensis	2	0	1	0	0	0	1	1	1	0	0	1	1	1	1	1	-
Pagurus brevidactylus	2	0	1	0	0	0	1	1	1	0	0	1	1	1	1	1	-
Pagurus maclaughlinae	2	0	1	0	0	0	1	1	1	0	0	1	1	1	1	1	-
Pagurus pollicaris	2	0	1	0	0	0	1	1	1	0	0	1	1	1	1	1	-
Pagurus bullisi	2	0	1	0	0	0	1	1	1	0	0	1	1	1	1	1	-
Iridopagurus caribbensis	0	0	1	0	0	0	1	0	1	0	0	1	1	1	1	1	-
Iridopagurus reticulatus	0	0	1	0	0	0	1	0	1	0	0	1	1	1	1	1	-
Xylopagurus cancellarius	1	0	1	0	0	0	1	1	1	0	0	1	1	1	0	0	1
Labidochirus splendescens	2	0	1	0	0	0	1	0	1	0	0	1	1	1	-	1	-
Porcellanopagurus filholi	1	0	1	0	0	0	1	1	1	0	0	1	1	1	1	1	-
Gorepagurus piercei	2	0	1	0	0	0	1	1	1	0	0	1	1	1	1	1	-
Manucomplanus ungulatus	2	0	1	0	0	0	1	1	1	2	0	1	1	1	1	1	-
Pylopagurus discoidalis	2	0	1	0	0	0	1	1	1	2	0	1	1	1	1	1	-
Pylopaguridium markhami	2	0	1	0	0	0	1	1	1	0	0	1	1	1	1	1	-
Phimochirus holthuisi	2	0	1	0	0	0	1	1	1	2	0	1	1	1	1	1	-
Phimochirus randalli	2	0	1	0	0	0	1	1	1	2	0	1	1	1	1	1	-
Phimochirus randalli	2	0	1	0	0	0	1	1	1	2	0	1	1	1	1	1	-
Agaricochirus alexandri	2	0	1	0	0	0	1	1	1	1	0	1	1	1	1	1	-
Tomopagurus merimaculosus	2	0	1	0	0	0	1	1	1	2	0	1	1	1	1	1	-
Discorsopagurus schmitti	0	0	1	0	0	0	1	1	1	0	0	1	1	1	1	1	-
Bythiopagurus macrocolus	1	0	1	0	0	0	1	1	1	0	0	1	1	1	0	0	1
Sympagurus dimorphus	2	0	1	0	0	0	1	1	1	0	0	1	1	1	0	0	1
Sympagurus acinops	2	0	1	0	0	0	1	1	1	0	0	1	1	1	0	0	1
Sympagurus pictus	2	0	1	0	0	0	1	1	1	0	0	1	1	1	0	0	1
Sympagurus sp.	2	0	1	0	0	0	1	1	1	0	0	1	1	1	0	0	1
Parapagurus latimanus	2	0	1	0	0	0	1	1	1	0	0	1	1	1	0	0	1
Trizocheles spinosus	0	0	1	0	0	0	1	1	1	0	0	1	1	1	0	0	1
Pomatocheles jeffreysii	?	0	1	0	0	0	1	0	1	0	0	1	1	1	0	0	1
Pachycheles haigae	1	0	1	0	0	0	1	0	0	0	0	0	1	1	0	1	-
Pachycheles rudis	1	0	1	0	0	0	1	0	0	0	0	0	1	1	0	1	-
Pachycheles rugimanus	1	0	1	0	0	0	1	0	0	0	0	0	1	1	0	1	-
Pachycheles ackleinus	1	0	1	0	0	0	1	0	0	0	0	0	1	1	0	1	-
Pachycheles pilosus	1	0	1	0	0	0	1	0	0	0	0	0	1	1	0	1	-
Polyonyx gibbesii	1	0	1	0	0	0	1	0	0	0	0	0	1	1	0	1	-
Pisidia magdalenensis	1	0	1	0	0	0	1	0	0	0	0	0	1	1	0	1	-
Megalobrachium poeyi	1	0	1	0	0	0	1	0	0	0	0	0	1	1	0	1	-
Petrolisthes armatus	1	0	1	0	0	0	1	0	0	0	0	0	1	1	0	1	-
Petrolisthes armatus	1	0	1	0	0	0	1	0	0	0	0	0	1	1	0	1	-
Petrolisthes laevigatus	1	0	1	0	0	0	1	0	0	0	0	0	1	1	0	1	-
Petrolisthes galathinus	1	0	1	0	0	0	1	0	0	0	0	0	1	1	0	1	-
Neopisosoma angustifrons	1	0	1	0	0	0	1	0	0	0	0	0	1	1	-	1	-
Parapetrolisthes tortugensis	1	0	1	0	0	0	1	0	0	0	0	0	1	1	0	1	-
Parapetrolisthes tortugensis	1	0	1	0	0	0	1	0	0	0	0	0	1	1	0	1	-
Allopetrolisthes spinifrons	1	0	1	0	0	0	1	0	0	0	0	0	1	1	0	1	-

Species	101	102	103	104	105	106	107	108	109	110	111	112	113	114	115	116	117
Porcellana sayana	1	0	1	0	0	0	1	0	0	0	0	0	1	1	0	1	-
Euceramus sp.	1	0	1	0	0	0	1	0	0	0	0	0	1	1	0	1	-
Munida subrugosa	1	0	1	0	0	0	1	0	0	0	0	0	1	1	0	0	1
Munida quadrispina	1	0	1	0	0	0	1	0	0	0	0	0	1	1	0	0	1
Munida iris	1	0	1	0	0	0	1	0	0	0	0	0	1	1	0	0	1
Munida pusilla	1	0	1	0	0	0	1	0	0	0	0	0	1	1	0	0	1
Pleuroncodes monodon	1	0	1	0	0	0	1	0	0	0	0	0	1	1	0	0	1
Cervimunida johni	1	0	1	0	0	0	1	0	0	0	0	0	1	1	0	0	1
Sadayoshia sp.	1	0	1	0	0	0	1	0	0	0	0	0	1	1	0	0	1
Babamunida kanaloa	1	0	1	0	0	0	1	0	0	0	0	0	1	1	0	0	1
Agononida procera	1	0	1	0	0	0	1	0	0	0	0	0	1	1	0	1	-
Neonida grandis	1	0	1	0	0	0	1	0	0	0	0	0	1	1	0	1	-
Anoplonida inermis	1	0	1	0	0	0	1	0	0	0	0	0	1	1	0	1	-
Bathymunida balssi	1	0	1	0	0	0	1	0	0	0	0	0	1	1	0	1	-
Alainius crosnieri	1	0	1	0	0	0	1	0	0	0	0	0	1	1	0	0	1
Galathea sp.	1	0	1	0	0	0	1	0	0	0	0	0	1	1	0	0	1
Galathea rostrata	1	0	1	0	0	0	1	0	0	0	0	0	1	1	0	0	1
Leiogalathea laevirostris	1	0	1	0	0	0	1	0	0	0	0	0	1	1	0	0	1
Shinkaia crosnieri	1	0	1	0	0	0	1	0	0	0	0	0	1	1	0	0	1
Munidopsis bairdii	1	0	1	0	0	0	1	0	0	0	0	0	1	1	0	0	1
Munidopsis erinacea	1	0	1	0	0	0	1	0	0	0	0	0	1	1	0	0	1
Pseudomunida fragilis	1	0	1	0	0	0	1	0	0	0	0	0	1	1	0	1	-
Galacantha rostrata	1	0	1	0	0	0	1	0	0	0	0	0	1	1	0	0	1
Galacantha valdiviae	1	0	1	0	0	0	1	0	0	0	0	0	1	1	0	0	1
Eumunida picta	1	0	1	0	0	0	1	0	0	0	0	0	1	1	0	1	-
Eumunida picta	1	0	1	0	0	0	1	0	0	0	0	0	1	1	0	1	-
Eumunida funambulus	1	0	1	0	0	0	1	0	0	0	0	0	1	1	0	1	-
Uroptychus spinirostris	1	0	1	0	0	0	1	0	0	0	0	0	1	1	0	0	1
Uroptychus nitidus	1	0	1	0	0	0	1	0	0	0	0	0	1	1	0	0	1
Uroptychus parvulus	1	0	1	0	0	0	1	0	0	0	0	0	1	1	0	0	1
Uroptychus scambus	1	0	1	0	0	0	1	0	0	0	0	0	1	1	0	0	1
Gastroptychus novaezelandiae	1	0	1	0	0	0	1	0	0	0	0	0	1	1	0	0	1
Gastroptychus rogeri	1	0	1	0	0	0	1	0	0	0	0	0	1	1	0	0	1
Gastroptychus spinifer	1	0	1	0	0	0	1	0	0	0	0	0	1	1	0	0	1
Chirostylus novaecaledoniae	1	0	1	0	0	0	1	0	0	0	0	0	1	1	0	0	1
Kiwa hirsuta	1	0	1	0	0	0	1	0	0	0	0	0	1	1	0	1	-
Aegla violacea	0	0	1	0	0	0	1	0	0	0	0	0	1	1	0	1	-
Aegla uruguyana	0	0	1	0	0	0	1	0	0	0	0	0	1	1	0	1	-
Aegla platensis	0	0	1	0	0	0	1	0	0	0	0	0	1	1	0	1	-
Aegla papudo	0	0	1	0	0	0	1	0	0	0	0	0	1	1	0	1	-
Aegla jarai	0	0	1	0	0	0	1	0	0	0	0	0	1	1	0	1	-
Aegla cholchol	0	0	1	0	0	0	1	0	0	0	0	0	1	1	0	1	-
Aegla camargoi	0	0	1	0	0	0	1	0	0	0	0	0	1	1	0	1	-
Aegla abtao	0	0	1	0	0	0	1	0	0	0	0	0	1	1	0	1	-
Aegla alacalufi	0	0	1	0	0	0	1	0	0	0	0	0	1	1	0	1	-
Lomis hirta	1	0	1	0	0	0	1	0	0	0	0	0	1	1	0	0	1

Species	118	119	120	121	122	123	124	125	126	127	128	129	130	131	132	133	134
Solenocera sp.	0	0	0	0	0	0	0	0	0	0	0	0	?	?	?	?	?
Hymenopenaeus debilis	0	0	0	0	0	0	0	0	0	0	0	0	?	?	?	?	?
Atyopsis sp.	0	0	0	0	0	0	0	0	0	0	1	1	?	?	?	?	?
Latreutes fucorum	0	0	0	0	0	0	0	0	0	0	0	1	?	?	?	?	?
Ogyrides sp.	0	0	0	0	0	0	0	0	0	0	0	1	?	?	?	?	?
Palaemonetes pugio	0	0	0	0	0	0	0	0	0	0	0	1	?	?	?	?	?
Calastacus crosnieri	0	0	0	1	0	0	0	0	0	0	0	1	?	?	?	?	?
Calaxius manningi	0	0	0	1	0	0	0	0	0	0	0	1	?	?	?	?	?
Lepidophthalmus louisianensis	0	0	0	1	0	0	0	0	0	0	0	1	?	?	?	?	?
Sergio mericeae	0	0	0	1	0	0	0	0	0	0	0	1	?	?	?	?	?
Austinogebia narutensis	0	0	0	1	0	0	0	0	0	0	0	1	?	?	?	?	?
Laemedia astacina	0	0	0	1	0	0	0	0	0	0	0	1	?	?	?	?	?
Thalassina anomala	0	0	0	1	0	0	0	0	0	0	0	1	2	?	0	0	?
Cosmonotus grayi	0	1	0	0	0	0	1	0	0	0	0	1	2	0	?	2	0
Calappa gallus	0	1	0	0	0	0	1	0	0	0	0	1	2	0	?	2	0
Chorilia longipes	0	1	0	0	0	0	1	0	0	0	0	1	2	0	?	2	0
Cyclograpsus cinereus	0	1	0	0	0	0	1	0	0	0	0	1	2	0	?	2	0
Praebebalia longidactyla	0	1	0	0	0	0	1	0	0	0	0	1	?	?	?	?	?
Blepharipoda occidentalis	1	1	0	0	0	0	1	0	0	0	0	1	?	0	?	?	?
Emerita emeritus	1	1	0	0	0	0	1	0	0	0	0	1	1	0	0	1	0&1
Emerita brasiliensis	1	1	0	0	0	0	1	0	0	0	0	1	1	0	0	1	0&1
Emerita talpoida	1	1	0	0	0	0	1	0	0	0	0	1	1	0	0	1	0&1
Albunea gibbesii	1	1	0	0	0	0	1	0	0	0	0	1	?	?	?	?	?
Albunea catherinae	1	1	0	0	0	0	1	0	0	0	0	1	?	?	?	?	?
Zygopa michaelis	1	1	0	0	0	0	1	0	0	0	0	1	?	?	?	?	?
Lepidopa californica	1	1	0	0	0	0	1	0	0	0	0	1	?	?	?	?	?
Lepidopa dexterae	1	1	0	0	0	0	1	0	0	0	0	1	?	?	?	?	?
Paraleucolepidopa	1	1	0	0	0	0	1	0	0	0	0	1	?	?	?	?	?
Coenobita compressus	1	1	0	0	0	2	0	1	1	2	0	1	1	0	1	1	0
Coenobita clypeatus	1	1	0	0	0	2	0	1	1	2	0	1	1	0	1	1	0
Coenobita perlatus	1	1	0	0	0	2	0	1	1	2	0	1	1	0	1	1	0
Birgus latro	1	1	0	0	0	2	1	1	1	2	0	1	1	0	1	1	0
Clibanarius albidigitus	0	0	0	0	0	1	0	1	1	2	0	1	0	0	0	1	0
Cilbanarius antillensis	0	0	0	0	0	1	0	1	1	2	0	1	0	0	0	1	0
Clibanarius corallinus	0	0	0	0	0	1	0	1	1	2	0	1	0	0	0	1	0
Clibanarius vittatus	0	0	0	0	0	1	0	1	1	2	0	1	0	0	0	1	0
Isocheles pilosus	0	0	0	?	?	?	0	1	1	1	0	1	0	0	?	1	0
Isocheles wurdmenni	0	0	0	?	?	?	0	1	1	1	0	1	0	0	?	1	0
Calcinus obscurus	0	0	0	0	0	2	0	1	1	1	0	1	0	0	0	1	0
Calcinus laevimanus	0	0	0	0	0	2	0	1	1	1	0	1	0	0	0	1	0
Paguristes turgidus	0	0	0	1	0	2	0	1	1	1	0	1	0	0	?	1	0
Paguristes tortugae	0	0	0	1	0	2	0	1	1	1	0	1	0	0	?	1	0
Paguristes triangulatus	0	0	0	1	0	2	0	1	1	1	0	1	0	0	?	1	0
Paguristes moorei	0	0	0	1	0	2	0	1	1	1	0	1	0	0	?	1	0
Paguristes sericeus	0	0	0	1	0	2	0	1	1	1	0	1	0	0	?	1	0
Paguristes grayi	0	0	0	1	0	2	0	1	1	1	0	1	0	0	?	1	0
Paguristes puncticeps	0	0	0	1	0	2	0	1	1	1	0	1	0	0	?	1	0
Paguristes cadenati	0	0	0	1	0	2	0	1	1	1	0	1	0	0	?	1	0
Areopaguristes hewatti	0	0	0	1	0	2	0	1	1	1	0	1	0	0	?	1	0
Areopaguristes hewatti	0	0	0	1	0	2	0	1	1	1	0	1	0	0	?	1	0
Areopaguristes hewatti	0	0	0	1	0	2	0	1	1	1	0	1	0	0	?	1	0
Areopaguristes pilosus	0	0	0	1	0	2	0	1	1	1	0	1	0	0	?	1	0
Areopaguristes hummi	0	0	0	1	0	2	0	1	1	1	0	1	0	0	?	1	0
Areopaguristes hummi	0	0	0	1	0	2	0	1	1	1	0	1	0	0	?	1	0
Dardanus fuscous	0	0	0	0	0	2	?	1	1	1	0	1	0	0	1	1	0
Dardanus insignis	0	0	0	0	0	2	?	1	1	1	0	1	0	0	1	1	0
Dardanus sp.	0	0	0	0	0	2	?	1	1	1	0	1	0	0	1	1	0
Petrochirus diogenes	0	0	0	0	0	2	?	1	1	1	0	1	0	0	?	1	0

**Species**	**118**	**119**	**120**	**121**	**122**	**123**	**124**	**125**	**126**	**127**	**128**	**129**	**130**	**131**	**132**	**133**	**134**
Lithodes santolla	1	1	0	0	0	2	1	1	1	1	0	1	0	0	?	1	0
Lithodes santolla	1	1	0	0	0	2	1	1	1	1	0	1	0	0	?	1	0
Glyptolithodes cristatipes	1	1	0	0	0	2	1	1	1	1	0	1	0	0	?	1	0
Paralomis sp.	1	1	0	0	0	2	1	1	1	1	0	1	0	0	?	1	0
Phyllolithodes papillosus	1	1	0	0	0	2	1	1	1	1	0	1	0	0	?	1	0
Lopholithodes mandtii	1	1	0	0	0	2	1	1	1	1	0	1	0	0	?	1	0
Paralithodes brevipes	1	1	0	0	0	2	1	1	1	1	0	1	0	0	?	1	0
Paralithodes camtschaticus	1	1	0	0	0	2	1	1	1	1	0	1	0	0	?	1	0
Paralithodes platypus	1	1	0	0	0	2	1	1	1	1	0	1	0	0	?	1	0
Cryptolithodes sp.	1	1	0	0	0	2	1	1	1	1	0	1	0	0	?	1	0
Oedignathus inermis	1	1	0	0	0	2	1	1	1	1	0	1	0	0	?	1	0
Hapalogaster mertensii	1	1	0	0	0	2	1	1	1	1	0	1	0	0	?	1	0
Pagurus bernhardus	1	0	0	1	0	2	1	1	1	1	0	1	0	0	0	1	0
Pagurus stimpsoni	1	0	0	1	0	2	1	1	1	1	0	1	0	0	0	1	0
Pagurus carolinensis	1	0	0	1	0	2	1	1	1	1	0	1	0	0	0	1	0
Pagurus brevidactylus	1	0	0	1	0	2	1	1	1	1	0	1	0	0	0	1	0
Pagurus maclaughlinae	1	0	0	1	0	2	1	1	1	1	0	1	0	0	0	1	0
Pagurus pollicaris	1	0	0	1	0	2	1	1	1	1	0	1	0	0	0	1	0
Pagurus bullisi	1	0	0	1	0	2	1	1	1	1	0	1	0	0	0	1	0
Iridopagurus caribbensis	1	0	1	0	0	2	0	1	1	1	0	1	?	?	?	?	?
Iridopagurus reticulatus	1	0	1	0	0	2	0	1	1	1	0	1	?	?	?	?	?
Xylopagurus cancellarius	0	1	0	0	0	2	0	1	1	2	0	1	0	0	?	1	?
Labidochirus splendescens	1	1	0	0	0	2	0	1	1	1	0	1	?	?	?	?	?
Porcellanopagurus filholi	1	1	1	0	0	2	0	1	1	2	0	1	1	0	?	1	0
Gorepagurus piercei	1	0	1	1	0	2	0	1	1	1	0	1	?	?	?	?	?
Manucomplanus ungulatus	1	0	0	1	0	2	0	1	1	1	0	1	?	?	?	?	?
Pylopagurus discoidalis	1	0	1	1	0	2	0	1	1	1	0	1	?	?	?	?	?
Pylopaguridium markhami	1	0	0	1	0	2	0	1	1	1	0	1	?	?	?	?	?
Phimochirus holthuisi	1	0	0	1	0	2	0	1	1	1	0	1	?	?	?	?	?
Phimochirus randalli	1	0	0	1	0	2	0	1	1	1	0	1	?	?	?	?	?
Phimochirus randalli	1	0	0	1	0	2	0	1	1	1	0	1	?	?	?	?	?
Agaricochirus alexandri	1	0	1	1	0	2	0	1	1	1	0	1	?	?	?	?	?
Tomopagurus merimaculosus	1	0	0	1	0	2	0	1	1	1	0	1	?	?	?	?	?
Discorsopagurus schmitti	1	0	0	1	0	2	1	1	1	1	0	1	1	0	?	1	0
Bythiopagurus macrocolus	1	0	1	0	0	1	1	1	1	2	0	1	1	0	?	1	0
Sympagurus dimorphus	0	0	0	1	0	2	1	1	1	2	0	1	1	0	0	1	0
Sympagurus acinops	0	0	0	1	0	2	1	1	1	2	0	1	1	0	0	1	0
Sympagurus pictus	0	0	0	1	0	2	1	1	1	2	0	1	1	0	0	1	0
Sympagurus sp.	0	0	0	1	0	2	1	1	1	2	0	1	1	0	0	1	0
Parapagurus latimanus	0	0	0	1	0	1	1	1	1	0	0	1	1	0	?	1	0
Trizocheles spinosus	0	0	0	0	0	0	1	0	0	0	0	1	2	0	?	1	0
Pomatocheles jeffreysii	0	0	0	0	0	0	1	0	0	0	0	1	2	0	?	1	0
Pachycheles haigae	1	1	0	0	?	?	?	?	0	0	0	1	0	0	?	1	1
Pachycheles rudis	1	1	0	0	?	?	?	?	0	0	0	1	0	0	?	1	1
Pachycheles rugimanus	1	1	0	0	?	?	?	?	0	0	0	1	0	0	?	1	1
Pachycheles ackleinus	1	1	0	0	?	?	?	?	0	0	0	1	0	0	?	1	1
Pachycheles pilosus	1	1	0	0	?	?	?	?	0	0	0	1	0	0	?	1	1
Polyonyx gibbesii	0	1	0	0	1	-	1	?	?	?	0	1	0	0	0	1	1
Pisidia magdalenensis	0	1	0	0	1	-	1	?	?	?	0	1	0	0	0	1	1
Megalobrachium poeyi	0	1	0	0	1	-	1	?	?	?	0	1	?	?	?	?	?
Petrolisthes armatus	0	1	0	0	1	-	1	2	0	0	0	1	0	0	0	1	1
Petrolisthes armatus	0	1	0	0	1	-	1	2	0	0	0	1	0	0	0	1	1
Petrolisthes laevigatus	0	1	0	0	1	-	1	2	0	0	0	1	0	0	0	1	1
Petrolisthes galathinus	0	1	0	0	1	-	1	2	0	0	0	1	0	0	0	1	1
Neopisosoma angustifrons	1	1	0	0	1	-	1	2	0	0	0	1	?	?	?	?	?
Parapetrolisthes tortugensis	0	1	0	0	1	-	1	2	0	0	0	1	?	?	?	?	?
Parapetrolisthes tortugensis	0	1	0	0	1	-	1	2	0	0	0	1	?	?	?	?	?
Allopetrolisthes spinifrons	0	1	0	0	1	-	1	2	0	0	0	1	?	?	?	?	?

Species	118	119	120	121	122	123	124	125	126	127	128	129	130	131	132	133	134
Porcellana sayana	0	1	0	0	1	-	1	?	?	?	0	1	0	0	?	1	1
Euceramus sp.	0	?	0	0	1	-	1	0	0	0	0	1	0	0	?	1	1
Munida subrugosa	0	0	0	0	0	0	1	0	0	0	0	1	0	0	0	1	0
Munida quadrispina	0	0	0	0	0	0	1	0	0	0	0	1	0	0	0	1	0
Munida iris	0	0	0	0	0	0	1	0	0	0	0	1	0	0	0	1	0
Munida pusilla	0	0	0	0	0	0	1	0	0	0	0	1	0	0	0	1	0
Pleuroncodes monodon	0	0	0	0	0	0	1	0	0	0	0	1	0	0	?	1	0
Cervimunida johni	0	0	0	0	0	0	1	0	0	0	0	1	0	0	?	1	0
Sadayoshia sp.	0	0	0	0	0	0	1	0	0	0	0	1	0	0	?	1	0
Babamunida kanaloa	0	0	0	0	0	0	1	0	0	0	0	1	0	0	?	1	0
Agononida procera	0	0	0	0	0	0	1	0	0	0	0	1	0	0	?	1	0
Neonida grandis	0	0	0	0	0	0	1	0	0	0	0	1	0	0	?	1	0
Anoplonida inermis	0	0	0	0	0	0	1	0	0	0	0	1	0	0	?	1	0
Bathymunida balssi	0	0	0	0	0	0	1	0	0	0	0	1	0	0	?	1	0
Alainius crosnieri	0	0	0	0	0	0	1	0	0	0	0	1	0	0	?	1	0
Galathea sp.	0	0	0	0	0	0	1	0	0	0	0	1	0	0	?	1	0
Galathea rostrata	0	0	0	0	0	0	1	0	0	0	0	1	0	0	0	1	0
Leiogalathea laevirostris	0	0	0	0	0	0	1	0	0	0	0	1	0	0	?	1	0
Shinkaia crosnieri	0	0	0	0	0	0	1	0	0	0	0	1	?	?	?	?	?
Munidopsis bairdii	0	0	0	0	0	0	1	0	0	0	0	1	0	0	0	1	0
Munidopsis erinacea	0	0	0	0	0	0	1	0	0	0	0	1	0	0	0	1	0
Pseudomunida fragilis	1	1	0	0	0	0	1	0	0	0	0	1	1	0	?	1	?
Galacantha rostrata	0	0	0	0	0	0	1	0	0	0	0	1	0	0	?	1	0
Galacantha valdiviae	0	0	0	0	0	0	1	0	0	0	0	1	0	0	?	1	0
Eumunida picta	1	1	0	0	0	0	1	0	0	0	0	1	1	0	1	1	?
Eumunida picta	1	1	0	0	0	0	1	0	0	0	0	1	1	0	1	1	?
Eumunida funambulus	1	1	0	0	0	0	1	0	0	0	0	1	1	0	1	1	?
Uroptychus spinirostris	0	1	0	0	1	?	1	0	0	2	0	1	0	0	1	1	0
Uroptychus nitidus	0	1	0	0	?	?	1	0	0	?	0	1	0	0	1	1	0
Uroptychus parvulus	0	1	0	0	1	-	1	0	0	2	0	1	0	0	1	1	0
Uroptychus scambus	0	1	0	0	0	0	1	0	0	2	0	1	0	0	1	1	0
Gastroptychus novaezelandiae	0	1	0	0	1	-	1	0	0	2	0	1	?	0	?	1	0
Gastroptychus rogeri	0	1	0	0	0	0	1	0	0	0	0	1	?	0	?	1	0
Gastroptychus spinifer	0	1	0	0	0	0	1	0	0	0	0	1	?	0	?	1	0
Chirostylus novaecaledoniae	0	1	0	0	?	?	1	0	0	?	0	1	?	0	?	1	0
Kiwa hirsuta	0	0	0	0	0	0	1	0	0	0	?	1	?	?	?	?	?
Aegla violacea	1	1	0	0	0	0	1	0	0	0	1	1	0	1	?	1	2
Aegla uruguyana	1	1	0	0	0	0	1	0	0	0	1	1	0	1	?	1	2
Aegla platensis	1	1	0	0	0	0	1	0	0	0	1	1	0	1	?	1	2
Aegla papudo	1	1	0	0	0	0	1	0	0	0	1	1	0	1	?	1	2
Aegla jarai	1	1	0	0	0	0	1	0	0	0	1	1	0	1	?	1	2
Aegla cholchol	1	1	0	0	0	0	1	0	0	0	1	1	0	1	?	1	2
Aegla camargoi	1	1	0	0	0	0	1	0	0	0	1	1	0	1	?	1	2
Aegla abtao	1	1	0	0	0	0	1	0	0	0	1	1	0	1	?	1	2
Aegla alacalufi	1	1	0	0	0	0	1	0	0	0	1	1	0	1	?	1	2
Lomis hirta	0	1	0	0	0	0	0	0	0	0	0	1	0	1	0	1	2

Species	135	136	137	138	139	140	141	142	143	144	145	146	147	148	149	150	151
Solenocera sp.	?	?	?	?	?	?	?	?	?	?	?	0	?	?	?	?	?
Hymenopenaeus debilis	?	?	?	?	?	?	?	?	?	?	?	0	?	?	?	?	?
Atyopsis sp.	?	?	?	?	?	?	?	?	?	-	-	?	-	-	-	-	-
Latreutes fucorum	?	?	?	?	?	?	?	?	?	0	0	0	1	1	0	0	?
Ogyrides sp.	?	?	?	?	?	?	?	?	?	0	0	0	1	1	0	0	?
Palaemonetes pugio	?	?	?	?	?	?	?	?	?	0	0	0	1	1	0	0	?
Calastacus crosnieri	?	?	?	?	?	?	?	?	?	?	?	?	?	?	?	?	?
Calaxius manningi	?	?	?	?	?	?	?	?	?	?	?	?	?	?	?	?	?
Lepidophthalmus louisianensis	?	?	?	?	2	?	?	?	?	0	0	0	0	1	0	0	0
Sergio mericeae	?	?	?	?	2	?	?	?	?	0	0	0	0	1	2	0	?
Austinogebia narutensis	?	?	?	?	?	?	?	?	?	0	0	0	0	1	3	0	?
Laemedia astacina	?	?	?	?	?	?	?	?	?	0	0	1	0	1	2	0	?
Thalassina anomala	0	0	?	0	?	?	?	?	?	?	?	1	?	?	?	?	?
Cosmonotus grayi	0	0	1	0	1	0	0	?	?	?	?	0	?	?	?	?	?
Calappa gallus	0	0	1	0	1	0	0	0	?	0	1	0	0	1	0	0	2
Chorilia longipes	0	0	1	0	1	0	0	0	?	0	1	0	0	1	?	?	?
Cyclograpsus cinereus	0	0	1	0	1	0	0	0	?	0	0	0	0	1	0	0	2
Praebebalia longidactyla	?	?	?	?	?	?	?	?	?	?	?	?	?	?	?	?	?
Blepharipoda occidentalis	?	?	?	?	?	?	?	0	?	0	0	0	0	0	0	0	?
Emerita emeritus	0	0	0	0	0	0	0	0	0	0	0	0	0	1	0	0	?
Emerita brasiliensis	0	0	0	0	0	0	0	0	0	0	0	0	0	1	0	0	?
Emerita talpoida	0	0	0	0	0	0	0	0	0	0	0	0	0	1	0	0	?
Albunea gibbesii	?	?	?	?	?	?	?	?	?	1	0	0	0	1	0	0	?
Albunea catherinae	?	?	?	?	?	?	?	?	?	1	0	0	0	1	0	0	?
Zygopa michaelis	?	?	?	?	?	?	?	?	?	?	?	0	?	?	?	?	?
Lepidopa californica	?	?	?	?	?	?	?	?	?	1	0	0	0	1	0	0	?
Lepidopa dexterae	?	?	?	?	?	?	?	?	?	1	0	0	0	1	0	0	?
Paraleucolepidopa	?	?	?	?	?	?	?	?	?	1	0	0	0	1	0	0	?
Coenobita compressus	0	1	0	0	1	0	1	0	2	0	0	0	0	1	2	0	1
Coenobita clypeatus	0	1	0	0	1	0	1	0	2	0	0	0	0	1	2	0	1
Coenobita perlatus	0	1	0	0	1	0	1	0	2	0	0	0	0	1	2	0	1
Birgus latro	0	1	0	0	1	0	1	0	2	0	0	0	0	1	2	0	1
Clibanarius albidigitus	0	1	0	0	1	0	1	0	1&3	0	0	0	0	1	2	0	1
Cilbanarius antillensis	0	1	0	0	1	0	1	0	1&3	0	0	0	0	1	2	0	1
Clibanarius corallinus	0	1	0	0	1	0	1	0	1&3	0	0	0	0	1	2	0	1
Clibanarius vittatus	0	1	0	0	1	0	1	0	1&3	0	0	0	0	1	2	0	1
Isocheles pilosus	0	1	0	0	1	0	1	0	?	0	0	0	0	1	1	0	1
Isocheles wurdmenni	0	1	0	0	1	0	1	0	?	0	0	0	0	1	1	0	1
Calcinus obscurus	0	1	0	0	1	0	1	0	3	1	0	0	0	1	2	0	?
Calcinus laevimanus	0	1	0	0	1	0	1	0	3	1	0	0	0	1	2	0	?
Paguristes turgidus	0	1	0	0	1	0	1	0	?	0	0	0	0	1	2	0	1
Paguristes tortugae	0	1	0	0	1	0	1	0	?	0	0	0	0	1	2	0	1
Paguristes triangulatus	0	1	0	0	1	0	1	0	?	0	0	0	0	1	2	0	1
Paguristes moorei	0	1	0	0	1	0	1	0	?	0	0	0	0	1	2	0	1
Paguristes sericeus	0	1	0	0	1	0	1	0	?	0	0	0	0	1	2	0	1
Paguristes grayi	0	1	0	0	1	0	1	0	?	0	0	0	0	1	2	0	1
Paguristes puncticeps	0	1	0	0	1	0	1	0	?	0	0	0	0	1	2	0	1
Paguristes cadenati	0	1	0	0	1	0	1	0	?	0	0	0	0	1	2	0	1
Areopaguristes hewatti	0	1	0	0	1	0	1	0	?	0	0	0	0	1	2	0	1
Areopaguristes hewatti	0	1	0	0	1	0	1	0	?	0	0	0	0	1	2	0	1
Areopaguristes hewatti	0	1	0	0	1	0	1	0	?	0	0	0	0	1	2	0	1
Areopaguristes pilosus	0	1	0	0	1	0	1	0	?	0	0	0	0	1	2	0	1
Areopaguristes hummi	0	1	0	0	1	0	1	0	?	0	0	0	0	1	2	0	1
Areopaguristes hummi	0	1	0	0	1	0	1	0	?	0	0	0	0	1	2	0	1
Dardanus fuscous	0	1	0	0	1	0	1	0	3	0	0	0	0	1	2	0	?
Dardanus insignis	0	1	0	0	1	0	1	0	3	0	0	0	0	1	2	0	?
Dardanus sp.	0	1	0	0	1	0	1	0	3	0	0	0	0	1	2	0	?
Petrochirus diogenes	0	1	0	0	1	0	1	0	3	0	0	0	0	1	2	0	?

Species	135	136	137	138	139	140	141	142	143	144	145	146	147	148	149	150	151
Lithodes santolla	0	0	1	0	1	0	1	1	2&3	1	0	0	0	1	0	1	1
Lithodes santolla	0	0	1	0	1	0	1	1	2&3	1	0	0	0	1	0	1	1
Glyptolithodes cristatipes	0	0	1	0	1	0	1	1	2&3	1	0	0	0	1	0	1	1
Paralomis sp.	0	0	1	0	1	0	1	1	2&3	1	0	0	0	1	0	1	1
Phyllolithodes papillosus	0	0	1	0	1	0	1	1	2&3	?	?	0	?	?	?	?	?
Lopholithodes mandtii	0	0	1	0	1	0	1	1	2&3	1	0	0	0	1	0	1	1
Paralithodes brevipes	0	0	1	0	1	0	1	1	2&3	1	0	0	0	1	0	1	1
Paralithodes camtschaticus	0	0	1	0	1	0	1	1	2&3	1	0	0	0	1	0	1	1
Paralithodes platypus	0	0	1	0	1	0	1	1	2&3	1	0	0	0	1	0	1	1
Cryptolithodes sp.	0	0	1	0	1	0	1	1	2&3	1	0	0	0	1	0	1	1
Oedignathus inermis	0	0	1	0	1	0	1	1	?	?	?	?	?	?	?	?	?
Hapalogaster mertensii	0	0	1	0	1	0	1	1	?	1	0	0	0	1	0	0	1
Pagurus bernhardus	1	0	0	1	1	0	1	1	1&3	1	0	0	0	1	0	0	1
Pagurus stimpsoni	1	0	0	1	1	0	1	1	1&3	1	0	0	0	1	0	0	1
Pagurus carolinensis	1	0	0	1	1	0	1	1	1&3	1	0	0	0	1	0	0	1
Pagurus brevidactylus	1	0	0	1	1	0	1	1	1&3	1	0	0	0	1	0	0	1
Pagurus maclaughlinae	1	0	0	1	1	0	1	1	1&3	1	0	0	0	1	1	0	1
Pagurus pollicaris	1	0	0	1	1	0	1	1	1&3	1	0	0	0	1	0	0	1
Pagurus bullisi	1	0	0	1	1	0	1	1	1&3	1	0	0	0	1	0	0	1
Iridopagurus caribbensis	?	?	?	?	?	?	?	?	?	?	?	?	?	?	?	?	?
Iridopagurus reticulatus	?	?	?	?	?	?	?	?	?	?	?	?	?	?	?	?	?
Xylopagurus cancellarius	0	0	?	1	1	0	1	?	?	?	?	?	?	?	?	?	?
Labidochirus splendescens	?	?	?	?	?	?	?	?	?	1	0	?	0	1	0	0	?
Porcellanopagurus filholi	1	0	0	0	2	0	1	0	1	?	?	0	?	?	?	?	?
Gorepagurus piercei	?	?	?	?	?	?	?	?	?	?	?	?	?	?	?	?	?
Manucomplanus ungulatus	?	?	?	?	?	?	?	?	?	?	?	?	?	?	?	?	?
Pylopagurus discoidalis	?	?	?	?	?	?	?	?	?	?	?	?	?	?	?	?	?
Pylopaguridium markhami	?	?	?	?	?	?	?	?	?	?	?	?	?	?	?	?	?
Phimochirus holthuisi	?	?	?	?	?	?	?	?	?	0	0	?	0	1	1	0	?
Phimochirus randalli	?	?	?	?	?	?	?	?	?	0	0	?	0	1	1	0	?
Phimochirus randalli	?	?	?	?	?	?	?	?	?	0	0	?	0	1	1	0	?
Agaricochirus alexandri	?	?	?	?	?	?	?	?	?	?	?	?	?	?	?	?	?
Tomopagurus merimaculosus	?	?	?	?	?	?	?	?	?	?	?	?	?	?	?	?	?
Discorsopagurus schmitti	0	0	0	?	?	0	1	1	?	0	0	0	0	1	1	0	?
Bythiopagurus macrocolus	0	0	0	?	?	0	1	1	?	?	?	?	?	?	?	?	?
Sympagurus dimorphus	0	0	0	1	1	0	1	1	1	0	?	?	?	?	?	?	?
Sympagurus acinops	0	0	0	1	1	0	1	1	1	0	?	?	?	?	?	?	?
Sympagurus pictus	0	0	0	1	1	0	1	1	1	0	?	?	?	?	?	?	?
Sympagurus sp.	0	0	0	1	1	0	1	1	1	0	?	?	?	?	?	?	?
Parapagurus latimanus	?	0	0	1	1	0	1	1	?	0	0	0	0	1	2	0	1
Trizocheles spinosus	?	?	1	?	?	0	?	?	?	0	0	0	0	1	0	1	0
Pomatocheles jeffreysii	?	?	1	?	?	0	?	?	?	0	0	0	0	1	0	?	0
Pachycheles haigae	1	0	1	0	0	1	1	0	?	1	0	0	0	1	0	0	2
Pachycheles rudis	1	0	1	0	0	1	1	0	?	1	0	0	0	1	0	0	2
Pachycheles rugimanus	1	0	1	0	0	1	1	0	?	1	0	0	0	1	0	0	2
Pachycheles ackleinus	1	0	1	0	0	1	1	0	?	1	0	0	0	1	0	0	2
Pachycheles pilosus	1	0	1	0	0	1	1	0	?	1	0	0	0	1	0	0	2
Polyonyx gibbesii	1	0	1	0	0	1	1	0	?	1	0	0	0	1	0	0	2
Pisidia magdalenensis	1	0	1	0	0	1	1	0	?	1	0	0	0	1	0	0	2
Megalobrachium poeyi	?	?	?	?	?	?	?	?	?	1	0	0	0	1	0	0	2
Petrolisthes armatus	1	0	1	0	0	1	1	0	2	1	0	0	0	1	0	0	2
Petrolisthes armatus	1	0	1	0	0	1	1	0	2	1	0	0	0	1	0	0	2
Petrolisthes laevigatus	1	0	1	0	0	1	1	0	2	1	0	0	0	1	0	0	2
Petrolisthes galathinus	1	0	1	0	0	1	1	0	2	1	0	0	0	1	0	0	2
Neopisosoma angustifrons	?	?	?	?	?	?	?	?	?	1	0	0	0	1	0	0	2
Parapetrolisthes tortugensis	?	?	?	?	?	?	?	?	?	1	0	0	0	1	0	0	2
Parapetrolisthes tortugensis	?	?	?	?	?	?	?	?	?	1	0	0	0	1	0	0	2
Allopetrolisthes spinifrons	?	?	?	?	?	?	?	?	?	1	0	0	0	1	0	0	2

Species	135	136	137	138	139	140	141	142	143	144	145	146	147	148	149	150	151
Porcellana sayana	?	0	1	0	0	1	1	0	?	1	0	0	0	1	0	0	2
Euceramus sp.	?	0	1	0	0	1	1	0	?	1	1	0	0	0	0	0	2
Munida subrugosa	1	0	0	1	0	0	1	0	2	1	1	0	0	1	0	0	2
Munida quadrispina	1	0	0	1	0	0	1	0	2	1	1	0	0	1	0	0	2
Munida iris	1	0	0	1	0	0	1	0	2	1	1	0	0	1	0	0	2
Munida pusilla	1	0	0	1	0	0	1	0	2	1	1	0	0	1	0	0	2
Pleuroncodes monodon	1	0	0	1	0	0	1	0	?	1	1	0	0	1	0	0	2
Cervimunida johni	1	0	0	1	0	0	1	0	?	1	?	?	?	?	?	?	?
Sadayoshia sp.	1	0	0	1	0	0	1	0	?	1	1	0	0	1	0	0	1
Babamunida kanaloa	1	0	0	1	0	0	1	0	?	?	?	0	?	?	?	?	?
Agononida procera	1	0	0	1	0	0	1	0	?	1	1	0	0	1	0	0	2
Neonida grandis	1	0	0	1	0	0	1	0	?	1	1	0	0	1	?	0	2
Anoplonida inermis	1	0	0	1	0	0	1	0	?	?	?	0	?	?	?	?	?
Bathymunida balssi	1	0	0	1	0	0	1	0	?	?	?	?	?	?	?	?	?
Alainius crosnieri	1	0	0	1	0	0	1	0	?	?	?	?	?	?	?	?	?
Galathea sp.	1	0	0	1	0	0	1	0	?	1	1	0	0	1	0	0	1
Galathea rostrata	1	0	0	1	0	0	1	0	?	1	1	0	0	1	0	0	1
Leiogalathea laevirostris	1	0	0	1	0	0	1	0	?	?	?	?	?	?	?	?	?
Shinkaia crosnieri	?	?	?	?	?	?	?	?	?	?	?	?	?	?	?	?	?
Munidopsis bairdii	1	0	0	1	0	0	1	0	1	0	0	0	0	1	0	0	0
Munidopsis erinacea	1	0	0	1	0	0	1	0	1	0	0	0	0	1	0	0	0
Pseudomunida fragilis	0	0	?	0	1	0	1	0	?	?	?	?	?	?	?	?	?
Galacantha rostrata	?	0	0	1	0	0	1	0	?	?	?	0	?	?	?	?	?
Galacantha valdiviae	?	0	0	1	0	0	1	0	?	?	?	0	?	?	?	?	?
Eumunida picta	0	0	0	0	1	0	1	0	?	0	0	0	0	1	1	0	1
Eumunida picta	0	0	0	0	1	0	1	0	?	0	0	0	0	1	1	0	1
Eumunida funambulus	0	0	0	0	1	0	1	0	?	0	0	0	0	1	1	0	1
Uroptychus spinirostris	0	0	0	0	1	0	1	0	1	0	0	0	1	1	1	1	?
Uroptychus nitidus	0	0	0	0	1	0	1	0	1	0	0	0	1	1	1	1	?
Uroptychus parvulus	0	0	0	0	1	0	1	0	1	0	0	0	1	1	1	1	?
Uroptychus scambus	0	0	0	0	1	0	1	0	1	0	0	0	1	1	1	1	?
Gastroptychus novaezelandiae	0	0	0	0	1	0	1	0	?	0	?	0	1	1	1	?	?
Gastroptychus rogeri	0	0	0	0	1	0	1	0	?	0	?	0	1	1	1	?	?
Gastroptychus spinifer	0	0	0	0	1	0	1	0	?	0	?	0	1	1	1	?	?
Chirostylus novaecaledoniae	0	0	0	0	1	0	1	0	?	0	0	0	1	0	?	1	0
Kiwa hirsuta	?	?	?	?	?	?	?	?	?	?	?	?	?	?	?	?	?
Aegla violacea	0	0	0	0	1	0	1	0	?	?	?	?	?	?	?	?	?
Aegla uruguyana	0	0	0	0	1	0	1	0	?	?	?	?	?	?	?	?	?
Aegla platensis	0	0	0	0	1	0	1	0	?	?	?	?	?	?	?	?	?
Aegla papudo	0	0	0	0	1	0	1	0	?	?	?	?	?	?	?	?	?
Aegla jarai	0	0	0	0	1	0	1	0	?	?	?	?	?	?	?	?	?
Aegla cholchol	0	0	0	0	1	0	1	0	?	?	?	?	?	?	?	?	?
Aegla camargoi	0	0	0	0	1	0	1	0	?	?	?	?	?	?	?	?	?
Aegla abtao	0	0	0	0	1	0	1	0	?	?	?	?	?	?	?	?	?
Aegla alacalufi	0	0	0	0	1	0	1	0	?	?	?	?	?	?	?	?	?
Lomis hirta	0	0	1	0	1	0	?	0	?	0	0	0	1	1	0	0	?

Species	152	153	154	155	156
Solenocera sp.	?	0	0	2	2
Hymenopenaeus debilis	?	0	0	2	2
Atyopsis sp.	-	0	-	-	-
Latreutes fucorum	0	0	1	2	2
Ogyrides sp.	0	0	0	2	2
Palaemonetes pugio	0	0	0	2	1
Calastacus crosnieri	?	0	?	?	?
Calaxius manningi	?	0	?	?	?
Lepidophthalmus louisianensis	1	1	0	2	0
Sergio mericeae	1	?	0	2	0
Austinogebia narutensis	0	0	0	0	0
Laemedia astacina	0	0	0	2	?
Thalassina anomala	?	0	?	?	?
Cosmonotus grayi	?	0	?	?	2
Calappa gallus	1	0	1	2	1
Chorilia longipes	?	0	1	2	0
Cyclograpsus cinereus	2	0	0	2	2
Praebebalia longidactyla	?	?	?	?	?
Blepharipoda occidentalis	0	1	0	2	2
Emerita emeritus	0	0	0	2	2
Emerita brasiliensis	0	0	0	2	2
Emerita talpoida	0	0	0	2	2
Albunea gibbesii	0	0	1	2	2
Albunea catherinae	0	0	1	2	2
Zygopa michaelis	?	?	?	?	?
Lepidopa californica	0	0	1	2	1
Lepidopa dexterae	0	0	1	2	1
Paraleucolepidopa	0	0	1	2	1
Coenobita compressus	2	1	0	2	2
Coenobita clypeatus	2	1	0	2	2
Coenobita perlatus	2	1	0	2	2
Birgus latro	2	1	0	2	2
Clibanarius albidigitus	1	1	0	2	1
Cilbanarius antillensis	1	1	0	2	1
Clibanarius corallinus	1	1	0	2	1
Clibanarius vittatus	1	1	0	2	1
Isocheles pilosus	1	1	0	2	1
Isocheles wurdmenni	1	1	0	2	1
Calcinus obscurus	1	1	0	2	2
Calcinus laevimanus	1	1	0	2	2
Paguristes turgidus	1	1	0	2	1
Paguristes tortugae	1	1	0	2	1
Paguristes triangulatus	1	1	0	2	1
Paguristes moorei	1	1	0	2	1
Paguristes sericeus	1	1	0	2	1
Paguristes grayi	1	1	0	2	1
Paguristes puncticeps	1	1	0	2	1
Paguristes cadenati	1	1	0	2	1
Areopaguristes hewatti	1	1	0	2	1
Areopaguristes hewatti	1	1	0	2	1
Areopaguristes hewatti	1	1	0	2	1
Areopaguristes pilosus	1	1	0	2	1
Areopaguristes hummi	1	1	0	2	1
Areopaguristes hummi	1	1	0	2	1
Dardanus fuscous	0	1	0	2	2
Dardanus insignis	0	1	0	2	2
Dardanus sp.	0	1	0	2	2
Petrochirus diogenes	1	1	0	2	2

Species	152	153	154	155	156
Lithodes santolla	2	1	0	2	1
Lithodes santolla	2	1	0	2	1
Glyptolithodes cristatipes	2	1	0	2	?
Paralomis sp.	2	1	0	2	0
Phyllolithodes papillosus	?	?	?	?	?
Lopholithodes mandtii	2	1	0	2	1
Paralithodes brevipes	2	1	0	2	1
Paralithodes camtschaticus	2	1	0	2	1
Paralithodes platypus	2	1	0	2	1
Cryptolithodes sp.	2	1	0	2	1
Oedignathus inermis	?	?	?	?	?
Hapalogaster mertensii	2	0	0	2	1
Pagurus bernhardus	2	1	0	2	1
Pagurus stimpsoni	2	1	0	2	1
Pagurus carolinensis	2	1	0	2	1
Pagurus brevidactylus	2	1	0	2	1
Pagurus maclaughlinae	2	1	0	2	1
Pagurus pollicaris	2	1	0	2	1
Pagurus bullisi	2	1	0	2	1
Iridopagurus caribbensis	?	?	?	?	?
Iridopagurus reticulatus	?	?	?	?	?
Xylopagurus cancellarius	?	?	?	?	?
Labidochirus splendescens	2	1	0	2	1
Porcellanopagurus filholi	?	?	?	?	?
Gorepagurus piercei	?	?	?	?	?
Manucomplanus ungulatus	?	?	?	?	?
Pylopagurus discoidalis	?	?	?	?	?
Pylopaguridium markhami	?	?	?	?	?
Phimochirus holthuisi	2	1	0	2	1
Phimochirus randalli	2	1	0	2	1
Phimochirus randalli	2	1	0	2	1
Agaricochirus alexandri	?	?	?	?	?
Tomopagurus merimaculosus	?	?	?	?	?
Discorsopagurus schmitti	2	1	0	2	1
Bythiopagurus macrocolus	?	?	?	?	?
Sympagurus dimorphus	?	?	?	?	?
Sympagurus acinops	?	?	?	?	?
Sympagurus pictus	?	?	?	?	?
Sympagurus sp.	?	?	?	?	?
Parapagurus latimanus	2	1	0	0	2
Trizocheles spinosus	0	1	0	0&1	0
Pomatocheles jeffreysii	0	?	0	1	0
Pachycheles haigae	0	1	0	2	0
Pachycheles rudis	0	1	0	2	0
Pachycheles rugimanus	0	1	0	2	0
Pachycheles ackleinus	0	1	0	2	0
Pachycheles pilosus	0	1	0	2	0
Polyonyx gibbesii	0	1	0	2	0
Pisidia magdalenensis	0	1	0	2	0
Megalobrachium poeyi	0	1	0	2	0
Petrolisthes armatus	0	1	0	2	0
Petrolisthes armatus	0	1	0	2	0
Petrolisthes laevigatus	0	1	0	2	0
Petrolisthes galathinus	0	1	0	2	0
Neopisosoma angustifrons	0	1	0	2	0
Parapetrolisthes tortugensis	0	1	0	2	0
Parapetrolisthes tortugensis	0	1	0	2	0
Allopetrolisthes spinifrons	0	1	0	2	0

Species	152	153	154	155	156
Porcellana sayana	0	1	0	2	0
Euceramus sp.	0	1	0	2	0
Munida subrugosa	0	1	0	2	1
Munida quadrispina	0	1	0	2	1
Munida iris	0	1	0	2	1
Munida pusilla	0	1	0	2	1
Pleuroncodes monodon	0	1	0	2	1
Cervimunida johni	?	?	?	?	?
Sadayoshia sp.	0	1	0	2	1
Babamunida kanaloa	?	?	?	?	?
Agononida procera	1	1	0	2	1
Neonida grandis	1	1	0	2	1
Anoplonida inermis	?	?	?	?	?
Bathymunida balssi	?	?	?	?	?
Alainius crosnieri	?	?	?	?	?
Galathea sp.	1	1	0	2	2
Galathea rostrata	1	1	0	2	2
Leiogalathea laevirostris	?	?	?	?	?
Shinkaia crosnieri	?	?	?	?	?
Munidopsis bairdii	0	1	0	2	0
Munidopsis erinacea	0	1	0	2	0
Pseudomunida fragilis	?	?	?	?	?
Galacantha rostrata	?	?	?	?	?
Galacantha valdiviae	?	?	?	?	?
Eumunida picta	2	1	0	2	1
Eumunida picta	2	1	0	2	1
Eumunida funambulus	2	1	0	2	1
Uroptychus spinirostris	?	0	2	1	0
Uroptychus nitidus	?	0	2	1	0
Uroptychus parvulus	?	0	2	1	0
Uroptychus scambus	?	0	2	1	0
Gastroptychus novaezelandiae	?	0	4	1	0
Gastroptychus rogeri	?	0	4	1	0
Gastroptychus spinifer	?	0	4	1	0
Chirostylus novaecaledoniae	0	0	3	1	0
Kiwa hirsuta	?	?	?	?	?
Aegla violacea	?	?	?	?	?
Aegla uruguyana	?	?	?	?	?
Aegla platensis	?	?	?	?	?
Aegla papudo	?	?	?	?	?
Aegla jarai	?	?	?	?	?
Aegla cholchol	?	?	?	?	?
Aegla camargoi	?	?	?	?	?
Aegla abtao	?	?	?	?	?
Aegla alacalufi	?	?	?	?	?
Lomis hirta	1	1	0	2	?
