# Supplementary material for: A comprehensive and integrative reconstruction of evolutionary history for Anomura (Crustacea: Decapoda)
Source: BMC Evol Biol. 2013 Jun 20;13:128. doi: 10.1186/1471-2148-13-128 (PMC3708748; doi:10.1186/1471-2148-13-128)
Supplement: Additional file 3 — Morphological characters and states used in combined Bayesian analysis. [file 1471-2148-13-128-S3.doc]

# Additional File 3.

# Anomura morphological characters and states

1. Rostrum: distinct (0); absent or minute (1) (Dixon et al. 2003).

2. Rostral form: simple, elongate (0); triangular or subtriangular (2); broadly rounded, broadly and weakly subacute or lobate (3).

3. Median dorsal rostral process: absent (0); present (1). The median dorsal rostral process is a feature of *Lithodes*, and for convenience, is often referred to as the rostrum in taxonomic literature. The true rostrum in *Lithodes* is a simple spine often termed the ‘ventral rostral spine’ for taxonomic purposes (Ahyong 2010)

4. Carapace margins: indistinct (0); distinctly defined (1) (Scholtz & Richter 1995).

5. Carapace regions (hepatic, gastric, cardiac, branchial): undefined (0); weakly well defined (1); distinctly delineated (2) (Schnabel et al. 2011).

6. Cervical groove: present (0); obsolete or absent (1).

7. Carapace surface spination: unarmed or with few scattered spines (0); densely spinose (1).

8. Carapace proportions: elongate (0); as long as wide to wider than long (1) (Schnabel et al. 2011).

9. Anterior portion of carapace: well calcified throughout (0); moderately or partially calcified (1) (Schnabel et al. 2011).

10. Posterior portion of carapace: well-calcified (0); soft or poorly calcified (1) (Schnabel et al. 2011).

11. Carapace dorsoventral shape: subcylindrical (0); depressed (1) (Schnabel et al. 2011).

12. Carapace dorsal outline: subquadrate (0); ovate (2); subtriangular (3).

13. Branchiostegites: fully calcified (0); partially calcified (1); membranous (2) (Schnabel et al. 2011).

14. Carapace anterolateral spine: absent (0); present (1) (McLaughlin et al., 2007).

15. Carapace supraorbital projections: absent (0); short tooth (1); long spine (2) (McLaughlin et al., 2007).

16. Second supraorbital spine: absent (0); present (1). The galatheid *Sadayoshia*, and eumunidid *Eumunida* have a second pair of supraorbital spines.

17. Carapace antennal spine: absent (0); present (1).

18. Carapace outer orbital spines: absent (0); present (1).

19. Carapace pterygostomian spine: absent (0); present (1).

20. Carapace linea anomurica/thalassinica: absent (0); present (1). The *lineae* *anomurica* and *thalassinica* are considered homologous (Ahyong & O’Meally 2004; McLaughlin et al. 2007)

21. Sternite-coxa articulation: regular (0); inverted (1) (Scholtz and Richter, 1995).

22. Thoracic sternal plastron: narrow (0); widening posteriorly (1) (Dixon et al. 2003).

23. Thoracic sternite 4 median fissure: absent (0); present (1). The median fissure of thoracic sternite 4 is a feature of several lithodid genera (Ahyong 2010).

24. Thoracic sternite 3: narrow, with bases of corresponding maxilliped 3 contacting or nearly so (0); broad, with bases of corresponding appendages distinctly separated (1) (Schnabel et al. 2011).

25. Thoracic sternite 3 anterior margin: rounded to angular (0); produced to prominent median projection (1); with small anterior point (2); transverse (3).

26. Thoracic sternite 3 with anterior ‘cliff’: absent (0); present (1) (Baba 2005).

27. Thoracic sternite 3 anterior margin of overhang: more or less straight, unbroken (0); with V-shaped emargination (Baba 2005).

28. Thoracic sternite 3 anterior margin spine row: absent (0); present (1) (Baba 2005).

29. Thoracic sternite 4 anterolateral spine or point: absent (0); present (1).

30. Thoracic sternite 6 anterior median lobe: obsolete (0); rounded (1); rectangular (2).

31. Fusion of thoracic sternite 6 (3rd pereopods) with sternite 5: completely fused (0); incompletely or indistinctly fused (1); not fused (2) (Scholtz & Richter 1995).

32. Thoracic sternite 8 (5th pereopods): well developed (0); reduced (1); absent (2) (Schnabel et al. 2011).

33. Thoracic sternite 7/8: fused (0); articulated (1).

34.Thoracic sternite 8 and abdominal somite 1: separate (0); fused (1) (Dixon et al., 2003).

35. Female brood pouch: absent (0); present (1). The brood pouch is a feature of some diogenid hermit crabs.

36. Gill type: trichobranchiate (0); phyllobranchiate (1); dendrobranchiate (2).

37. Branchial condition: normal (0); reduced (1) (Schnabel et al. 2011).

38. Abdominal segmentation: somites distinct (0); somites ill-defined (1) (Schnabel et al. 2011).

39. Abdomen flexion (dorsovental): slightly or weakly flexed (0); strongly flexed and carried against the ventral thorax (at least in males) (1) (McLaughlin et al. 2007).

40. Abdomen condition: straight (0); twisted (1) (Schnabel et al. 2011).

41. Abdominal calcification: strong with complete sternites (0); strong with incomplete sternites (1); weak with incomplete sternites (2) (Schnabel et al. 2011).

42. Pleonic hinges: weak (0); strong (1).

43. Abdominal pleura: prominent (0); reduced (1); absent (Schnabel et al. 2011).

44. Abdominal somite 1 overlapping lobes: absent (0); present (1) (Dixon et al. 2003).

45. Abdominal tergite 1: well calcified (0); partially calcified (1); chitinous or membranous (2) (Schnabel et al. 2011).

46. Abdominal pleuron 2: not overlapping adjacent pleura (0); overlapping adjacent pleura (1). The expanded pleuron 2, overlapping adjacent pleura is a synapomorphy of Caridea.

47. Abdominal tergite 2 division: undivided (0); five plates (1); absent (2); three plates comprising marginal and fused median and submedian plates (3); three plates comprised of free median plate and fused median and submedian plates (4). The plate divisions reflect differences among lithodid genera (Ahyong 2010).

48. Abdominal somite 5 pleuron: well-developed (0); reduced (1); absent (2).

49. Telson proportions: distinctly longer than wide (0); as wide as long (1); wider than long (2) (Schnabel et al. 2011).

50. Telson shape: triangular or subtriangular (0); subquadrate to linguiform (1); subsemicircular (2) (Schnabel et al. 2011).

51. Telson sexual dimorphism: weak or absent (0); distinct (1).

52. Telson dorsal surface: undivided (0); divided into multiple small plates (1); divided by longitudinal suture into two plates (2); divided transversely (3) (Schnabel et al. 2011).

53. Telson terminal margin: entire (0); with median cleft or concavity (1) (Schnabel et al. 2011).

54. Telson stretch receptor: absent (0); present (1) (Scholtz & Richter 1995; Paul 2003).

55. Uropods: present (0); absent (1).

56. Uropod rasp: absent (0); present (1).

57. Uropod shape: foliaceous, forming tail fan (0); styliform, not forming tail fan (1); short, bulbous (2) (Dixon et al. 2003; Ahyong & O’Meally 2004; McLaughlin et al. 2007).

58. Eyes: well-developed (0); absent or vestigial (1) (Schnabel et al. 2011).

59. Eye mobility: movable (0); immovable (1) (Schnabel et al. 2011).

60. Ocular scales spination: simple (0); multispinose (1); bifid (2).

61. Orbit: present (0); very slight or absent (1) (Schnabel et al. 2011).

62. Ocular peduncle setation: glabrous or sparsely setose (0); with ring of 'eye lashes' surrounding cornea (1); with dorsal field of dense setae (2) (Schnabel et al. 2011).

63. Ocular peduncle spines: absent (0); present (1).

64. Eyestalk length: squat (0); long, slender (1).

65. Ocular peduncle shape: cylindrical (0); dorsoventrally flattened (1); laterally compressed (2).

66. Ocular acicles: absent (0); present (1) (McLaughlin et al., 2007).

67. Mandibular cutting edge: chitinous (0); calcified (1) (Schnabel et al. 2011).

68. Mandibular dentition: smooth (0); strongly toothed (1); feebly toothed, tridentate (2).

69. Mandibular palp: three-segmented (0); two-segmented (1); absent (2).

70. Maxillule endopod segmentation: two (0); one (1) (Schnabel et al. 2011).

71. Maxillule endopod external lobe: vestigial or absent (0); prominent (1) (Schnabel et al. 2011).

72. Caridean lobe: absent (0); present (1).

73. Maxilliped 1 exopod flagellum: well-developed (0); absent or reduced (1) (Schnabel et al. 2011).

74. Maxilliped epipod 1: absent (0); present.

75. Maxilliped epipod 2: absent (0); present.

76. Maxilliped 3 form: pediform (0); broadly expanded or operculate (1) (Schnabel et al. 2011).

77. Maxilliped 3 crista dentata: well developed (0); weakly developed or absent (1) (Schnabel et al. 2011).

78. Maxilliped 3 merus form: elongate, cylindrical (0); elongate, rectilinear compressed (1); with wide flange (2); elongate, triangular, compressed (3).

79. Maxilliped 3 carpal distal projection: short or absent (0); long, prominent (Boyko 2002)

80. Maxilliped 3 exopod flagellum: present (0); absent (1).

81. Maxilliped 3 crista dentata accessory tooth: absent (0); present (1) (Dixon et al. 2003).

82. Maxilliped 3 epipod: present (0); absent (1).

83. Antennular basal article: simple, not notably expanded or enlarged (0); notably expanded or enlarged (1).

84. Antennular flagella: straight (0); strongly curved (1) (Scholtz & Richter 1995).

85. Antennular flagella annulus proportions: annuli subequal (0); annuli wider than long (1) (Scholtz & Richter 1995; Dixon et al. 2003).

86. Antennular flagella length: longer than antennular peduncle (0); similar to antennular peduncle (1); shorter than peduncle (2) (Dixon et al. 2003).

87. Antennular peduncle shape: Z-shaped (0); straight (1) (Dixon et al., 2003).

88. Antennular outer flagellum: basal segments free (0); some basal segments fused (1) (Scholtz & Richter 1995).

89. Antennular upper flagellum termination: tapering (0); blunt, stick-like (1).

90. Antenna article 1 spine ventral to dorsodistal angle: absent (0); present (1) (Boyko & Harvey 2009).

91. Antenna position: not excluded from orbit (0); excluded from orbit (1).

92. Antennal basal article position: not in notch (0); in carapace notch (1) (Scholtz & Richter 1995).

93. Antennal peduncle segmentation: articles 2 and 3 indistinctly fused (0); articles 2 and 3 free (1); 2 and 3 indistinguishably fused (2) The antennal articles are free in most anomurans, but galatheoids have articles 2 and 3 indistinguishably fused, and in aeglids, articles 2 and 3 are immovably fused, but with the demarcation evident.

94. Basal antennal article: movable, not fused to carapace or epistome (0), immovable, fused to carapace or epistome (1).

95. Antennal gland position: ventral (0); lateral (1).

96. Scaphocerite: absent (0); well-developed (1); minute (2).

97. Scaphocerite mobility: articulated (0); fused to peduncle (1). In most taxa having a scaphocerite, the articulation is free. However, in aeglids and kiwaids, the scaphocerite appears to be present but immovably fused to the antennal peduncle.

98. Pereopod 1 condition: chelate (0); subchelate (1); simple (2).

99. Pereopod 1 symmetry: equal or subequal (0); left distinctly larger (1); right distinctly larger (2).

100. Pereopod 1 chela: swollen, cross-section subcylindrical to ovate (0); compressed, flattened (1).

101. Pereopod 1 terminus of dactylus and pollex: both corneous (0); both calcified (1); one cheliped with corneous and one with calcified terminus (2) (Schnabel et al. 2011).

102. P1 dactylus apex: pointed (0); rounded (1).

103. Pereopod 2 condition: semichelate (0); simple (1); chelate (2) (Schnabel et al. 2011).

104. Pereopod 2 merus setal row: absent (0); present (1) (Dixon et al. 2003).

105. Pereopod 2–3 dactyli: sub-conical with subcircular cross-section (0); laterally compressed and dorsoventrally expanded (1) (Schnabel et al. 2011).

106. Pereopod 2–4 dactyl corneous apical claws: present (0); absent (1).

107. Pereopod 3 condition: chelate (0); simple (1).

108. Pereopod 4 condition: simple (0); subchelate (1); chelate (2).

109. Pereopod 4 size: normal, similar to preceding limb (0); strongly reduced (1).

110. Pereopod 4 preungal process: absent (0); small, inconspicuous (1); prominent (2).

111. Pereopod 4 merus lateral surface: calcified (0); decalcified (1).

112. Pereopod 4–5 rasp: absent (0); present (1).

113. Pereopod 5 termination: simple or subchelate (0); chelate (1).

114. Pereopod 5 dactyl scale-like teeth: absent (0); present (1).

115. Male pleopod pairing: paired (0); unpaired (1).

116. Male pleopod 1: present (0); absent (1).

117. Male pleopod 1 rami: biramous (0); uniramous (1).

118. Male pleopod 2: present (0); vestigial or absent (1) (McLaughlin et al. 2007).

119. Male pleopods 3–4: present (0); vestigial or absent (1).

120. Male sexual tube: absent (0); present (1).

121. Female paired pleopods 1: absent (0); present (1).

122. Female pleopods 2: present (0); absent (1) (McLaughlin et al. 2007).

123. Female pleopods 2 pairing: symmetrical paired (0); asymmetrically paired (1); unpaired (2).

124. Female pleopods 3–4 development: biramous (0); uniramous (1).

125. Female pleopods 3: paired (0); unpaired (1); absent (2).

126. Female pleopods 4: paired (0); unpaired (1); absent (2).

127. Female pleopod 5: paired (0); unpaired (1); vestigial or absent (2).

128. Development: indirect (0); direct (1) (Scholtz & Richter 1995).

129. Spawning: broadcast (0); incubated (1).

130. Sperm acrosome shape: spherical to ovoid (0); elongated (1); depressed (2) (Jamieson and Tudge 2000; Tudge et al. 2001; Tudge & Scheltinga 2002).

131. Acrosome position relative to cytoplasm: acrosome vesicle not embedded in cytoplasm (0); acrosome vesicle embedded in cytoplasm (1) (Tudge & Scheltinga 2002).

132. Acrosome ray zone: absent (0); present (1).

133. Microtubular arms: nuclear origin (0); cytoplasmic origin (1); absent (2) (Tudge 1997).

134. Spermatozoal number of external (microtubular) arms: 3 (0); 4 or more (1); 1 or 2 (2) (Jamieson & Tudge 2000; Tudge et al. 2001; Tudge & Scheltinga 2002).

135. Ornamentation of perforatorial chamber wall: walls smooth (0); with shallow corrugations or prominent longitudinal septa (1) (Jamieson & Tudge 2000; Tudge et al. 2001; Tudge & Scheltinga 2002).

136. Microvillar projections in base of acrosomal chamber: absent (0); present (1) (Jamieson & Tudge 2000; Tudge et al. 2001; Tudge & Scheltinga 2002).

137. Operculum perforation: imperforate (0); centrally perforate (1) (Jamieson & Tudge 2000; Tudge et al. 2001; Tudge & Scheltinga 2002).

138. Reticulated acrosome zones: absent (0); present (1) (Jamieson & Tudge 2000; Tudge et al. 2001; Tudge & Scheltinga 2002).

139. Inner acrosome zone: divided (0); entire (1); absent (2) (Jamieson & Tudge 2000; Tudge et al. 2001; Tudge & Scheltinga 2002).

140. Posterior perforatorial ring: absent (0); present (1) (Jamieson & Tudge 2000; Tudge et al. 2001; Tudge & Scheltinga 2002).

141. Tripartite spermatophore (ampulla, stalk and pedestal): absent (0); present (1) (Jamieson & Tudge 2000; Tudge et al. 2001; Tudge & Scheltinga 2002).

142. Spermatophore accessory ampullae: present (0); absent (1) (Jamieson & Tudge 2000; Tudge et al. 2001; Tudge & Scheltinga 2002).

143. Form of stalk in pedunculate spermatophore: spermatophore tubular or capsular (0); pedunculate with small pseudostalk (1); pedunculate with short, thick stalk (2); pedunculate with long, thin stalk (3) (Jamieson & Tudge 2000; Tudge et al. 2001; Tudge & Scheltinga 2002).

144. Zoea 1 carapace posterolateral spine: absent (0); present (1) (Schnabel et al. 2011).

145. Zoea 1 posterodorsal and posteroventral teeth: absent (0); present (1) (Schnabel et al. 2011).

146. Zoeal mandibles: similar (0); markedly asymmetrical (1).

147. Zoea 1 antennular peduncle: unsegmented (0); segmented (1). The antennule is segmented in the chirostylids, lomisids and some lithodids, probably due to their abbreviated development in which they hatch at an advanced stage (Cormie 1993; McLaughlin et al. 2003; Clark & Ng 2008).

148. Zoea 1 antennal scaphocerite: absent (0); present (1).

149. Zoea 1 antenna endopod: 0–1 terminal plumose seta (0); 2 terminal long plumose setae (1); 3 terminal plumose setae (2) (Schnabel et al. 2011).

150. Zoea 1 mandibular palp: absent or a small bud (0); distinct (1).

151. Zoea 1 maxillule basial endite stout cuspidate setae: absent (0); two (1); three or more (2) (Schnabel et al. 2011).

152. Zoea 1 maxillule endopod segmentation: unsegmented (0); two-segmented (1); three-segmented (2).

153. Anomuran hair (seta): absent (0); present (1).

154. Zoea 1 paired telson lateral spines: absent (0); one (1); two (2); three (3); more than 10 (4). Lateral spines on the telson are nearly always absent, with the exception of *Lepidopa* and *Galathea,* each of which have one pair of spines*.* Chirostylidae, however, have between 2 and 10 pairs of spines along the lateral margins.

155. Zoea 1 uropods: uniramous (0); biramous (1); absent (2).

156. Zoeal stages: abbreviated, 2 zoeal stages (0); 3 or 4 zoeal stages (1); 5 or more zoeal stages (2).

REFERENCES CITED IN CHARACTER LIST

Ahyong ST (2010) The marine fauna of New Zealand: king crabs of New Zealand, Australia and the Ross Sea (Crustacea: Decapoda: Lithodidae). *NIWA Biodiversity Memoir* **123**: 1–196.

Ahyong ST, O'Meally D (2004) Phylogeny of the Decapoda Reptantia: resolution using three molecular loci and morphology. *Raffles Bulletin of Zoology* **52**: 673–693.

Baba K (2005) Deep-sea chirostylid and galatheid crustaceans (Decapoda: Anomura) from the Indo-West Pacific, with a list of species. *Galathea Reports* **20**: 1–317.

Boyko CB (2002) A worldwide revision of the Recent and fossil sand crabs of the Albuneidae Stimpson and Blepharipodidae, new family (Crustacea: Decapoda: Anomura: Hippoidea). *Bulletin of the American Museum of Natural History* **272**: 1–396.

Boyko CB, Harvey AW (2009) Phylogenetic systematics and biogeography of the sand crab families Albuneidae and Blepharipodidae (Crustacea: Anomura: Hippoidea). *Invertebrate Systematics* **23**: 1–18.

Clark PF, Ng PKL (2008) The lecithotrophic zoea of *Chirostylus ortmanni* Miyake & Baba, 1968 (Crustacea: Anomura: Galatheoidea: Chirostylidae) described from laboratory hatched material. Raffles Bulletin of Zoology 56, 85-94.

Cormie AK (1993) The morphology of the first zoea stage of *Lomis hirta* (Lamarck, 1818) (Decapoda, Lomisidae). *Crustaceana* **64**, 249–255.

Dixon CJ, Ahyong ST, Schram FR (2003) A new hypothesis of decapod phylogeny. *Crustaceana* **76**: 935–975.

Jamieson BG, Tudge CC (2000) Crustacea Decapoda. In 'Reproductive Biology of Invertebrates'. (Ed. BG Jamieson) pp. 1–95. (John Wiley & Sons Ltd: New York)

McLaughlin PA, Anger K, Kaffenberger A, Lovrich GA (2003) Larval and early juvenile development in *Paralomis granulosa* (Jacquinot) (Decapoda: Anomura: Paguroidea: Lithodidae), with emphasis on abdominal changes in megalopal and crab stages. *Journal of Natural History* **37**: 1433–1452.

McLaughlin PA, Lemaitre R, Sorhannus U (2007) Hermit crab phylogeny: a reappraisal and its "fall out". *Journal of Crustacean Biology* **21**: 97–115.

Paul DH (2003) Neurobiology of the Anomura: Paguroidea, Galatheoidea and Hippoidea. *Memoirs of Museum Victoria* **60**: 3–11.

Schnabel KE, Ahyong ST, Maas EW (2011) Galatheoidea are not monophyletic – molecular and morphological phylogeny of the squat lobsters (Decapoda: Anomura) with recognition of a new superfamily. *Molecular Phylogenetics and Evolution* **58**: 157–168.

Scholtz G, Richter S (1995) Phylogenetic systematics of the reptantian Decapoda (Crustacea, Malacostraca). *Zoological Journal of the Linnean Society* **113**: 289–328.

Tudge CC, Scheltinga DM, Jamieson BG (2001) Spermatozoal morphology in the "symmetrical" hermit crab, *Pylocheles* (*Bathycheles*) sp. (Crustacea, Decapoda, Anomura, Paguroidea, Pylochelidae). *Zoosystema* **23**: 117–130.

Tudge CC, Scheltinga DM (2002) Spermatozoal morphology of the freshwater anomuran *Aegla longirostri* Bond-Buckup & Buckup, 1994 (Crustacea: Decapoda: Aeglidae) from South America. *Proceedings of the Biological Society of Washington* **115**: 118–128.

Tudge CC (1997) Phylogeny of the Anomura (Decapoda, Crustacea): spermatozoa and spermatophore morphological evidence. *Contributions to Zoology* **67**: 125–141.

SOURCES OF LARVAL AND SPERMATOZOAL DATA

Bartilotti C, Calado R, dos Santos A (2008) Complete larval development of the hermit crabs *Clibanarius aequabilis* and *Clibanarius erythropus* (Decapoda: Anomura: Diogenidae), under laboratory conditions, with a revision of the larval features of genus *Clibanarius*. *Helgoland Marine Research* 62:103–121.

Boyd CM (1960) The larval stages of *Pleuroncodes planipes* Stimpson (Crustacea, Decapoda, Galatheidae). *Biological Bulletin* 118:17–30.

Christiansen ME, Anger K (1990) Complete larval development of *Galathea intermedia* Lilljeborg reared in laboratory culture (Anomura: Galatheidae). *Journal of Crustacean Biology* 10: 87–111.

Clark PF, Ng PKL (2008) The lecithotrophic zoea of *Chirostylus ortmanni* Miyake & Baba, 1968 (Crustacea: Anomura: Galatheoidea: Chirostylidae) described from laboratory hatched material. *Raffles Bulletin of Zoology* 56: 85–94.

Cormie AK (1993) The morphology of the first zoea stage of *Lomis hirta* (Lamarck, 1818) (Decapoda, Lomisidae). *Crustaceana* 64: 249–255.

Costlow JD, Jr, Fagetti E (1967) The larval development of the crab *Cyclograpsus cinereus* Dana, under laboratory conditions. *Pacific Science* 21: 166–177.

Crain JA, McLaughlin PA (2000a) Larval and early juvenile development in the Lithodidae (Decapoda: Anomura: Paguroidea) reared under laboratory conditions 1. Subfamily Lithodinae: *Lopholithodes mandtii* Brandt, 1848. *Invertebrate Reproduction and Development* 37:43–59.

Crain JA, McLaughlin PA (2000b) Larval and early juvenile development in the Lithodidae (Decapoda: Anomura: Paguroidea) reared under laboratory conditions 2. Subfamily Hapalogastrinae: *Placetron wosnessenskii* Schalfeew, 1892 with notes on comparative development within the subfamilies of the Lithodidae. *Invertebrate Reproduction and Development* 37:113–127.

Dobkin S (1963) The larval development of *Palaemonetes paludosus* (Gibbes, 1850) (Decapoda, Palaemonidae), reared in the laboratory. *Crustaceana* 6: 41–61.

Fujita Y, Baba K, Shokita S (2001) Larval development of *Galathea inflata* Potts, 1915 (Decapoda: Anomura: Galatheidae) described from laboratory-reared material. *Crustacean Research* 30: 111–132.

Fujita Y, Baba K, Shokita S (2003) Larval development of *Galathea amboinensis* (Decapoda: Anomura: Galatheidae) under laboratory conditions. *Crustacean* *Research* 32: 79–97.

Fujita Y, Clark PF (2010) The larval development of *Chirostylus stellaris* Osawa, 2007 (Crustacea: Anomura: Chirostylidae) described from laboratory reared material. *Crustacean Research* 39: 55–60.

Fujita Y, Shokita S (2005) The complete larval development of *Sadayoshia edwardsii* (Miers, 1884) (Decapoda : Anomura : Galatheidae) described from laboratory-reared material. *Journal of Natural History* 39: 865–886.

Garcia-Guerrero MU, Rodríguez A, Hendrickx ME (2006) Larval development of the eastern Paciic anomuran crab *Porcellana cancrisocialis* (Crustacea: Decapoda: Anomura: Porcellanidae) described from laboratory reared material. *Journal of the Marine Biological Association of the United Kingdom* 86:1123–1132.

Gherardi F, McLaughlin PA (1995) Larval and early juvenile development of the tube-dwelling hermit crab *Discorsopagurus schmitti* (Stevens) (Decapoda: Anomura: Paguridae) reared in the laboratory. *Journal of Crustacean Biology* 15: 258–279.

Gore RH (1968) The larval development of the commensal crab *Polyonyx gibbesi* Haig, 1956 (Crustacea: Decapoda). *Biological Bulletin* 135: 111–129.

Gore RH (1979) Larval development of *Galathea rostrata* under laboratory conditions, with a discussion of larval development in the Galatheidae (Crustacea Anomura). *Fishery Bulletin* 76: 781–806.

Gore RH, Van Dover CL (1981) Studies on decapod Crustacea from the Indian River region of Florida. XIX. Larval development in the laboratory of *Lepidopa richmondi* Benedict, 1903, with notes on larvae of American species in the genus (Anomura: Albuneidae). *Proceedings of the Biological Society of Washington* 93: 1016–1034.

Guerao G, Abelló P, Torres P (1999) Morphology of the first zoea of the shamefaced crab *Calappa granulata* (Linnaeus, 1758) (Brachyura, Calappidae) obtained in the laboratory. *Graellsia* 55: 157–162

Guerao G, Macpherson E, Samadi S, Richer de Forges B, Boisselier M-C (2006) First stage zoeal descriptions of five Galatheoidea species from Western Pacific (Crustacea: Decapoda: Anomura). *Zootaxa* 1227: 1–29.

Gurney R (1938) Larvae of decapod Crustacea. Part 5. Nephropsidea and Thalassinidea. *Discovery Reports* 17: 291–344.

Hart JFL (1965) Life history and larval development of *Cryptolithodes typicus* Brandt (Decapoda, Anomura) from British Columbia. *Crustaceana* 8: 255–276, pl. III.

Hebling NJ, Mansur CB (1995) Desenvolvimento larval de *Dardanus insignis* (Saussure) (Crustacea, Decapoda, Diogenidae), em laboratorio. *Revista Brasilieira de Zoologia* 12: 471–491.

Hong SY, Perry RI, Boutillier JA, Kim MH (2005) Larval development of *Acantholithodes hispidus* (Stimpson) (Decapoda: Anomura: Lithodidae) reared in the laboratory. *Invertebrate Reproduction and Development* 47: 101–110.

Hubschman JH, Broad AC (1974) The larval development of *Palaemonetes intermedius* Holthuis, 1949 (Decapoda, Palaemonidae) reared in the laboratory. *Crustaceana* 26: 89–103.

Ingle RW (1992) *Larval Stages of Northeastern Atlantic Crabs*. An Illustrated Key. Chapman & Hall, London.

Jamieson BG, Guinot D, Richer de Forges B (1995) Phylogeny of the Brachyura (Crustacea: Decapoda): evidence from spermatozoal structure. *Mémoires du Muséum national d'Histoire naturelle, Paris* 166: 265–283.

Jamieson BG, Tudge CC (2000) Crustacea - Decapoda. In: Jamieson BG (ed) *Reproductive Biology of Invertebrates*. John Wiley, New York, pp 95.

Johnson MW, Lewis WM (1942) Pelagic larval stages of the sand crabs *Emerita analoga* (Stimpson), *Blepharipoda occidentalis* Randall, and *Lepidopa myops* Stimpson. *Biological Bulletin* 83: 67–87.

Kim JN, Hong SY (1999) Larval development of *Latreutes laminirostris* (Decapoda: Hippolytidae) reared in the laboratory. *Journal of Crustacean Biology* 19: 762–781.

Kim MH, Hong SY (2000) Larval development of *Cryptolithodes expansus* Miers (Decapoda: Anomura: Lithodidae) reared in the laboratory. *Proceedings of the Biological Society of Washington* 113: 54–56.

Kim MH, Hong SY (2010) Larval development of *Rhinolithodes wosnessenskii* Brandt (Decapoda: Anomura: Lithodidae) reared in the laboratory. *Animal Cells and Systems* 14: 115–123.

Kim MH, Son MH, Hong SY (2008) Larval development of *Pagurus japonicus* (Stimpson) (Decapoda: Anomura: Paguridae) reared in the laboratory. *Animal Cells and Systems* 12: 171–180.

Knight MD (1967) The larval development of the sand crab *Emerita rathbunae* Schmitt (Decapoda, Hippidae). *Pacific Science* 21: 58–76.

Knight MD (1970) The larval development of *Lepidopa myops* Stimpson, (Decapoda, Albuneidae) reared in the laboratory, and the zoeal stages of another species of the genus from California and the Pacific coast of Baja California. *Crustaceana* 19: 125–156.

Ko HS (2001) First zoea of *Enosteoides ornata* (Stimpson, 1858) (Crustacea, Decapoda, Anomura, Porcellanidae) reared under laboratory conditions. *Korean Journal of Biological Sciences* 5: 11–15.

Konishi K (1986) Larval development of the stone crab, *Hapalogaster dentata* (De Haan, 1844) (Crustacea: Anomura: Lithodidae) reared in the laboratory. *Journal of the Faculty of Science, Hokkaido University*, series 6 24: 155–172.

Konishi K, Saito T (2000) Larvae of the deep-sea squat lobsters, *Agononida incerta* (Henderson, 1888) and *Munida striola* Macpherson and Baba, 1993 with notes on larval morphology of the family (Crustacea: Anomura: Galatheidae). *Zoological Science* 17: 1021–1029.

Korn OM, Kornienko ES, Komai T (2008) A reexamination of adults and larval stages of *Diogenes nitidimanus* (Crustacea: Decapoda: Anomura: Diogenidae). *Zootaxa* 1693: 1–26.

Kornienko ES, Korn OM (2012) First stage larva of the lobster shrimp *Allaxius princeps* (Boas, 1880) (Decapoda: Axiidea: Axiidae) obtained in the laboratory. *Zootaxa* 3527: 83–87.

Kornienko ES, Korn OM, Demchuk DD (2012) The larval development of the mud shrimp *Upogebia issaeffi* (Balss, 1913) (Decapoda: Gebiidea: Upogebiidae) reared under laboratory conditions. *Zootaxa* 3269: 31–46.

Lebour MV (1930) The larvae of the Plymouth Galatheidae. I. *Munida banffica, Galathea strigosa* and *Galathea dispersa*. *Journal of the Marine Biological Association of the United Kingdom* 17: 175–187.

Lebour MV (1931) The larvae of the Plymouth Galatheidae. II. *Galathea squamifera* and *Galathea intermedia*. *Journal of the Marine Biological Association of the United Kingdom* 17: 385–390.

Lee SH, Ko HS (2012) Larval stages of *Areopaguristes japonicus* (Miyake, 1961) (Decapoda: Anomura: Diogenidae) described from laboratory reared material. *Zootaxa* 3368: 146–160.

MacDonald JD, Pike RB, Williamson DI (1957) Larvae of the British species of *Diogenes*, *Pagurus*, *Anapagurus* and *Lithodes* (Crustacea, Decapoda). *Proceedings of the Zoological Society of London* 128: 209–257.

Mantelatto FL, Scelzo MA, Tudge CC (2009) Morphological and morphometric appraisal of the spermatophore of the southern hermit crab *Isocheles sawayai* Forest and Saint Laurent, 1968 (Anomura: Diogenidae), with comments on gonopores in both sexes. *Zoologischer Anzeiger* 248: 1–8.

Marques F, Pohle G (1998) The use of structural reduction in phylogenetic reconstruction of decapods and a phylogenetic hypothesis for 15 genera of Majidae: testing previous larval hypotheses and assumptions. *Invertebrate Reproduction and Development* 33: 241–262.

Marques F, Pohle G (2003) Searching for larval support for majoid families (Crustacea: Brachyura) with particular reference to Inachoididae Dana, 1851. *Invertebrate Reproduction and Development* 43: 71–82.

McLaughlin PA, Anger K, Kaffenberger A, Lovrich GA (2001) Megalopal and early juvenile development in *Lithodes santolla* (Molina, 1782) (Decapoda: Anomura: Paguroidea: Lithodidae), with notes on zoeal variations. *Invertebrate Reproduction and Development* 40: 53–67.

McLaughlin PA, Anger K, Kaffenberger A, Lovrich GA (2003) Larval and early juvenile development in *Paralomis granulosa* (Jacquinot) (Decapoda: Anomura: Paguroidea: Lithodidae), with emphasis on abdominal changes in megalopal and crab stages. *Journal of Natural History* 37:1433–1452.

McLaughlin PA, Gherardi F (1995) Larval development in *Phimochirus roseus* (Benedict) (Decapoda: Anomura: Paguridae) reared in the laboratory. *Journal of Natural History* 29:1167–1187.

McLaughlin PA, Gore RH (1988) Studies on the *Provenzanoi* and other pagurid groups: I. The larval stages of *Pagurus maclaughlinae* García-Gómez, 1982 (Decapoda: Anomura: Paguridae), reared under laboratory conditions. *Journal of Crustacean Biology* 8: 262–282.

McLaughlin PA, Gore RH (1992) Studies on the *Provenzanoi* and other pagurid groups: VII. The zoeal and megalopal stages of *Pagurus armatus* (Dana) (Decapoda: Anomura: Paguridae), reared in the laboratory. *Journal of Crustacean Biology* 12: 448–463.

McLaughlin PA, Lemaitre R (2000) Aspects of evolution in the anomuran superfamily Paguroidea: one larval prospective. *Invertebrate Reproduction and Development* 38: 159–169.

McLaughlin PA, Lemaitre R (2008) Larvae of two species of *Trizocheles* (Decapoda: Anomura: Paguroidea: Pylochelidae: Trizochelinae), description of the adult of one, and preliminary implications of development on pylochelid phylogeny. *Zootaxa* 1911: 52–68.

McLaughlin PA, Lemaitre R, Tudge CC (2004) Carcinization in the Anomura – fact or fiction? II. Evidence from larval, megalopal and early juvenile morphology. *Contributions to Zoology* 73: 165–205.

Muraoka K, Konishi K (1987) The first zoeal stage of the porcellanid crab, *Petrolisthes japonicus* (de Haan, 1849) with special reference to zoeal features of *Petrolisthes* (Crustacea: Anomura). *Researches on Crustacea* 16:57–65.

Nates S, Felder DL, Lemaitre R (1997) Comparative larval development in two species of the burrowing ghost shrimp genus *Lepidophthalmus* (Decapoda: Callianassidae). *Journal of Crustacean Biology* 17: 497–519.

Negreiros-Franzoso ML, Hebling NJ (1983) Desenvolvimento pós-embrionário de *Isocheles sawayai* Forest & Saint Laurent, 1967 (Decapoda, Diogenidae), em laboratório. *Papéis Avulsos de Zoologia, Museu de Zoologia da Universidade de São Paulo* 35: 41–53.

Ngoc-Ho N (1981) A taxonomic study of the larvae of four thalassinid species (Decapoda, Thalassinidea) from the Gulf of Mexico. Bulletin of the British Museum of Natural History (Zoology) 40: 237–273.

Nyblade CF (1970) Larval development of *Pagurus annulipes* (Stimpson, 1862) and *Pagurus pollicaris* Say, 1817 reared in the laboratory. *Biological Bulletin* 139: 557–573.

Nyblade CF, McLaughlin PA (1975) The larval development of *Labidochirus splendescens* (Owen, 1839) (Decapoda, Paguridae). Crustaceana 29: 271–289.

Pike RB, Wear RG (1969) Newly hatched larvae of the genera *Gastroptychus* and *Uroptychus* (Crustacea, Decapoda, Galatheidea) from New Zealand waters. *Transactions of the Royal Society of New Zealand, Biological Sciences* 11:189-195.

Provenzano AJ, Jr (1962) The larval development of *Calcinus tibicen* (Herbst) (Crustacea, Anomura) in the laboratory. *Biological Bulletin* 123: 179–201.

Provenzano AJ, Jr (1967) The zoeal stages and glaucothoe of the tropical Eastern Pacific hermit crab *Trizopagurus magnificus* (Bouvier, 1898) (Decapoda; Diogenidae), reared in the laboratory'. *Pacific Science* 21: 457–473.

Provenzano AJ, Jr (1968a) The complete larval development of the West Indian hermit crab *Petrochirus diogenes* (L.) (Decapoda, Diogenidae) reared in the laboratory. *Bulletin of Marine Science* 18: 143–181.

Provenzano AJ, Jr (1978) Larval development of the hermit crab, *Paguristes spinipes* Milne-Edwards, 1880 (Decapoda, Diogenidae) reared in the laboratory *Bulletin of Marine Science* 28: 512–526.

Reese ES, Kinzie RA, III (1968) The larval development of the Coconut Crab or Robber Crab *Birgus latro* (L.) in the laboratory (Anomura, Paguridea). *Crustaceana* Supplement 2: 117–144

Roberts PE (1973) Larvae of *Munida subrugosa* (White, 1847) from Perseverance Harbour, Campbell Island. *Journal of the Royal Society of New Zealand* 3: 393–408.

Saito T, Konishi K (2002) Description of the first stage zoea of the symmetrical hermit crab *Pylocheles mortensenii* (Boas, 1926) (Anomura, Paguridea, Pylochelidae). *Crustaceana* 75: 621–628.

Samuelsen TJ (1972) Larvae of *Munidopsis tridentata* (Esmark) (Decapoda, Anomura) reared in the laboratory. *Sarsia* 48: 91–98.

Sakai K, Miyake S (1964) Description of the first zoea of *Laomedia astacina* de Haan (Decapoda, Crustacea). *Science Bulletin of the Faculty of Agriculture, Kyushu University* 21: 83–87.

Sandifer PA (1974) Larval stages of the shrimp, *Ogyrides limicola* Williams, 1955 (Decapoda, Caridea) obtained in the laboratory. *Crustaceana* 26: 37–60.

Scelzo MA, Fantucci MZ, Mantelatto FL (2010) Spermatophore and gonopore morphology of the southwestern-Atlantic hermit crab *Pagurus exilis* (Benedict, 1892) (Anomura, Paguridae). *Zoological Studies* 49: 421–433.

Scholtz G, Richter S (1995) Phylogenetic systematics of the reptantian Decapoda (Crustacea, Malacostraca). *Zoological Journal of the Linnean Society* 113: 289–328.

Shy J-Y, Chan T-Y (1996) Complete larval development of the edible mud shrimp *Upogebia edulis* Ngoc-Ho & Chan, 1992 (Decapoda, Thalassinidea, Upogebiidae) reared in the laboratory. *Crustaceana* 69: 175–186.

Strasser KM, Felder DL (2000) Larval development of the ghost shrimp *Callichirus islagrande* (Decapoda: Thalassinidea: Callianassidae) under laboratory conditions. *Journal of Crustacean Biology* 20: 100–117.

Stuck KC, Truesdale FM (1986) Larval and early postlarval development of *Lepidopa benedicti* Schmitt, 1935 (Anomura: Albuneidae) reared in the laboratory. *Journal of Crustacean Biology* 6: 89–110.

Tirelli T, Silvestro D, Pessani D, Tudge CC (2010) Description of the male reproductive system of *Paguristes eremita* (Anomura, Diogenidae) and its placement in a phylogeny of diogenid species based on spermatozoal and spermatophore ultrastructure. *Zoologischer Anzeiger* 248: 299–312.

Tudge CC (1995) Ultrastructure and phylogeny of the spermatozoa of the infraorders Thalassinidea and Anomura (Decapoda, Crustacea). In: BG Jamiseson, J Ausio, JL Justine (eds) Advances in Spermatozoal Phylogeny and Taxonomy. *Mémoires du Muséum national d'Histoire naturelle* 166: 251–263.

Tudge CC (1997) Phylogeny of the Anomura (Decapoda, Crustacea): spermatozoa and spermatophore morphological evidence. *Contributions to Zoology* 67: 125–141.

Tudge CC (2003) Endemic and enigmatic: the reproductive biology of Aegla (Crustacea: Anomura: Aeglidae) with observations on sperm structure. *Memoirs of Museum Victoria* 60: 63–70.

Tudge CC, Scheltinga DM, Jamieson BG (2001) Spermatozoal morphology in the "symmetrical" hermit crab, *Pylocheles* (*Bathycheles*) sp. (Crustacea, Decapoda, Anomura, Paguroidea, Pylochelidae). *Zoosystema* 23: 117–130.

Tudge CC, Scheltinga DM (2002) Spermatozoal morphology of the freshwater anomuran *Aegla longirostri* Bond-Buckup & Buckup, 1994 (Crustacea: Decapoda: Aeglidae) from South America. *Proceedings of the Biological Society of Washington* 115: 118–128.

Tudge CC, Jamieson BG (1996b) Spermatophore and spermatozoa morphology in the Porcellanidae. II. The genera *Petrolisthes* and *Polyonyx* (Decapoda, Anomura, Porcellanidae). *Journal of Crustacean Biology* 16: 535–546.

Tudge CC, Jamieson BG (1996a) Spermatophore and spermatozoa morphology in the Porcellanidae. I. *Aliaporcellana suluensis* and *Pisidia longicornis* (Decapoda, Anomura, Porcellanidae). *Crustacean Research* 25: 73–85.

Van Dover CL (1982) Reduction of maxillary endites in larval Anomura and Brachyura. *Crustaceana* 43: 211–215.

Van Dover CL, Factor JR, Gore RH (1982) Developmental patterns of larval scaphognathites: and aid to the classification of Anomuran and Brachyuran Crustacea. *Journal of Crustacean Biology* 2: 48–53.

Wilkens H, Parzefall J, Ribowski A (1990) Population biology and larvae of the anchialine crab *Munidopsis polymorpha* (Galatheidae) from Lanzarote (Canary Islands). *Journal of Crustacean Biology* 10: 667–675.

Yang HJ (2005) Larval development of *Latreutes anoplonyx* (Decapoda: Hippolytidae) reared in the laboratory. *Journal of Crustacean Biology* 25: 462–479.
